# Identification and Exploration of Immunity‐Related Genes and Natural Products for Alzheimer's Disease Based on Bioinformatics, Molecular Docking, and Molecular Dynamics

**DOI:** 10.1002/iid3.70166

**Published:** 2025-04-07

**Authors:** Pengpeng Liang, Yale Wang, Jiamin Liu, Hai Huang, Yue Li, Jinhua Kang, Guiyun Li, Hongyan Wu

**Affiliations:** ^1^ Shenzhen Hospital Shanghai University of Traditional Chinese Medicine Shenzhen China; ^2^ Shenzhen Longgang Second People's Hospital Shenzhen China

**Keywords:** Alzheimer's disease, bioinformatics, biomarkers, Chinese herbs, dynamics simulation, immune infiltration, machine learning, molecular docking, natrual compounds

## Abstract

**Background:**

Recent research highlights the immune system's role in AD pathogenesis and promising prospects of natural compounds in treatment. This study explores immunity‐related biomarkers and potential natural products using bioinformatics, machine learning, molecular docking, and kinetic simulation.

**Methods:**

Differentially expressed genes (DEGs) in AD were analyzed using GSE5281 and GSE132903 datasets. Important AD module genes were identified using a weighted co‐expression algorithm (WGCNA), and immune‐related genes (IRGs) were obtained from the ImmPortPortal database. Intersecting these genes yielded important IRGs. Then, the least absolute shrinkage and selection operator (LASSO) and other methods screened common immune‐related AD markers. Biological pathways were explored through Gene Ontology (GO), Kyoto Encyclopedia of Genes and Genomes (KEGG), and Gene Set Enrichment Analysis (GSEA). The accuracy of these markers was assessed by subject operator signature (ROC) curves and validated in the GSE122063 dataset. The datasets was then subjected to immunoinfiltration analysis. Multiple compound databases were used to analyze core Chinese medicines and components. Molecular docking and kinetic simulation verification were used for further verification.

**Results:**

A total of 1360 differential genes and 5 biomarkers (PGF, GFAP, GPI, SST, NFKBIA) were identified, showing excellent diagnostic efficiency. GSEA revealed markers associated with Oxidative phosphorylation, Nicotine addiction, and Hippo signaling pathway. Immune infiltration analysis showed dysregulation in multiple immune cell types in AD brains, with significant interactions between markers and 5 immune cell types. A total of 27 possible herbs and 7 core compounds were eventually identified. The binding environment of GPI‐luteolin and GPI‐stigasterol was relatively stable and showed good affinity.

**Conclusions:**

PGF, GFAP, SST, GPI, and NFKBIA were identified for early AD diagnosis, associated with immune cells and pathways in AD brains. 7 promising natural compounds, including luteolin and stigmasterol, were screened for targeting these biomarkers.

## Graphical abstract

We used a variety of methods to identify biomarkers and compounds of Alzheimer's disease, providing new insights into the clinical research transformation of Alzheimer's disease.

**Abbreviations**
AD: Alzheimer's disease
AUC: area under the receiver operating characteristic Curve
BP: biological process
CC: cellular component
CIBERSORT: robust enumeration of cell subsets from tissue expression profiles
DCA: decision curve analysis
DCCM: dynamic covariance matrix
DEGs: differential genes
GEO: Gene Expression Omnibus
GO: Gene Ontology
GSEA: gene set enrichment analysis
GV‐971: Manamide sodium capsule
HK: hexokinase
ICDs: immunogenic cell death genes
IIRGs: important immunity‐related genes
ImmPort: immunology database and analysis portal
IRGs: immune‐related genes
KEGG: Kyoto Encyclopedia of Genes and Genomes
LASSO: least absolute shrinkage and selection operator
MD: molecular dynamics
MF: molecular function
MLP: multilayer perceptron
MM‐PBSA: molecular mechanics Poisson‐Boltzmann surface area
PCA: principal component analysis
PFK‐1: phosphofructokinase‐1
PK: pyruvate kinase
PME: Particle‐Mesh‐Ewald
RF: random forest
RG: radius of gyration
RMSD: root mean square deviation
RMSF: root mean square fluctuation
ROC: receiver operator characteristic curve
SASA: solvent‐accessible surface area
ssGSEA: single sample gene set enrichment analysis
STZ: Streptozotocin
SVM‐RFE: support vector machine‐recursive feature elimination
TCM: traditional Chinese medicine
TOM: topological overlap matrix
WGCNA: weighted gene co‐expression network analysis
WHO: World Health Organization
XGBOOST: extreme gradient boosting

## 1 Introduction

As the population ages, the prevalence of neurodegenerative disorders is rising in tandem, posing a global public health challenge [1]. Alzheimer's disease (AD) is a degenerative brain condition that often affects older individuals, leading to cognitive decline, memory loss, language difficulties, visual‐spatial issues, and changes in behavior and personality, significantly impacting their quality of life. A World Health Organization (WHO) report states that there are approximately 50 million individuals with AD globally, with projections indicating a rise to 152 million by 2050, with an economic impact expected to surpass $900 million [2]. However, the exact pathological mechanism of AD remains unclear. Current theories suggest that AD development is linked to senile plaques, neurofibrillary tangles, and the death of neural cells [3]. To date, all drugs for AD, such as memantine, donepezil, and rivastigmine, can only alleviate the patient's clinical symptoms but can't fundamentally reverse the pathological process of AD. Long‐term combined use of the above drugs may cause dizziness, nausea, diarrhea, hepatorenal toxicity, and other adverse reactions in patients. Moreover, a significant number of individuals with AD may not exhibit any signs in the initial years before the development of dementia, leading to delays in identifying the condition and determining the best time for treatment [4, 5]. As a result, it is crucial to thoroughly examine and evaluate genes and markers from various angles and investigate potential medications to enhance existing strategies for preventing and treating AD.

In recent years, mounting evidence suggests that the immune response plays a critical role in both the initiation and development of AD [6, 7, 8, 9]. The main cell types involved in immune response are microglia, macrophages, mast cells and T cells. Microglia, a distinctive type of immune cells residing in the central nervous system, are primarily tasked with initiating the inflammatory response in the brain [10, 11]. M1 microglia exhibit pro‐inflammatory characteristics and release pro‐inflammatory molecules that can worsen neuronal harm, including IL‐1β, IL‐6, and TNF‐α. In contrast, M2 microglial cells have the ability to control the brain environment and enhance tissue healing through the secretion of anti‐inflammatory substances and certain neurotrophic factors [12]. Activation of mast cells by Aβ protein can lead to the release of preformed and newly synthesized inflammatory mediators, increasing blood‐brain barrier permeability. This process also includes the recruitment of T‐cells and macrophages into the brain tissue [13]. Macrophages, a type of immune cell known for their strong ability to engulf particles, play a role in controlling inflammation and removing Aβ/Tau plaques [14]. CD4+ T cells are one of the main T cell types contribute to AD progression. Th1 and Th17 cells significant stimulate the pro‐inflammatory reaction of microglia, which is also partially influenced by Th2 cells [15]. These findings indicate that comprehending immune responses linked to AD pathology is crucial for treating and preventing AD. Therefore, managing the brain's immune system, enhancing brain metabolism, and removing pathological indicators like Aβ and Tau through clinical interventions are anticipated to be among the most effective treatment approaches for AD.

Despite clinical efforts, there has been little advancement in the research and creation of novel medications for AD over the last few decades [16]. Recently, Manamide sodium capsule (GV‐971), independently developed by China, can improve the gut microflora, reduce the content of phenylalanine and leucine, reduce neuroinflammation in the brain, and improve cognition [17]. Although Phase 3 clinical trials have confirmed that it has a certain effect on improving cognition [18], due to the epidemic and financial reasons, the study was terminated early in 2022. In addition, more detailed experimental data related to Aβ deposition and its molecular mechanism are still lacking. Natural products and extracts have shown increased effectiveness in preventing and treating neurodegenerative diseases like Alzheimer's and Parkinson's due to their multi‐target, multi‐pathway, gentle action, and minimal side effects in recent years. Several herbal extracts have been found to have good pharmacological effects on neurodegenerative diseases. Ginsenoside Rg1 primarily functions as a neuroprotectant by reducing nerve damage and cognitive impairment in AD through its antioxidant, anti‐inflammatory, and anti‐apoptotic properties [19]. Angelica polysaccharide has the ability to boost neuron count in the CA1, CA3, and DG areas of the hippocampus in rats with AD, triggering the BDNF/TrkB/CREB pathway to suppress the production of inflammatory cytokines, ultimately enhancing memory function in these rats [20]. Some of classic formulas, such as Kaixin Powder, have been extensively utilized for treating dementia with positive results [21, 22, 23], suggesting that medicinal herbs and their components could be beneficial resources for developing new drugs for AD. While many researchers have reviewed studies and animal experiments to explore the potential of Chinese herbs and natural compounds in immune‐based treatment for AD, there is a lack of systematic research on herbs and compounds that target immune‐related factors in AD. Furthermore, the interaction between important compounds and key immune targets still requires further investigation and clarification.

**Figure 1:**
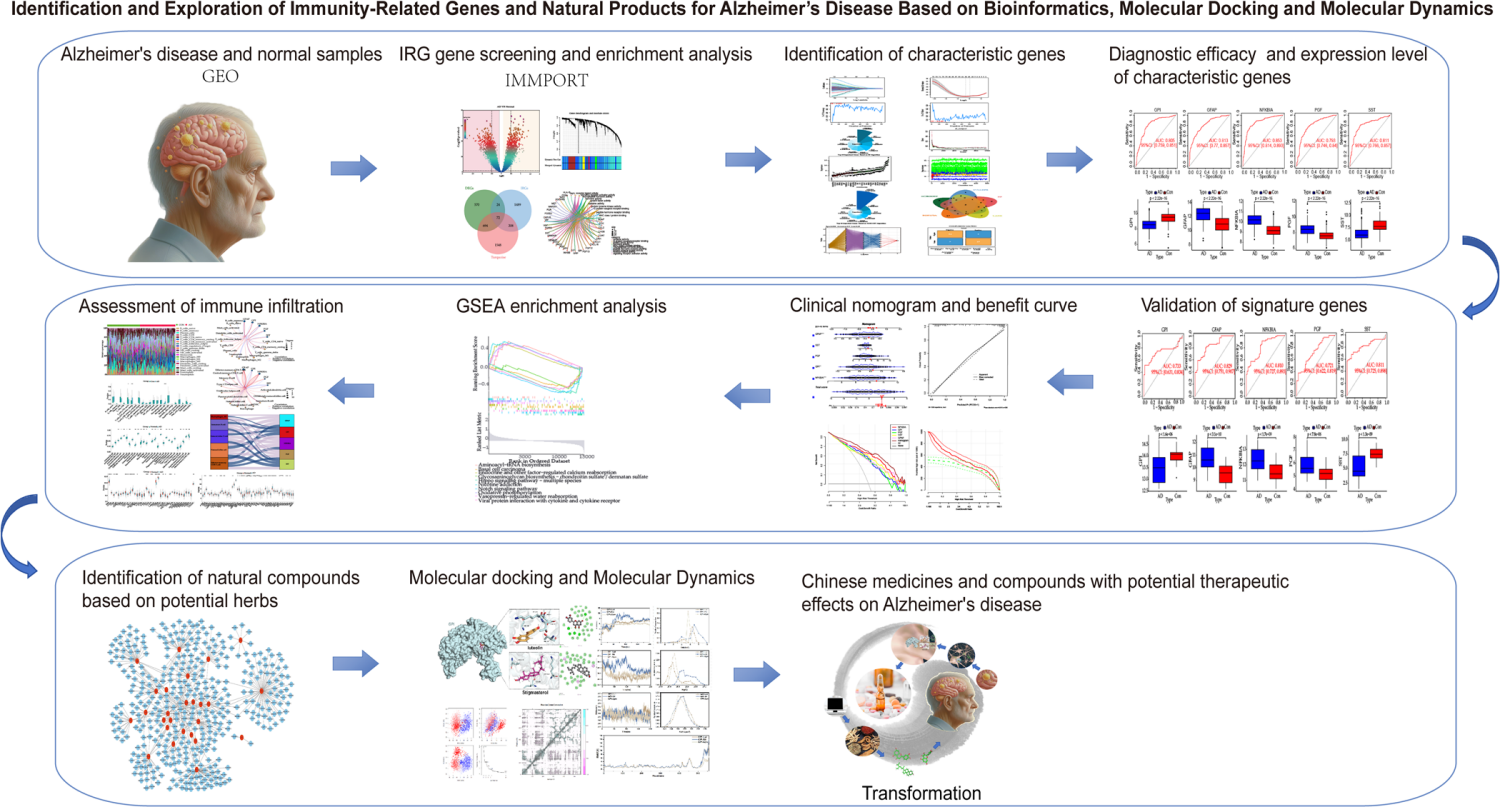
Flow chart of this study. The datasets were acquired from the GEO database and integrated with an immune‐related gene set. Subsequently, differential gene analysis, machine learning techniques, clinical predictive models, immune infiltration assessments, traditional Chinese medicine compound predictions, molecular docking studies, and dynamic simulations were conducted to provide new insights into the development of Alzheimer's disease markers to novel drugs.

In this study, we utilized bioinformatics, machine learning, molecular docking, and kinetic simulation technologies to address current research challenges. From an immunological perspective, we not only systematically analyzed the biological functional differences and biomarkers between normal and AD individuals but also constructed clinical prediction models and curves. Furthermore, we evaluated the extent of immune infiltration and examined variations in immune cell types among individuals with AD, and their correlation with immune markers. Finally, we utilized multiple herbal databases to screen potentially effective traditional Chinese medicines (TCMs) and compounds while validating the interactions between key compounds and disease targets through simulation. The research process is depicted in Figure 1. This study provides a solid foundation for the exploration of mechanisms, clinical interventions, and the development of novel pharmaceuticals.

## 2 Materials and Methods

### 2.1 Downloading and Processing of Data and Acquisition of Immune‐Related Genes (IRGs)

We obtained three raw AD microarray datasets (GSE132903, GSE5281, and GSE122063) from the GEO database. Each dataset's probes were annotated and converted into gene symbols using platform annotation files. Probes without matching gene symbols were excluded from further analysis. In cases where multiple probes mapped to the same gene, the probe with the highest average expression level was chosen to represent gene expression. The GSE5281 and GSE132903 datasets were combined into a training set after correcting for batch effects using the Combat function from the “sva” package [24]. Additionally, the gene expression data from the GSE122063 AD dataset served as the validation dataset for subsequent analysis. We downloaded a list of 1793 immune‐related genes (IRGs) from ImmPort, excluding duplicates, for further investigation.

### 2.2 Analysis of Differential Expression Genes (DEGs)

Following normalization of the data with the “Normalize Between Arrays” function in the “limma” R package [25], DEGs between AD and control samples were analyzed using the same “limma” R package. DEGs were identified based on criteria of |log2 fold change (log2FC)| > 0.5 and *p* < 0.05. Subsequently, volcano plots and heatmaps were generated using the “ggplot2” and “ComplexHeatmap” R packages, respectively [26].

### 2.3 Identification of Co‐Expression Genes

To comprehensively understand the genetic mechanisms underlying AD development and screened the important modules of differential gene co‐expression, we constructed a co‐expression network using the “WGCNA” package in R [27]. The network was built using the top 5000 most variant genes from the merged dataset based on variance. Initially, we transformed the adjacency matrix into a topological overlap matrix (TOM). Hierarchical clustering was then applied to detect modules within the network, and eigengenes were computed for each module. Subsequently, we evaluated the correlation between phenotype (AD vs. Normal) and each module using Pearson's correlation analysis, identifying modules associated with AD for further marker screening.

### 2.4 Identification of Important Immunity‐Related Hub Genes (IIRGs) and Functional Enrichment Analysis

We intersected the DEGs, the module signature genes in WGCNA, and immunity‐related genes to identify IIRGs. A Venn diagram was used to describe the core components of the genes. The identified Gene Ontology (GO) terms and Kyoto Encyclopedia of Genes and Genomes (KEGG) pathways in AD or normal tissues can reflect the AD related specific gene expression pattern and its related biological processes. Thus we further performed the biological processes and enrichment pathways of of IIRGs based on the GO (http://geneontology.org/page/go-enrichment-analyses) and KEGG (http://www.genome.jp/kegg/pathway.html). Next, we used bioinformatics platform (https://www.bioinformatics.com.cn) for the visualization analysis.

### 2.5 Machine Learning for the Diagnostic Feature Genes

Faced with complex biological data, machine learning gives biomarker screening highly automated, efficient, and accurate capabilities, making it an important tool in biomedical research.To improve the accuracy of screening genes, we used six machine learning methods, LASSO, SVM‐RFE, RF, BORUTA, XGboost, and MLP. The machine learning models integrate genes into training and test sets to construct and evaluate the models. Additionally, GSE122063 is utilized as an independent validation set to assess the models’ performance in real‐world applications. LASSO was utilized to identify the core feature genes using the “glmnet” R package [28]. According to the minimum binomial deviance, we can select the best eigenvalue λ to determine the characteristic genes. SVM‐RFE was used to identify the genes using “e1071” R packages [29], the number of features is determined according to the highest accuracy and minimum error in the SVM‐RFE model. RF is an ensemble technique that combines the results of multiple decision trees to improve performance, implemented using “randomforest” R packages. The top 10 genes were identified according to the “importance” parameters in the RF model. Boruta is a feature selection method that effectively selects relevant features based on random forest, implemented using “Boruta” R packages. The Boruta model classified the included candidate genes as confirmed, rejected, and tentative. We finally selected genes with confirmed tags as characteristic genes for further analysis. XGBoost is an efficient gradient lifting decision tree algorithm that can handle many problems, implemented using “xgboost” R packages. The top 10 genes were identified according to the “importance” parameters in the “xgboost” model. The common genes identified by five methods exhibited high accuracy and were recognized as crucial genes. Finally, we took the crucial genes as neurons and constructed multi‐layer perceptron and confusion matrix based on “Keras” and “TensorFlow” R packages to verify the accuracy of the model by using confusion matrix and related parameters.

### 2.6 Assessment of the Diagnostic Value of Candidate Biomarkers in AD and Gene Set Enrichment Analysis (GSEA)

We plotted the receiver operating curve (ROC) analysis and utilized the area under the receiver operating characteristic curve (AUC) to evaluate the diagnostic effectiveness of each hub gene using the “pROC” package [30]. The external validation dataset GSE122063 was used to verify candidate biomarkers’ expressions and diagnostic values. Finally, a nomogram was established for comprehensive risk score evaluation by using the “rms” R package. Meanwhile, the calibration curve was used to evaluate the predictive ability of the nomogram model. In addition, decision curve analysis and clinical impact curves were plotted to evaluate the model's utility value by using the “rmda” R package. GSEA enrichment analysis was implemented, and related maps were implemented to functionally elucidate the biological significance of characteristic genes using the “clusterProfiler” R package [31].

### 2.7 Validation of Characteristic Genes Based on Bioinformatics Analysis

We first employed additional datasets GSE122063 to evaluate the ROC diagnostic efficiency and expression levels of five key markers selected by machine learning, comparing the overall results with those from the training set. Subsequently, PC12 neuron cells were obtained from Abiowell and cultured in RPMI‐1640 medium with 5% fetal bovine serum, 5% fetal bovine serum, 10% Horse serum, and appropriate amounts of penicillin and streptomycin at 37°C and 5% CO2. We induced PC12 cells with Aβ1‐42 for 12 h and the final concentration of Aβ142 was 7 μM to detect the expression levels of five hub genes using real‐time fluorescent quantitative PCR [32, 33].

### 2.8 Evaluation of Immune Infiltration, Immune Checkpoints, and Immunogenic Cell Death (ICD) Related Genes

Immunoinfiltration analysis, immune checkpoints, and ICD related genes provide insights into the pathological progression of AD. These mechanisms also serve as potential therapeutic targets, facilitating early detection and accurate diagnosis of the disease course in Alzheimer's patients. The abundance and correlation of immune infiltrating cells were analyzed using the Cibersort deconvolution algorithm and ssGSEA. The Cibersort method mainly relies on the LM22 immune cell subtype expression matrix, which is analyzed qualitatively and quantitatively using the “Cibersort” R package [34]. The ssGSEA algorithm obtains 28 immune‐related gene sets based on the TISIDB website and uses the “GSVA” R package for abundance calculation [35]. In addition, we compared the expression of 37 common immune checkpoints and 53 immune ICD genes in the normal and AD groups by the Wilcoxon rank sum test. Based on the results of two immune infiltration analysis algorithms, we used the Spearman method for correlation analysis via the CNSknownall website (https://cnsknowall.com/#/Home/associationNetwork?pid=21001016). Then the regulatory networks between hub genes and immune cells were mapped. According to the overlapping cells shared between the two algorithms, we further summarized the correlation results of immune cells for each immune marker (*p* < 0.05), identified common immune cells, and drew the correlation Sankey map with the help of the CNSknowall platform.

### 2.9 Identification of Small Molecular Therapeutic Agents

The TCM database is essential for research on Chinese herbal medicine, as it systematically integrates data on chemical components, pharmacological effects, and clinical applications. Additionally, it provides scientific evidence to support drug development and initiatives for the modernization of TCM. We extensively searched three major medical databases (Herb, Coremine, and Symmap) to identify herbal medicines with potential therapeutic effects for AD. The search results were then cross‐referenced and the Chinese medicines found in these databases were identified as important Chinese medicines. Following OB > 30 and DL > 0.18 criteria, we analyzed these key TCMs using the TCMSP database to determine their potential compound components. Subsequently, we used the Cytoscape software to construct a network of ingredients from TCM. The Network Analyzer is utilized to conduct visual analysis.

### 2.10 Molecular Docking and Molecular Dynamics (MD) Simulation

To further explore the molecular interactions between core targets and natural compounds, molecular docking studies were conducted using Autodock Vina 1.2.2 software to assess the binding affinities between potential ligands and target proteins. The crystal structures of the target proteins were obtained from the RCSB PDB database (http://www.rcsb.org/ [accessed on 31 MAY 2024]), while the 3D structures of the ligands were downloaded from the PubChem library (https://pubchem.ncbi.nlm.nih.gov/). Before docking analysis, all water molecules were removed and hydrogen atoms were added to both protein and ligand files, which were then saved in PDBQT format. The grid box encompassed the entire receptor protein to allow unrestricted movement of the ligand, with the protein's active site designated as the docking site. Docking simulations were executed using Autodock Vina 1.2.2 (http://autodock.scripps.edu/), and the highest scoring result was selected as the final outcome following completion of the docking process. Subsequently, visual inspection of the docking results and additional analyses were performed using Discovery Studio Visualizer v.19.

We used the PMEMD module of Amber 22 [36, 37] to simulate the MD of a CUDA‐accelerated protein‐molecular complex system with the top two affinity. Each system was simulated for 100 ns. The SHAKE algorithm constrained hydrogen bonds [38], and the Particle‐Mesh‐Ewald (PME) method managed electrostatic interactions with a truncation radius of 8 Å [39]. Following initial system setup, atomic clashes were reduced through a 500‐step steepest descent method, followed by conjugate gradient minimization. Subsequently, the system underwent heating from 0 to 300 K over 50 ps, followed by 500 ps of density equilibration and constant pressure operations in the NPT ensemble at 300 K. After stabilization, four independent 200‐ns MD simulations were conducted using Langevin thermostats [40], employing a time step of 2 fs and collision frequency of 1 ps. Data were recorded every 1 fs and stored every 2 ps, with 2000 frames selected for subsequent analysis. The trajectories were analyzed using Amber 22's CPPTRAJ module [41] to compute root mean square deviation (RMSD), root mean square fluctuation (RMSF), radius of gyration (Rg), and solvent‐accessible surface area (SASA). Additionally, the difference in binding free energy of the protein‐ligand complex was assessed using molecular mechanics Poisson‐Boltzmann surface area (MM‐PBSA) based on 500 snapshots extracted from the final trajectory [42, 43, 44, 45]. Additionally, we used the AMBER tool for principal component analysis (PCA) and extracting eigenvectors. These eigenvectors were used to project conformational changes in the proteins, providing insight into the major movements in the MD simulations. To analyze the covariance matrix of proteins, we used the “Bio3D” package in R [46, 47]. The dynamic covariance matrix (DCCM) was visualized to explain the motion correlation between different parts of the protein.

## 3 Results

### 3.1 Identification of DEGs in AD Brain Tissues

**Figure 2:**
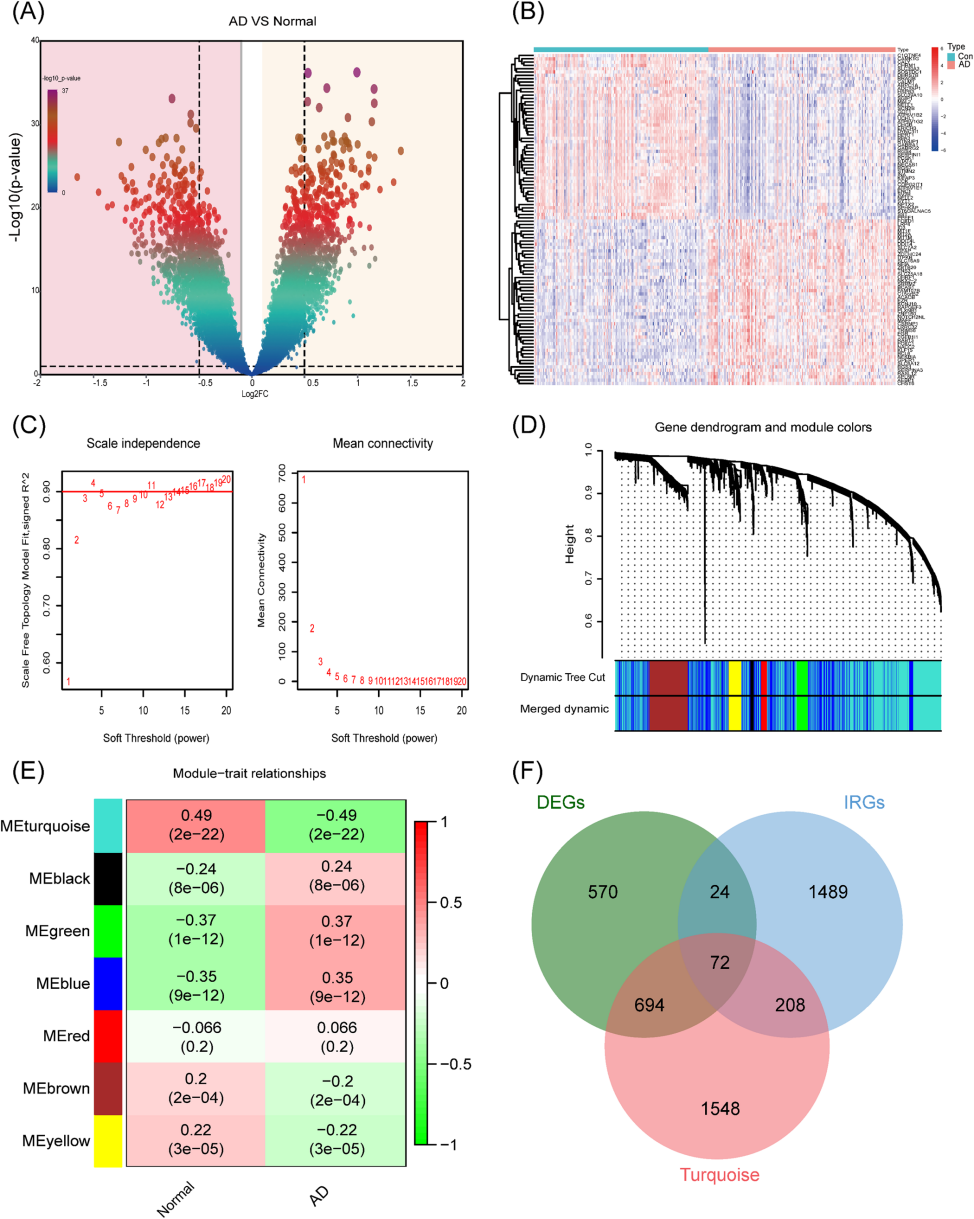
Identification of IIRGs in Alzheimer's disease brain tissues. (A) Volcano map. (B) Heatmap. (C) The soft threshold power and mean connectivity of WGCNA. (D) The cluster dendrogram of WGCNA. (E) Correlations between gene modules and AD status. (F) Venn diagram of the Turquoise module, DEGs, IRGs.

Following preprocessing and elimination of batch effects, we identified 1360 DEGs with a |log2FC| > 0.5 and *p* < 0.05, consisting of 612 genes that were upregulated and 748 genes that were downregulated (Figure 2A). The heatmap illustrated the summary of the distinctive gene expression (Figure 2B).

### 3.2 Identification of IIRGs in AD Brain Tissues

We further performed weighted coexpression analysis of the above differential genes to explore important gene expression modules. We obtained the network structure using a scale‐free fitting index of 0.9 and a soft threshold value of 3 (Figure 2C,D). The top 5000 genes with the highest variation were clustered and combined into 7 co‐expression modules. Pearson correlation analysis explored the correlation between module feature genes and clinical traits (Figure 2E). The results showed that each module had a general correlation with clinical features, and the turquoise module had a higher correlation than other modules (cor = 0.49, *p* < 0.01). In addition, turquoise module gene significance and module membership analysis showed a high correlation, as seen in the Supporting Information Materials “WGCNA” folder (cor = 0.83, *p* < 0.01). Therefore, this module was selected as an important module, and 2552 genes were used for subsequent analysis. We take the intersection of the turquoise module, DEGs, and IRGs, where in the cross‐region genes are selected as IIRGs. A total of 72 hub genes were finally identified for subsequent analysis (Figure 2F, Table 1).

**Table 1:** The list of characteristic genes screened by five kinds of machine learning.

Machine learning	Screening criteria	Characteristic genes
LASSO	The minimum binomial deviance and the best eigenvalue λ	APLNR, BDNF, BST2, CCK, CCL2, CD8A, CSRP1, FGF12, FGF13, GFAP, GPI, HDAC1, IFITM1, IL13RA2, IL17RD, IL9, LEAP2, MAP2K1, NFKB1, NFKBIA, NFKBIZ, PGF, PLXNB1, PPP3CB, RASGRP3, RBP4, ROBO2, SCG2, SST, TAC1, VGF, HLA‐E
SVM‐RFE	The highest accuracy and minimum error	NFKBIA, GFAP, GPI, RBP4, HDAC1, CSRP1, IFITM1, FGF12, SCG2, LEAP2, PLXNB1, PGF, SST, NFKBIZ, BDNF, SEMA3F, CHGB, IL4R
RF	Ranked top 10 in importance in the RF model	ANXA6, FGR, TAP1, CHGB, PLXNB1, GFAP, PGF, GPI, SST, NFKBIA
BORUTA	Genes with confirmed tags	ADIPOR2, AHNAK, ANXA6, APLNR, BDNF, CCK, CHGB, CORT, CRH, CSRP1, CXCL1, FGF12, FGF13, FGR, GFAP, GPER1, GPI, IL17RD, LEAP2, NDRG1, NFKB1, NFKBIA, NFKBIZ, PAK1, PCSK1, PDGFRB, PGF, PLXNB1, PMP2, PPP3R1, PTH1R, PTH2R, RASGRP3, RBP4, ROBO3, SERPINA3, SST, TAP1, TUBB3, VGF, HLA‐E
XGBOOST	Ranked top 10 in importance in the XGBOOST model	NFKBIA, GPI3, PGF, SST, GFAP, LEAP2, FGF13, BST2, CCL2, IL4R
IIRGS	The overlap of three datasets DEGs, IRGs, and Turquoise.	ADIPOR2, AHNAK, ANXA6, APLNR, BDNF, BST2, CCK, CCL2, CD8A, CHGB, CORT, CRH, CSF1R, CSRP1, CXCL1, CXCL16, FGF12, FGF13, FGFR3, FGR, GFAP, GPER1, GPI, GRP, HDAC1, HSP90AB1, IFITM1, IL13RA2, IL17RD, IL4R, IL9, INHBB, IRF7, LEAP2, MAP2K1, MET, NDRG1, NFKB1, NFKBIA, NFKBIZ, NPY, PAK1, PCSK1, PDGFRB, PGF, PLXNB1, PMP2, PNOC, PPP3CB, PPP3R1, PTH1R, PTH2R, RASGRP3, RBP4, ROBO2, ROBO3, SCG2, SEMA3F, SEMA5B, SERPINA3, SST, TAC1, TAP1, TGFB3, TNFRSF1A, TNFRSF1B, TPM2, TUBB3, VCAM1, VGF, VIP, HLA‐E

### 3.3 Functional Enrichment Analysis of IIRGs

**Figure 3:**
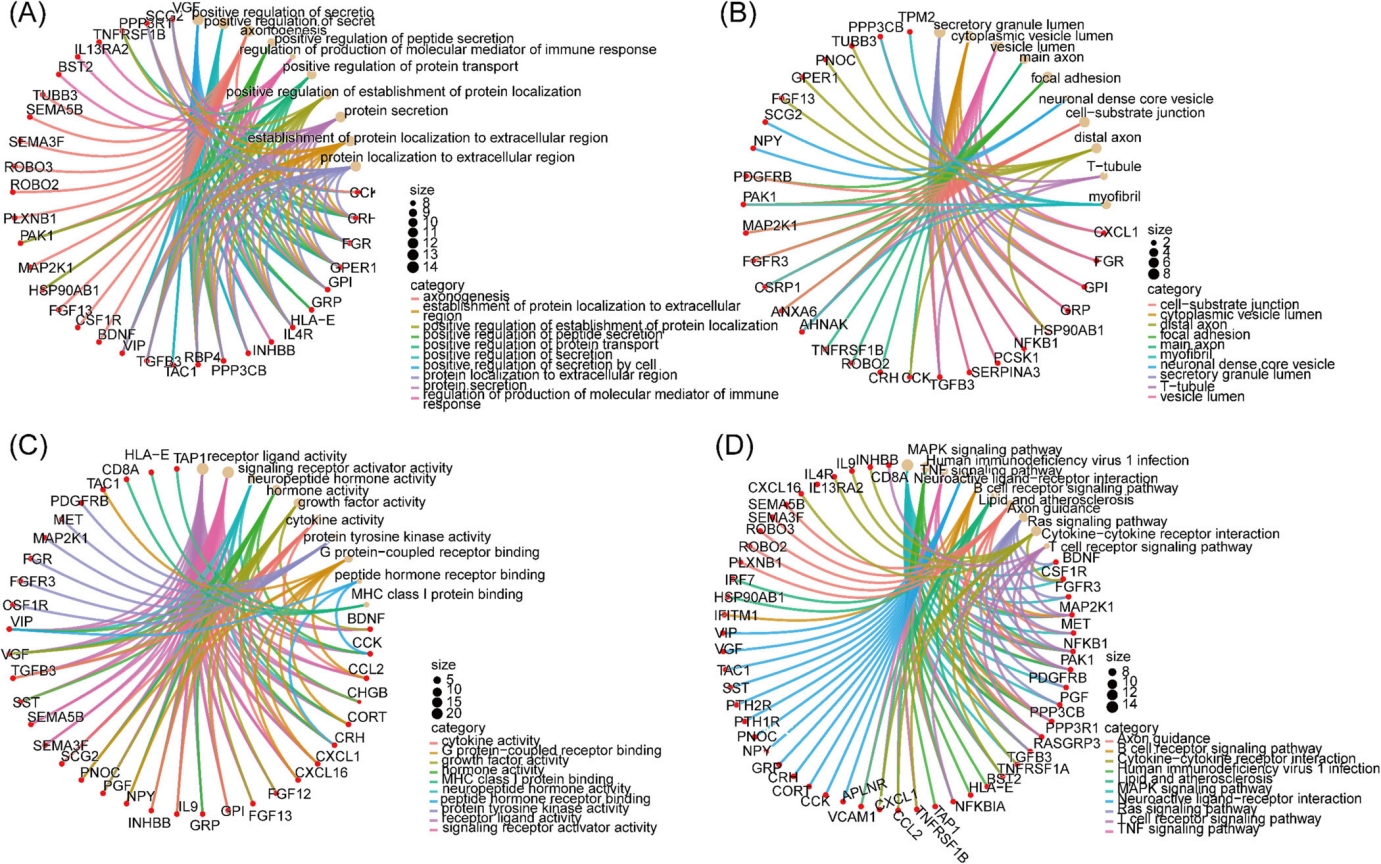
Functional enrichment analysis of IIRGS (A) GO functional enrichment analysis (BP). (B) GO functional enrichment analysis (CC). (C) GO functional enrichment analysis (MF). (D) KEGG pathway enrichment analysis.

Figure 3 depicts these genes' important GO functional terms, encompassing BP, MF, and CC. The significant GO‐BP terms (Figure 3A) were primarily associated with enhancing cell secretion, promoting secretion, axon formation, enhancing peptide secretion, and regulating the production of immune response mediators. GO‐CC (Figure 3B) suggested that crucial genes may be linked to the lumen of secretory granules, cytoplasmic vesicles, vesicles, main axons, and focal adhesions. In GO‐MF enrichment analysis (Figure 3C), the findings indicated participation in diverse functions, encompassing receptor‐ligand activity, signaling receptor activator activity, neuropeptide hormone activity, hormone activity, and growth factor activity. In KEGG analysis (Figure 3D), the most notably enriched pathways included the MAPK signaling pathway, human immunodeficiency virus 1 infection, TNF signaling pathway, Neuroactive ligand‐receptor interaction, and B cell receptor signaling pathway.

### 3.4 Discovery of Immune‐Related Diagnostic Biomarkers in AD

**Figure 4:**
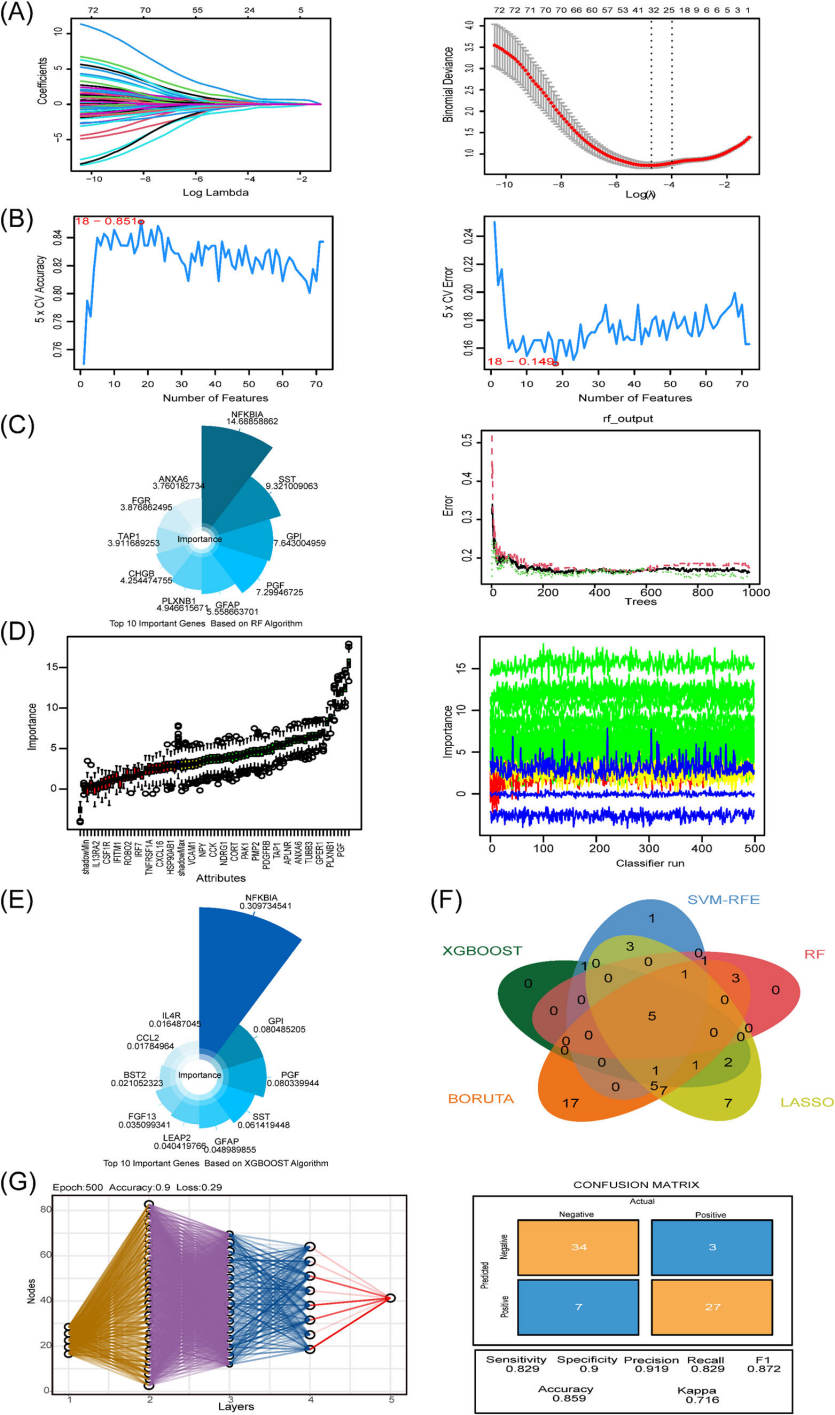
Identification of immune‐related diagnostic feature biomarkers in Alzheimer's disease. (A) The results of LASSO regression analysis. (B) The results of SVM‐RFE analysis. (C) The results of RF analysis. (D) The results of BORUTA analysis. (E) The results of XGBOOST analysis. (F) The intersection of the above five machine learning methods. (G) Neuron model based on five hub genes.

To accurately identify potential markers in the immune‐related gene set (72 genes) and reduce errors, we conducted five machine learning methods, including LASSO, SVM‐RFE, RF, BORUTA, and XGBOOST, to study the potential biomarkers of AD. Subsequently, we used a multi‐layer perceptron to construct a model to verify the accuracy of gene recognition. Utilizing the LASSO regression model, 32 characteristic genes associated with AD were identified from a pool of 72 hub genes (Figure 4A, Table 1). The SVM‐RFE algorithm identified 18 genes as possible diagnostic biomarkers (Figure 4B, Table 1). According to the ranking results of the importance of random forest genes, we selected the top 10 genes as candidate markers (Figure 4C, Table 1). Based on the BORUTA algorithm, we selected the candidate genes with “Confirmed” label in the analysis results (Figure 4D, Table 1), and XGBOOST, we also selected the top 10 important genes as candidate genes (Figure 4E, Table 1). A Venn diagram intersected the five algorithms, and five common genes were obtained as the characteristic genes closely related to AD (Figure 4F). The multi‐layer perceptron uses the above five genes as neurons and randomly divides the samples into the training set and the verification set according to the ratio of 8:2. After training the samples 500 times, the neuron model is obtained with an accuracy of 85%, indicating that the gene‐based model has robust performance and high diagnostic recognition ability (Figure 4G).

### 3.5 Evaluation and Validation of Diagnostic Effects of Characteristic Diagnostic Markers

**Figure 5:**
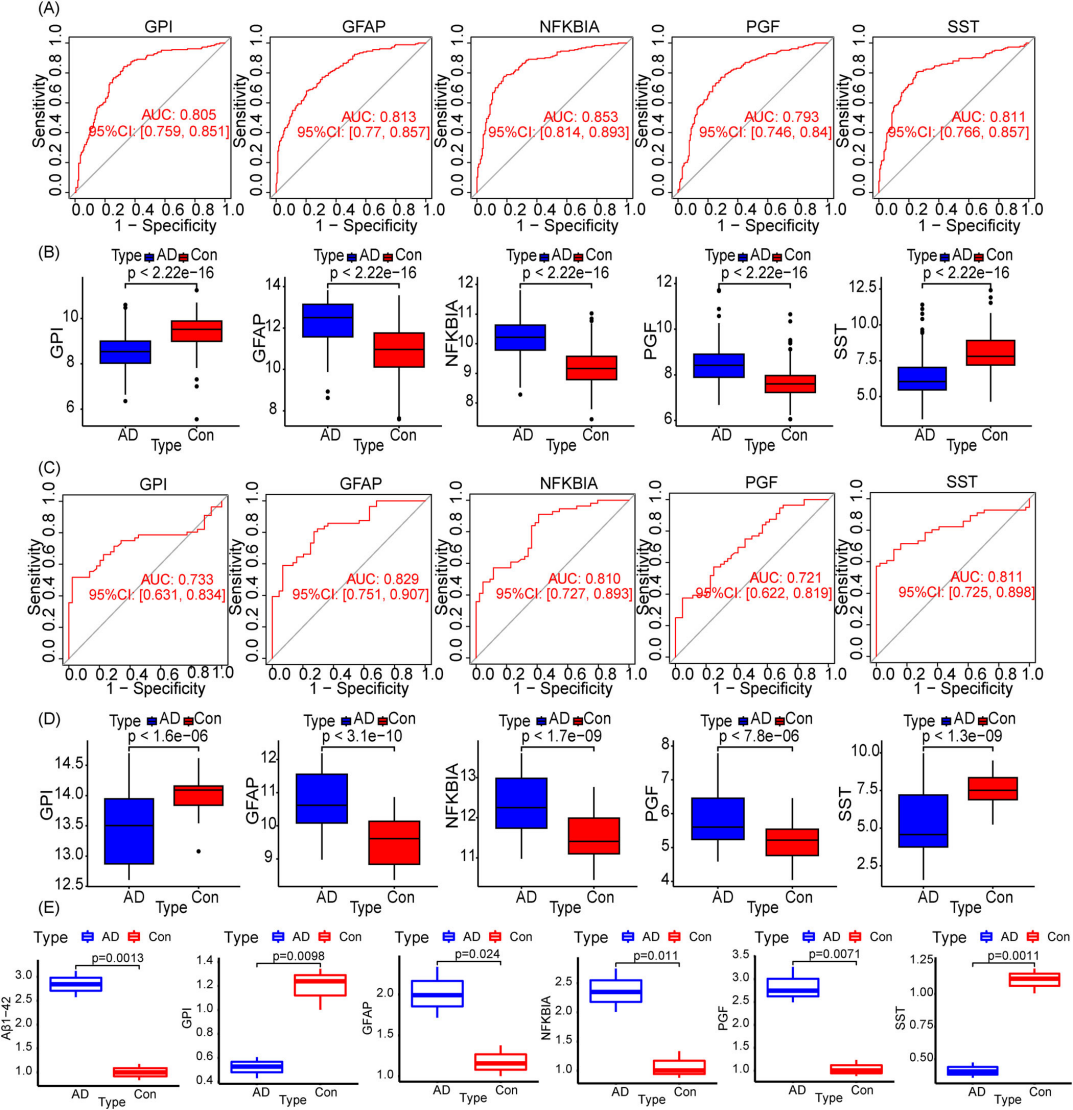
Evaluation and validation of diagnostic effects of characteristic diagnostic markers. (A) Receiver operating curve (ROC) of five markers. (B) Expression level of five markers. (C) ROC of five markers in GSE122063. (D) Expression level of five markers in GSE122063. (E) Relative mRNA expression levels of 5 hub genes using by RT‐PCR.

The genes that were identified were utilized to create the diagnostic model through the application of a logistic regression algorithm. The discrimination ability was additionally measured using AUC. The results from Figure 5A indicate that the biomarkers have a strong ability to differentiate between AD brains and control samples, with GPI having an AUC of 0.805 (95% CI = 0.759–0.851), GFAP having an AUC of 0.813 (95% CI = 0.770–0.857), NFKBIA having an AUC of 0.853 (95% CI = 0.814–0.893), PGF having an AUC of 0.793 (95% CI = 0.746–0.840), and SST having an AUC of 0.811 (95% CI = 0.766–0.857). These five genes show promise as reliable biomarkers for AD diagnosis. Additionally, it was noted that brain tissue samples from individuals with AD showed markedly elevated levels of GFAP, NFKBIA, and PGF in comparison to healthy tissue. Conversely, SST and GPI expression showed lower levels in AD patients (Figure 5B). To verify the accuracy and reliability of the above results, we used an external dataset (GSE122063) to verify the five hub genes. The findings indicated that the levels of GPI, GFAP, NFKBIA, PGF, and SST expression in AD patients were similar to those in the normal group as seen in the training set (Figure 5C). In addition, the area under the ROC curves of the four hub genes were all greater than 0.7, exhibiting a high diagnostic efficacy in distinguishing AD patients (Figure 5D). We also used Aβ1‐42 to model AD disease in PC12 cells and preliminatively verified the expression levels of five genes by fluorescent PCR. PCR results showed that the expression of Aβ1‐42 was significantly increased in the model group compared with the control group, indicating that the Aβ1‐42‐induced AD cell model was successful. We detected the expression trend of five hub genes consistent with the results of our training set and external datasets (Figure 5E). Both methods mentioned above validated the robustness of the machine learning model.

### 3.6 The Nomogram Model for Prediction of AD

**Figure 6:**
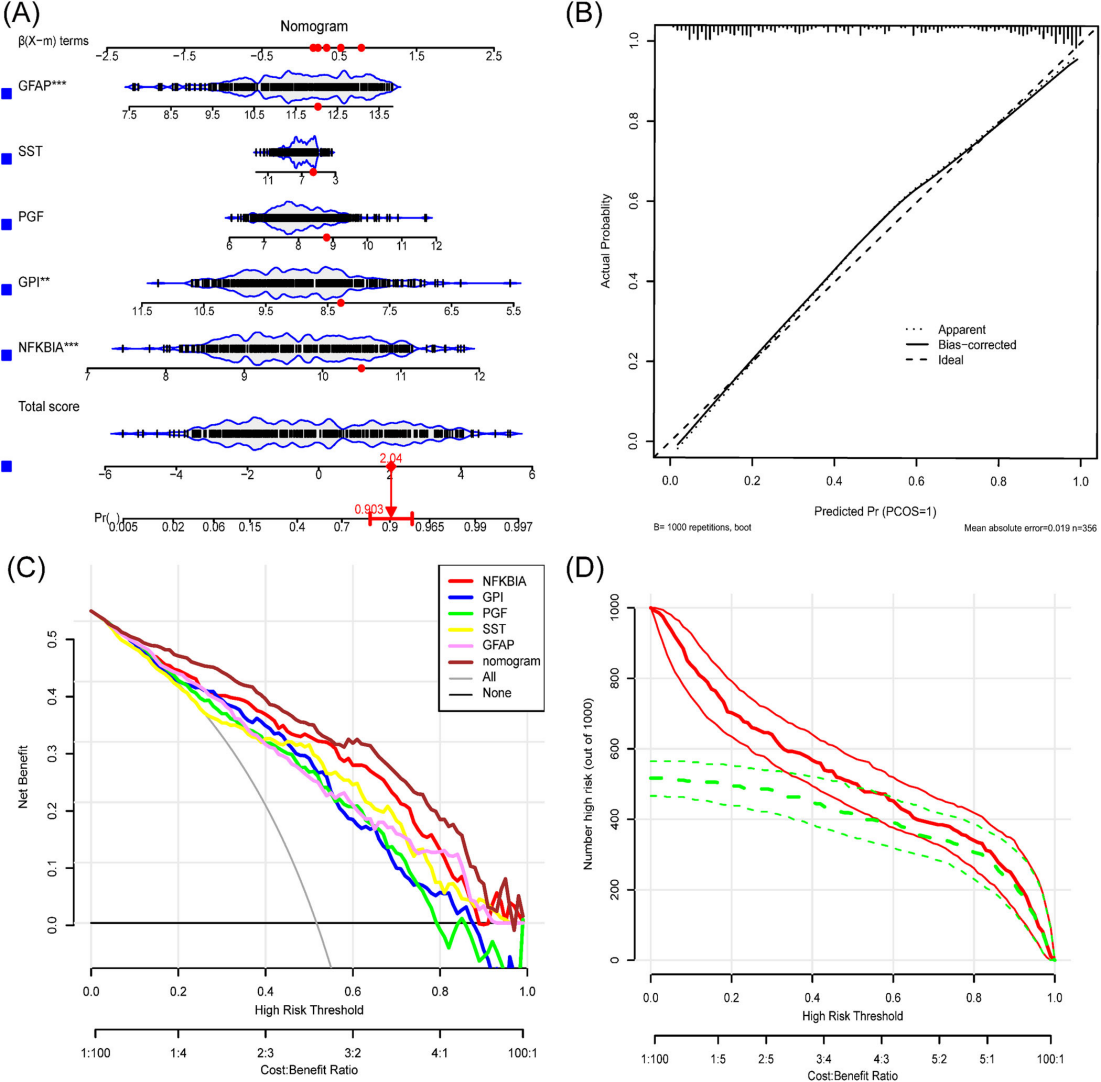
Construction and validation of nomogram models. (A) Nomogram model based on five markers. (B) Nomogram model correction curve. (C) Nomogram model clinical decision curve. (D) Nomogram model clinical impact curve.

We developed a nomogram model based on five pivotal genes to quantify risk assessment for AD patients. Figure 6 also includes the calibration curve, decision analysis curve, and clinical impact curve. Estimating patients' risk of AD can be achieved by adding up the scores of the model's features, offering a new approach for early detection and prevention of AD (Figure 6A). The nomogram model prediction reliability was shown by the calibration curves (Figure 6B). The red line consistently stayed higher than the gray and black lines on the DCA curve between 0 and 1, suggesting that decisions based on the model could offer extra advantages for patients with AD (Figure 6C). Moreover, the nomogram model demonstrated significant predictive ability as indicated by the clinical impact curve (Figure 6D).

### 3.7 Signaling Pathways Involved in Characteristic Genes

**Figure 7:**
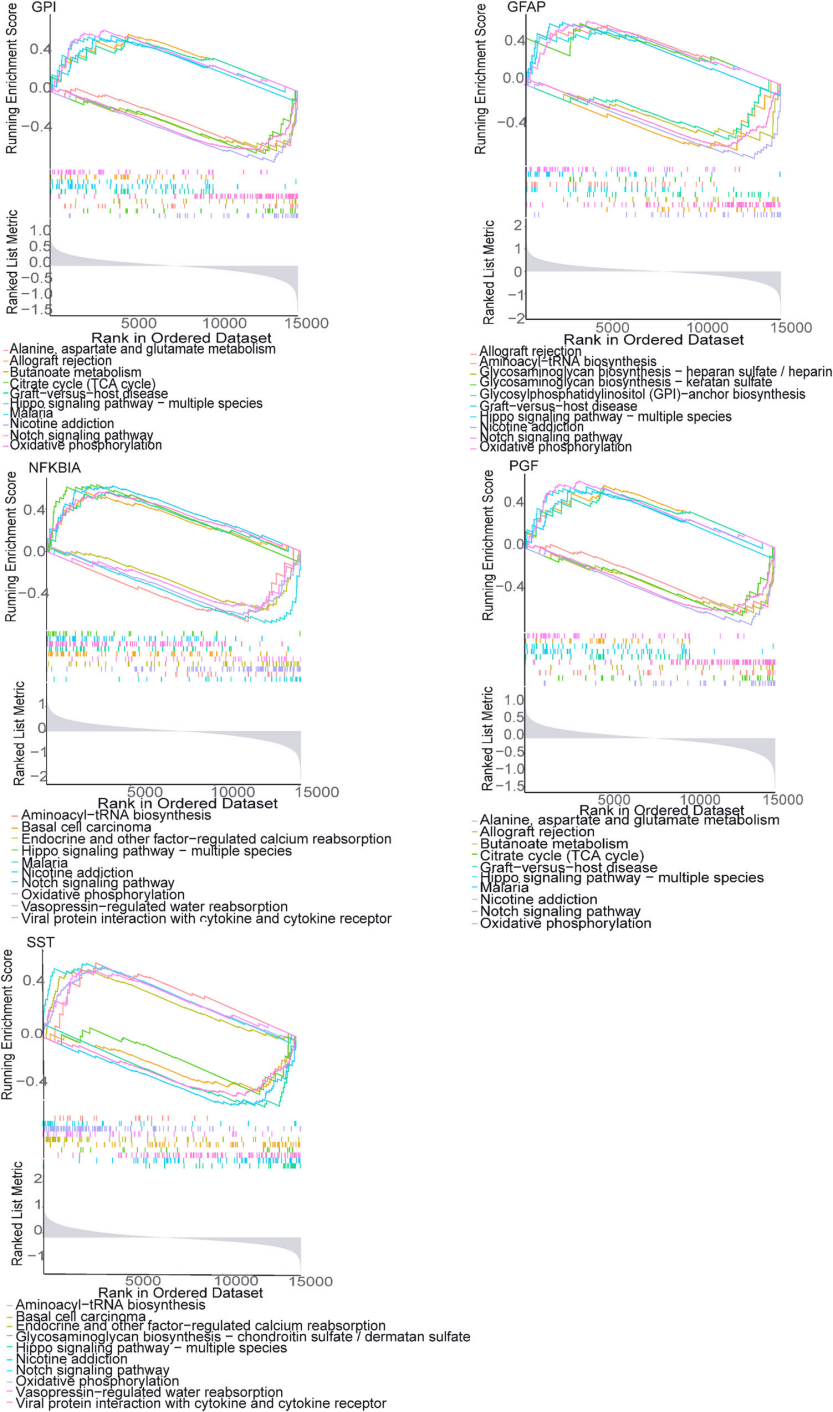
GSEA enrichment analysis results of five potential markers.

Despite the established clinical value of biomarkers demonstrated through machine learning and clinical predictive models, the distinct biological functions of each marker remain unclear. Further GSEA analysis revealed that these markers play a role in various pathways related to the onset and progression of AD, including Oxidative phosphorylation, Nicotine addiction, Hippo signaling pathway‐multiple species, Notch signaling pathway, Viral protein interaction with cytokine and cytokine receptor, Malaria, Allograft rejection, Graft‐versus‐host disease, Aminoacyl‐tRNA biosynthesis, Basal cell carcinoma, Endocrine‐regulated calcium reabsorption, vasopressin‐regulated water reabsorption, and several other pathways were identified. These genes showed significant correlations with Oxidative phosphorylation, Nicotine addiction, Hippo signaling pathway‐multiple species, and Notch signaling pathway, potentially indicating a connection to immune regulation in AD (Figure 7).

### 3.8 Analysis of Immune Infiltration and Immune Modulators

**Figure 8:**
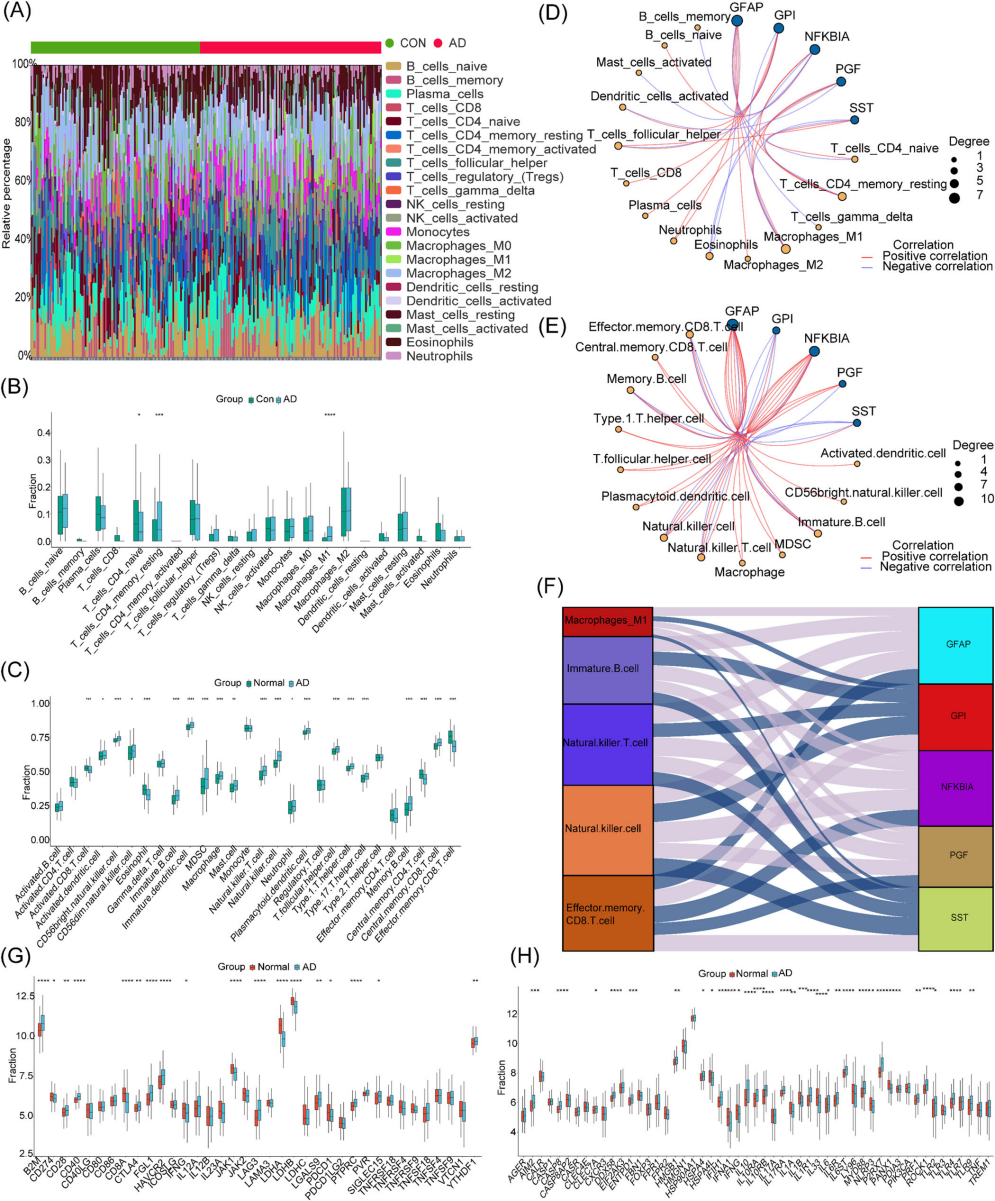
Analysis of immune infiltration and immune modulators. (A) Abundance of 22 immune cell types using Cibersort. (B) Abundance difference of 22 immune cell types based on Cibersort. (C) Abundance difference of 28 immune cell types based on ssGSEA. (D) Correlation analysis of 22 immune cell types and 5 biomarkers based on Cibersort: red represents positive correlation, blue represents negative correlation. (E) Correlation network of 28 immune cell types and 5 biomarkers based on ssGSEA, red represents positive correlation, and blue represents negative correlation. (F) Correlation analysis between 5 common immune cells and 5 biomarkers: purple represents positive correlation, blue represents negative correlation. (G) Analysis of 37 immune checkpoints in normal group VS AD group, red represents positive correlation. (H) Analysis of 53 ICDs in a normal group compared to the AD group, red represents a positive correlation. green represents negative correlation, *p < 0.05, **p < 0.01, ***p < 0.001.

Initially, we mapped the relative abundance of immune cells in AD compared to the normal population using the CIBERSORT algorithm (Figure 8A). Differential analysis of immune cells in AD patients and controls was further estimated based on Cibersort and ssGSEA in Figure 8B,C. Cibersort results showed that there was a high proportion of M1 macrophages and resting memory CD4T cells in AD patients, while the initial CD4T cells decreased. ssGSEA results showed that in AD patients’ tissues, the proportion of CD56bright natural killer cells, immature B cells and immature dendritic cells, myeloid suppressor cells, macrophages, mast cells, natural killer T cells, neutrophils, plasmacytoid dendritic cells, follicular helper T cells, helper T cells 1, helper T cells 17, memory B cells and central memory CD8 T cells were found increased. Conversely, a reduction in the proportion of activated CD8 T cells, eosinophils, central memory CD4 T cells, and effector memory CD8 T cells was observed. Based on the above two algorithms, we further analyzed the correlation between immune cells and hub genes, as shown in Figure 8D,E. By summarizing the results of the two algorithms for five genes, it was found that GFAP showed a positive correlation with CD56bright.natural.killer.cell, Memory.B.cell, Macrophage, MDSC, Central.memory.CD8.T.cell, and a negative correlation with Effector.memory.CD8.T.cell, Eosinophils, T_cells_CD4_naive, T_cells_gamma_delta. NFKBIA showed a positive correlation with Natural.killer.cell, Natural.killer.T.cell, Immature.B.cell, MDSC, Plasmacytoid.dendritic.cell, but a negative correlation with Effector.memory.CD8.T.cell, B_cells_memory, T_cells_follicular_helper, Eosinophils. PGF was positively correlated with Natural.killer.T.cell, Natural.killer.cell, Immature.B.cell, Macrophages_M1, T_cells_CD4_memory_resting, and negatively correlated with Effector.memory.CD8.T.cell, Eosinophils, Dendritic_cells_activated. GPI was positively correlated with T_cells_follicular_helper, T_cells_CD8, Plasma_cells, Dendritic_cells_activated, Effector.memory.CD8.T.cell, and negatively correlated with Natural.killer.cell, Immature.B.cell, Natural.killer.T.cell, Memory.B.cell, Macrophages_M1. SST was positively correlated with Effector.memory.CD8.T.cell, T_cells_CD4_naive, T_cells_follicular_helper, and negatively correlated with Natural.killer.cell, Natural.killer.T.cell, Immature.B.cell, Memory.B.cell, T_cells_CD4_memory_resting. Finally, we made a crossover analysis of the above results, and found that there is strong crosstalk between Effector.memory.CD8.T.cell, Immature.B.cell, Natural.killer.cell, Natural.killer.T.cell, T_cells_CD4_memory_resting, and Macrophages_M1 and these markers form a complex regulatory network, which may be an important part of the development of immune inflammation in AD (Figure 8F). Differential analysis of Immune checkpoints showed that 18 immune checkpoints were significantly different in AD group and normal group, including B2M, CD274, CD28, CD40, CD8A, and so on (Figure 8G). Difference analysis of ICDs showed that 31 ICDs significantly differed in AD and normal groups including AIM2, CASP8, DDX58, ENTPD1, IFIH1 and so on (Figure 8H). In addition, the gene sets related to immune checkpoints and ICD are presented in the Supporting Information. Therefore, the occurrence of AD may be related to the disorder of the immune microenvironment, the abnormal activation of immune inspection sites and immune death genes, and then cause inflammation and induce apoptosis.

### 3.9 Prediction of Potential Ameliorative Chinese Herbs and Compounds for AD

**Figure 9:**
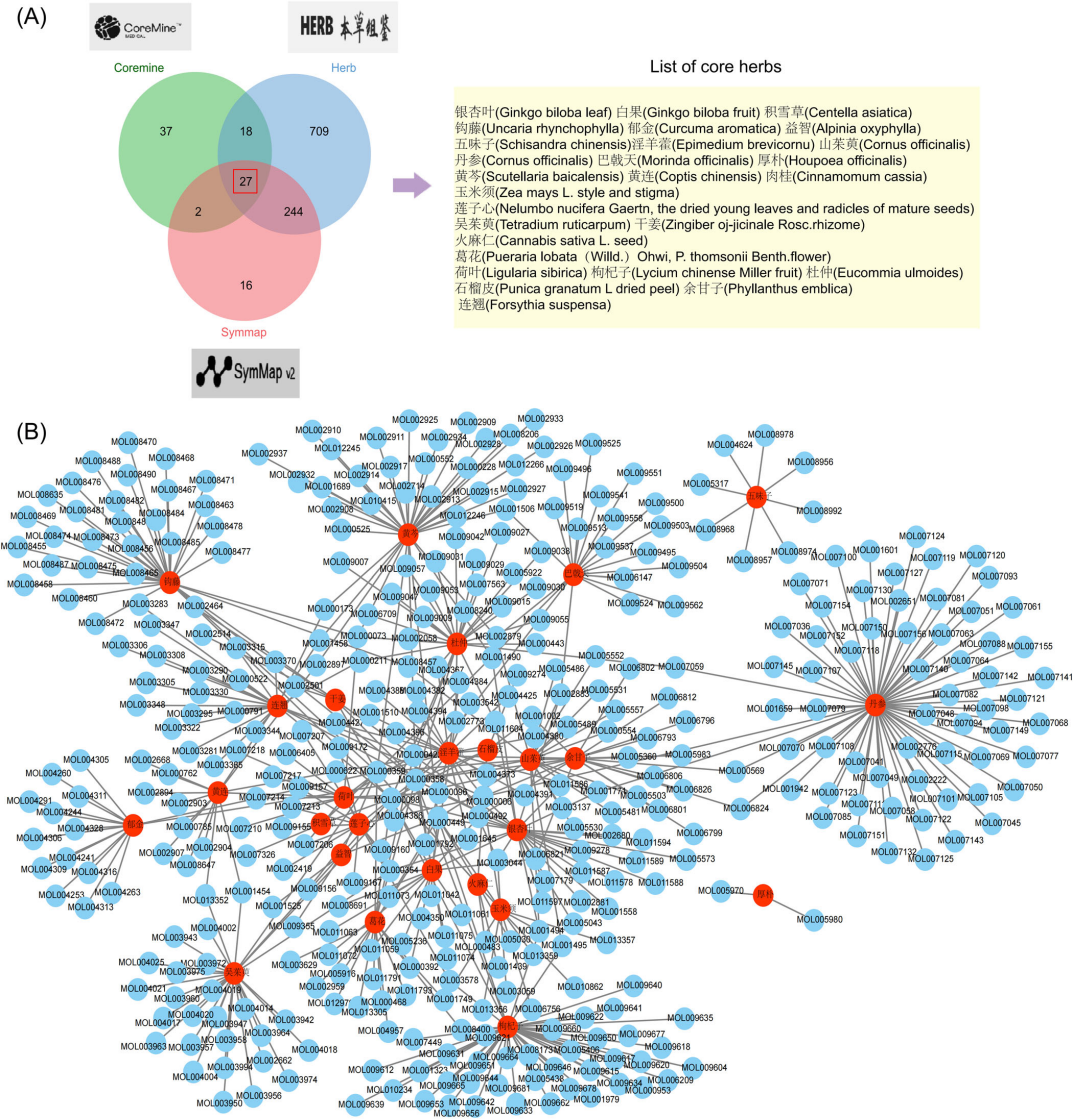
Prediction of potential ameliorative Chinese herbs and compounds for AD. (A) A Venn diagram and list of common herbs in three databases. (B) Potential core herbs‐compounds network.

We extensively searched three medical databases (Herb, Coremine, and Symmap) with AD as the keyword, and obtained 1053 potential TCMs (Supporting Information S1: File S7). After the intersection operation of the results, 27 flavors of TCMs were identified as the core TCMs for analysis. These TCMs were imported into the TCMSP database, and the components were screened out using OB > 30 and DL > 0.18 conditions. Subsequently, the findings above were utilized in the Cytoscape application to create network representing the relationship between targets, TCM, and components. According to the set parameters, seven core compounds, such as quercetin, were screened out for subsequent analysis (Figure 9A,B, Table 2).

**Table 2:** Topological parameters of key compounds.

Name	TCMSP_ID	Betweenness centrality	Closeness centrality	Degree
beta‐sitosterol	MOL000358	0.26011228	0.3961165	15
quercetin	MOL000098	0.20311502	0.37362637	15
luteolin	MOL000006	0.15325776	0.32025118	9
poriferast‐5‐en‐3beta‐ol	MOL001771	0.13476861	0.34056761	4
Stigmasterol	MOL000449	0.07286136	0.29955947	9
kaempferol	MOL000422	0.05648057	0.31578947	10
ent‐Epicatechin	MOL000073	0.04963222	0.32176656	4

### 3.10 Molecular Docking and MD Simulation

**Figure 10:**
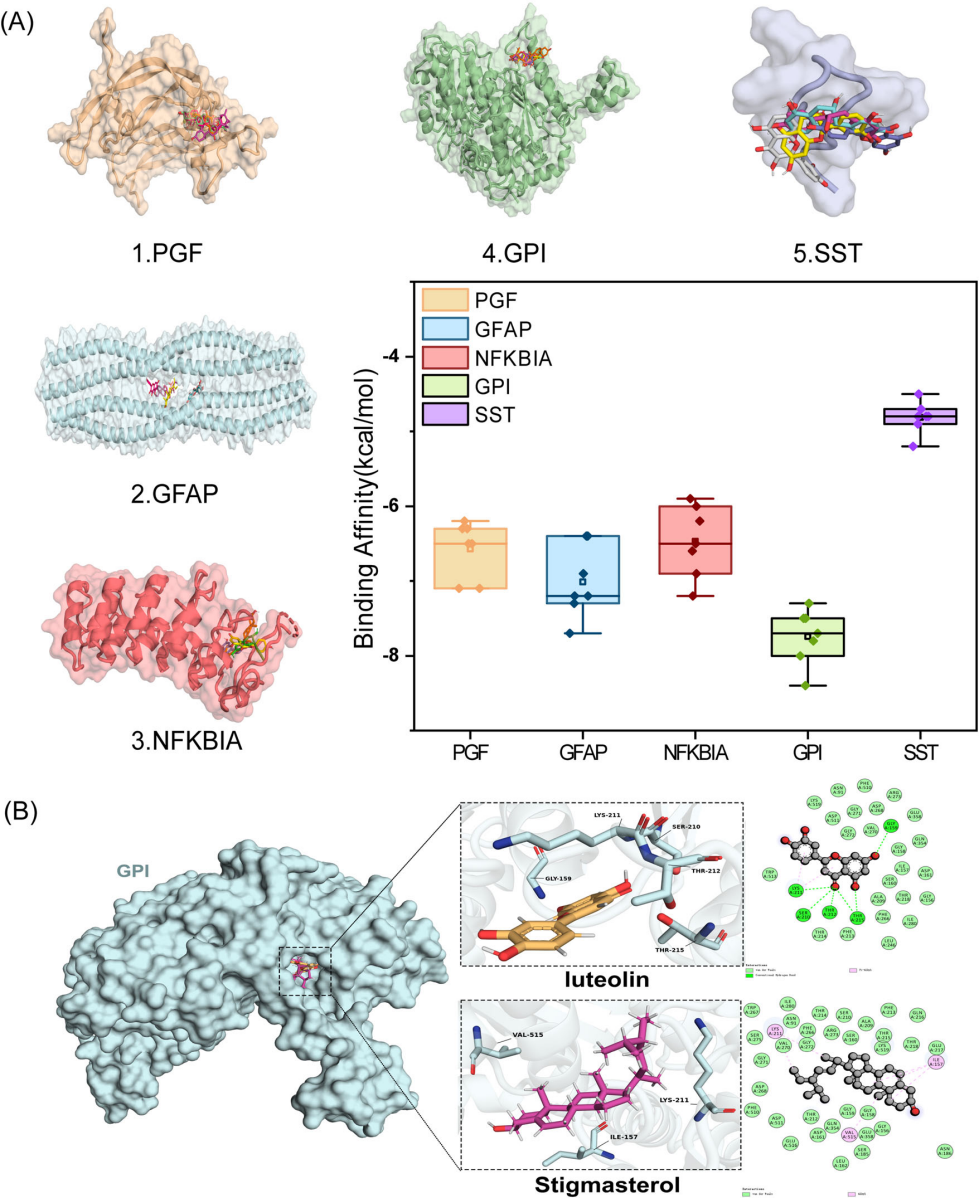
Molecular docking. (A) Batch docking and affinity results. (B) Autodocking result of Gpi‐luteolin, GPI‐stigmasterol.

**Figure 11:**
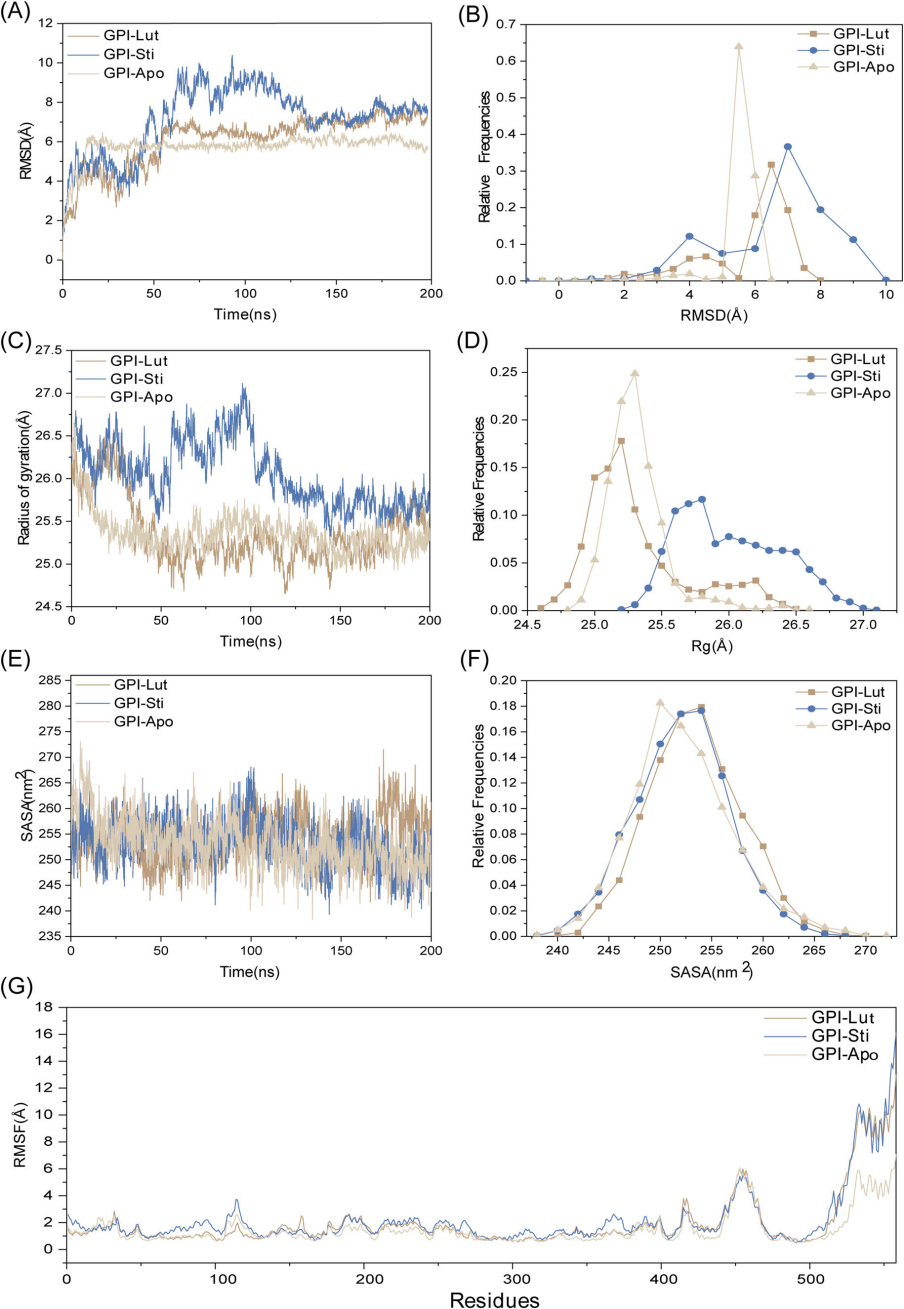
The molecular dynamics (MD) simulation of the GPI‐luteolin complex, GPI‐stigmasterol complex. (A) Temporal evolution of RMSDs from their initial structures for the three systems. (B) Relative frequencies of RMSDs. (C) Radius of gyration over the course of 200 ns MD for the three systems. (D) Relative frequencies of radius of gyration. (E) Solvent‐accessible surface area (SASA) over 200 ns MD. (F) Relative frequencies of SASA. (G) Root mean square fluctuations (RMSFs) of the C‐alpha (CA) atoms.

**Figure 12:**
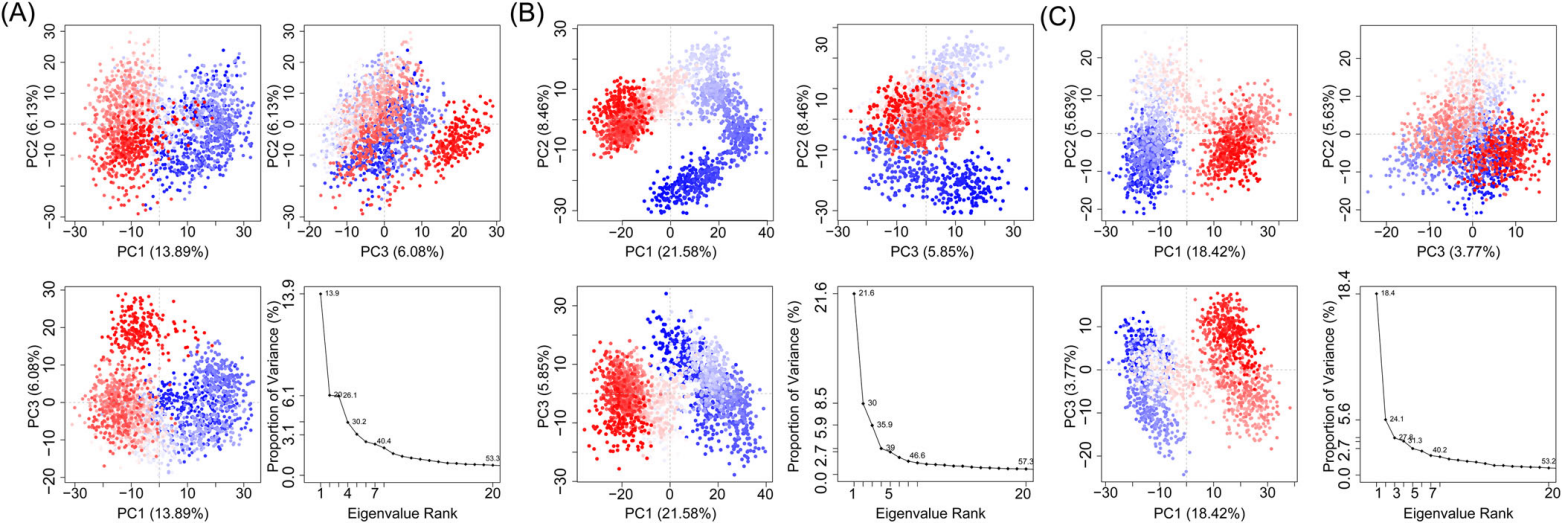
PCA of C‐alpha atoms in three systems: (A) GPI, (B) GPI‐Lut, (C) PSH‐Sti.

**Figure 13:**
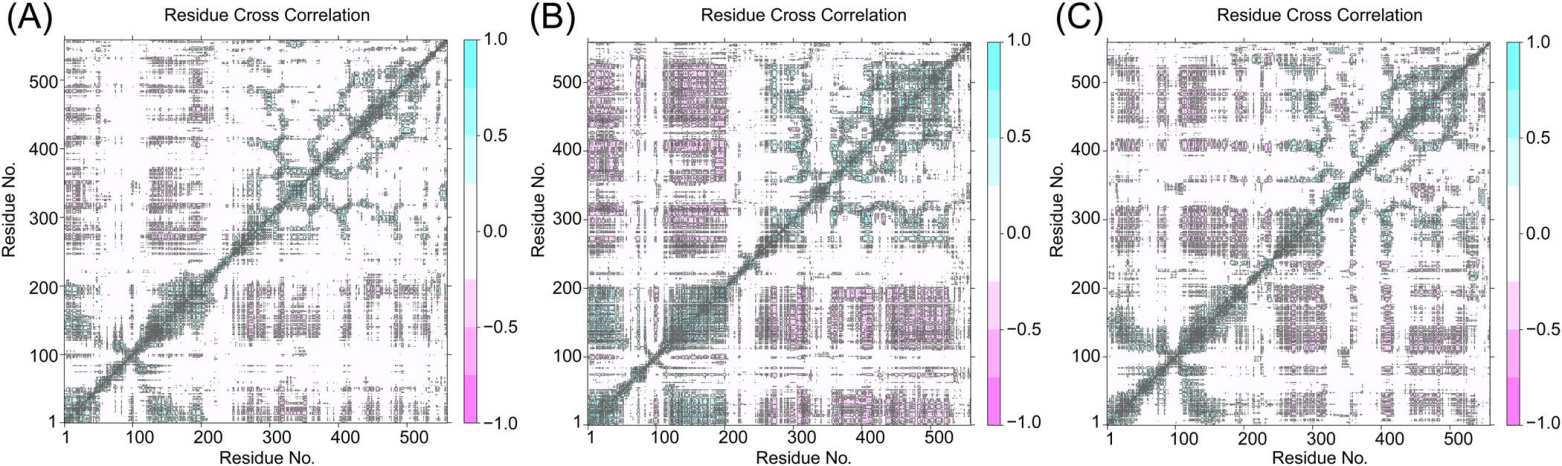
The interactive cross‐correlation chart for the 200 ns MD simulation paths of the three systems: (A) GPI, (B) Gpi‐luteolin, and (C) GPI‐stigmasterol. Cyan indicates positive regions, while pink indicates negative regions, showcasing correlated and anti‐correlated movements among residue CA atoms.

As shown in Figure 10A and Table 3, five proteins have stable binding with seven small molecules and strong affinity. In the GPI system, the dimensions of the GPI docking box were set to 43.05 Å × 40.95 Å × 64.05 Å, with the center coordinates at *x* = 1.369, *y* = 2.793, *z* = 0.373. Luteolin and stigmasterol docking with GPI are at the top of the list of binding affinity. The key residues of the two compounds binding with GPI are shown in Figure 10B. For the compound luteolin, residues Thr212, Lys211, Gly159, and Ser10 are crucial to the interaction. Stigmasterol is mainly bound to residues Val515, Ile157, and Lys211. Pi‐Pi and Pi‐alkyl interactions are crucial for the binding of all four ligands, with a significant contribution from tyrosine residues to the overall binding affinity. We furthered performed MD simulations of the two systems. The SASA of a protein can be used to analyze the hydrophobicity of the protein and the degree of closure of the protein surface. In Figure 11E, stigmasterol's SASA showed an overall decreasing trend after 125 ns, and most of it was lower than empty protein Apo, indicating that after binding with stigmasterol, the hydrophobicity of the protein increases, resulting in a more closed surface and stable lower than empty protein after 175 ns. The distribution center of stigmasterol's SASA is 255 nm^2^, while that of Apo is 250 nm^2^, which also follows the trend of the line graph (Figure 11F). The overall increase of luteolin after 100 ns was more significant than that of apo and stigmasterol; the hydrophobicity decreased, and the conformation may stretch after binding luteolin. The RMSD of the residue backbone's carbon atoms relative to their initial position is an indicator of the stability of the analysis simulation system, and it also shows the shift of the complex from the initial conformation. Analysis of RMSD showed that the three systems tended to be stable after 125 ns, while the RMSD of stigmasterol was higher than that of the empty protein (Apo) and luteolin after 50 ns, indicating more remarkerable conformational change (Figure 11A). Stigmasterol's distribution center is at 7 Å, which is also higher than that of Apo and luteolin, aligning with the above trend (Figure 11B). Rg reveals the tightness of the complex, and luteolin is smaller than stigmasterol, indicating that its binding conformation is more compact, but stigmasterol is slightly larger than Apo, as Apo is an empty protein without the support of ligands (Figure 11C,D). The RMSF of protein amino acids is used to analyze the extent of fluctuation of individual amino acids during the simulation process, revealing the flexibility changes of residues (Figure 12F). The RMSF of residues 100–120 and those after 500 show significant fluctuations when binding with molecules stigmasterol and luteolin. These fluctuations occur precisely at the binding sites of the small molecules, indicating high flexibility in these regions. The MM‐PBSA method was employed to estimate the binding free energy and assess the relative stability of the model. The results showed that the binding free energy of luteolin was −18.47 ± 2.58 kcal/mol, indicating that its binding affinity was lower than that of stigmasterol, which had a binding free energy of −20.48 ± 1.25 kcal/mol. This suggests that the binding between stigmasterol and the target enzyme is more robust and may have more significant potential in related biological functions or drug design (Table 4). Figure 12 displays the protein conformational change projections using PCA for three systems: Apo, GPI‐lut, and GPI‐sti. Figure 12A shows that the projection of the empty protein on principal component 1 (PC1, 13.8%) and principal component 2 (PC2, 9.6%) indicates that the protein has an extensive range of motion on these two principal components. In Figure 12B, the projection of proteins bound to luteolin on PC1 (21.6%) and PC2 (10.8%) showed significant conformational changes, indicating that large conformational changes occurred after small molecule binding. In Figure 12C, the projection of proteins bound to stigmasterol on PC1 (18.4%) and PC2 (9.7%) shows significant conformational changes. The eigenvalue plots show that PC1 and PC2 explain most conformational changes in all systems. The covariance matrix for each residue in the three systems was calculated to explore the changes in protein internal motional patterns. Figure 13 shows the three systems’ cross‐correlation matrix of Apo, GPI‐lut, and GPI‐sti protein residues. The negative correlation between 200 and 300 residues in Figure 13A suggests that these residues move in opposite directions without small molecule binding, potentially aiding the protein's structural stability when unbound. Figure 13B shows that after the binding of luteolin, the negative correlation between residues 100–200 and 300–400 is enhanced relative to empty protein. Figure 13C shows that after the binding of stigmasterol, the negative correlation between residues 200–300 and 400–500 is more robust. For the above details, see Supporting Information S1: Material S8. These findings indicating a significant counter‐movement in domain interactions, possibly due to interactions between domains caused by small molecules.

**Table 3:** The results of molecular docking simulation (Kcal/mol).

Compound	PGF	GFAP	NFKBIA	GPI	SST
Ent‐epicatechin	−6.3	−7.3	−6.5	−7.3	−4.8
Beta‐sitosterol	−6.3	−6.9	−5.9	−7.8	−4.9
Poriferast‐5‐en‐3beta‐ol	−6.2	−6.4	−6.2	−7.7	−4.8
Quercetin	−7.1	−7.2	−6.6	−7.5	−4.7
Luteolin	−7.1	−7.7	−6	−8	−4.8
Stigmasterol	−6.5	−6.4	−7.2	−8.4	−5.2
Kaempferol	−6.5	−7.2	−6.9	−7.5	−4.5

**Table 4:** The results of MM‐PBSA(Kcal/mol).

GPI‐system	Luteolin	Stigmasterol
ΔEvdW	‐25.13 ± 1.05	−43.28 ± 1.09
ΔEele	−40.98 ± 4.69	−3.02 ± 1.69
ΔGgas	−66.11 ± 3.89	−46.30 ± 1.95
ΔGsolv	47.64 ± 2.93	25.83 ± 1.80
ΔGtotal	−18.47 ± 2.58	−20.48 ± 1.25

## 4 Discussion

Despite extensive research, the exact pathogenesis of AD remains elusive. Recent studies have highlighted the significant involvement of immune mechanisms in the development and progression of AD. Research indicates that immune system dynamics play a critical role in advancing AD. Specifically, immune cell infiltration in the brain may regulate the clearance of amyloid beta plaques and potentially slow the progression of the disease [48, 49, 50]. Moreover, the unusual accumulation of amyloid beta in the brain can attach to specific immune cell types like CD36, TLR4, and TLR6, leading to the generation of inflammatory proteins and signaling molecules [51, 52]. Consequently, this has triggered inflammation within the brain, exacerbating neuronal harm in individuals with AD. However, there is a paucity of systematic research protocols on the key biomarkers, molecular regulatory mechanisms, and potential target herbs and compounds in AD immunity. Exploring these deficiencies could open up new opportunities for creating successful treatment options. We employed an integrated approach combining bioinformatics, machine learning, molecular docking, and MD simulation to comprehensively analyze essential genes, potential herbs, and compounds involved in the immune mechanisms of AD.

Five immune‐related markers (PGF, GFAP, SST, GPI, NFKBIA) were discovered in the study, demonstrating reliable performance in both the training and validation datasets, and suggesting a solid diagnostic potential for AD. PGF, a polypeptide hormone belonging to the VEGF family, has diverse physiological effects and is implicated in immune response, vascular balance, and angiogenesis. It is crucial in cell metabolism and linked to conditions like atherosclerosis, diabetic neuropathy, myocardial infarction, epilepsy, and other diseases [53, 54]. A recent study conducted by MGH discovered that elevated levels of plasma PGF can speed up the development of early neocortex tau pathology and cognitive decline in individuals with preclinical AD, which aligns with our conclusions from gene‐level research [55].

Ehtewish et al. [56] identified eight blood proteins through blood proteomics that effectively distinguish AD from healthy individuals. Among these proteins, placental growth factor (PGF) exhibits a significant negative correlation with cognitive ability and is associated with biological pathways involved in immune responses and vascular injury [56]. These findings align with our research results and further affirm the potential of PGF as a valuable clinical target. The autopsy study found that higher prefrontal cortex expression of VEGFB, FLT4, FLT1, and PGF was associated with worse cognitive trajectories, β‐amyloid burden, tau burden. VEGF ligand and receptor genes, specifically genes relevant to FLT4 and FLT1 receptor signaling, are associated with cognition, longitudinal cognitive decline, and AD neuropathology [57]. In addition, Wu et al. [58] showed that PGF is a strong indicator with a notable link to higher levels of CeVD in AD within a group of 109 Asian AD patients. The association between PGF and AD has primarily been established through clinical cohort studies, yet the precise molecular mechanism remains to be fully elucidated. Our research initially validated that PGF can trigger multiple immune cell pathways in the brain affected by AD, leading to disruptions in biological functions. Additionally, we identified several compounds through screening, laying the groundwork for further investigation into the molecular mechanisms of PGF. GFAP, a structural protein primarily present in astrocytes of the brain, is a crucial indicator of glial cell activation and brain damage or illness [59]. Research has demonstrated that elevated levels of GFAP in the bloodstream of patients are associated with more severe pathological Aβprotein development, serving as a potential indicator for diagnosing AD in clinical settings [60, 61, 62]. A meta‐analysis of 31 clinical studies found that the blood GFAP level was significantly elevated in the Aβ positive group compared to the opposing group, indicating its potential use in the early detection of AD [63]. The above clinical studies are consistent with our results, demonstrating that glial fibrillary acidic protein (GFAP) is an excellent marker for diagnosing AD. Chatterjee et al. [64] found that elevated levels of GFAP in patients from families with autosomal dominant Alzheimer's disease (ADAD) emerged 10 years before the expected onset of symptoms. These elevated GFAP levels were associated with amyloid positivity in asymptomatic ADAD individuals and increased in conjunction with the severity of clinical symptoms. A recent high‐resolution 7T MRI study demonstrated that p‐tau181 and GFAP robustly reflect changes detected by 7T MRI in AD. The strongest association was found between frontal/parietal MRI changes and MRS neuroinflammation [65]. Our study, grounded in an immunological perspective, confirmed the strong association between GFAP and AD, suggesting that GFAP serves as a robust, reliable, and valuable diagnostic biomarker. SST, a suppressive hormone related to sexuality, is found throughout the human brain and body. Song et al. [66] explained in their study the unique role of SST in the cerebral cortex, which can enhance the visual discrimination and directional selectivity of V1 neurons in mice. Han et al. [67] found that when encountering copper, Aβ, and metal‐Aβ complexes, somatostatin accumulates a lot to reduce the toxicity and aggregation of metal‐Aβ complexes, thereby reducing toxic damage to the brain. Furthermore, the anxiety‐like behavior associated with E/I imbalance in hippocampal synapses of 5xFAD mice results from the loss of SST+ and PV+ interneurons in the ventral hippocampus (vHPC). This finding provides a better explanation for the psychiatric symptoms, including anxiety and depression, observed in patients with early AD [68]. Rofo et al. [69] demonstrated that three intravenous injections of SST‐scFv8D3, a brain‐penetrating SST peptide, resulted in upregulation of mitochondrial proteins and synaptic proteins involved in membrane transport and neuronal development in the hippocampus of APPswe mice compared to wild‐type mice. Therefore, as an important pathological marker of AD, supplementing or regulating SST to increase brain levels may reduce Aβ damage and improve cognitive function in patients. The protein encoded by GPI is an critical enzyme in the process of glycolytic phosphate isomerization, which is involved in the process of sugar metabolism and sugar synthesis. Currently, it has been demonstrated that hexokinase (HK), phosphofructokinase‐1 (PFK‐1), and pyruvate kinase (PK) are crucial targets in the abnormal glucose metabolism of AD, showing promise as markers for AD pathology [70, 71]. There is limited research on GPI within the realm of AD. However, understanding the development of autoimmune conditions such as rheumatoid arthritis and systemic lupus erythematosus is an area of significant interest. Additionally, GPI serves as a prevalent immune indicator for diagnosing these diseases clinically [72]. Outside the cell, GPI can act as an important nutrient factor for spinal nerves and promote the normal development of neurons [73]. Previous animal experiments have demonstrated that increased expression of the GPI gene and its receptors may enhance synaptic mechanisms involved in learning and memory formation in vivo [74]. Hence, we are confident that investigating the scientific research potential of GPI in the immune mechanism of AD and drug development is necessary. NFKBIA, a transcription factor, interacts with NF‐κB to inhibit its nuclear entry, impacting immune response, apoptosis, cell proliferation, and influencing the advancement of AD. Recent studies have found that NFKBIA is highly expressed in patients with AD and is closely associated with hypoxic microenvironments and copper metabolism immunity, highlighting its significance as an important biomarker for the diagnosis and treatment of AD [75, 76, 77, 78]. Ryu et al. [79] demonstrated that increased levels of NFKBIA in the brain can suppress the activation of the NF‐κB signaling pathway, resulting in a decline in cognitive function, dementia, and AD. In addition, NFKBIA is regulated by SIRT1 and is closely related to aging diseases [80]. In AD, multiple pathological pathways interact, and inhibiting NFKBIA expression may improve cerebral hypoxia, reduce inflammation, and alter metabolic pathways, warranting further investigation as a therapeutic strategy.

Enrichment analysis findings uncovered crucial biological processes and signaling pathways that could be significant in the development of AD, enhancing comprehension of the immune mechanisms involved in AD. GSEA results showed that the glycosaminoglycan biosynthesis pathway, Hippo classical signaling pathway, nicotine addiction, and other pathways are closely related to multiple biomarkers. Glycosaminoglycans generated through the glycosaminoglycan biosynthesis route not only hinder the activity of α‐secretase in cleaving APP but also enhance the metabolic pathway of APP involving the β‐secretase enzyme, leading to elevated levels of Aβ [81]. The Hippo signaling pathway, which is highly conserved in mammals, plays a critical role in regulating tissue and organ size, as well as controlling cell proliferation, differentiation, and apoptosis [82]. Wang et al. [83] found that the regulation of MST1 in the Hippo pathway can directly and directly resist and improve the pathological features and clinical symptoms of AD without relying on the clearance of Aβ. Our results found that the four markers are related to the Hippo pathway, so the regulatory relationship between these markers and the MST1 gene can be further explored in the future, and efficient and effective clinical drugs can be explored. Nicotine addiction pathways are important in the brain for synaptic transmission, learning and memory, and addictive behavior. New research indicates that the irregular Ly6/uPAR protein expression disrupts the α7‐nAChR function in the cholinergic system in the initial phase of Aβbuild in AD. This leads to increased neuroinflammation and degeneration of cerebellar astrocytes [84]. A new medication called NBP, a cyclic form of T14, was created that inhibits the binding of its linear form to the alpha‐7 nicotinic receptor in the brain of Alzheimer's patients in a dose‐dependent manner. Administering NBP14 intranasally for 14 weeks resulted in decreased amyloid levels in the midbrain and improved normal object recognition in 5XFAD mice [85].

According to Cibersort and ssGSEA results and correlation analysis, M1 macrophages, resting memory CD4T cells, memory B cells, and immature dendritic cells were found in AD patients. Active CD8 T cells, eosinophils, central memory CD4 T cells, effector memory CD8 T cells, and other immune types are closely related to the biological process of AD. We reviewed the progress of previous immune cell studies [86, 87] and found that the above incorrect distribution of immune cells can lead to neuronal degeneration and further deterioration of AD. For example, studies have shown that Aβ protein can also activate macrophages to produce many inflammatory cytokines and reactive oxygen species, which aggravate cell damage [88, 89]. The CD4+ subtype Th1 and Th17 affecting T cells in this study have been confirmed to down‐regulate the role of Treg cells in the peripheral nervous system and central nervous system, promote the activation of glial cells, and the enhancement of neuroinflammation, lead to pathological changes in AD and aggravate the memory impairment of patients [90]. In addition, it was found that compared with the healthy group, the proportion of initial B cells in AD patients decreased, memory B cells increased, and the high level of TNF‐α released by activated B cells promoted the formation of plaques in AD brain [91], which was more consistent with our results of immune infiltration. A literature review found that recent studies on AD immune factors mainly focus on T cells, B cells, astrocytes, inflammatory factors, inflammasome [90, 91, 92, 93]. At the same time, we also found significant differences in 18 immunodetection sites, such as B2M, JAK1, and LDHB. These immune examination sites participate in the immune escape of tumors, and are closely related to the occurrence and development of cancer. Zhao et al. [94] revealed a class of amyloid copolymers, B2M‐Aβ, identified B2M as a critical factor mediating amyloid toxicity. Based on B2M's ability to cross the blood‐brain barrier, they proposed targeting peripheral B2M as a therapeutic strategy for AD. JAK1 is an essential member of the JAK/STAT signaling pathway, which can contribute to the progression of neurodegenerative diseases such as AD by activating innate immunity, coordinating adaptive immune mechanisms, and promoting neuroinflammation [95]. These studies corroborate our findings and bring new directions for future AD treatment. Difference analysis of ICDs showed 31 ICDs were significantly different in AD and normal groups. The analysis of ICD genes revealed 31 genes with significantly altered expression levels when comparing the AD cohort to the healthy control group. ICD can trigger an immune response and is an essential process under various pathological conditions [96]. The damage‐associated molecular patterns (DAMPs) released during ICD, such as HSPs and HMGB1 proteins, are involved in the immunoinflammatory response in neurodegenerative diseases such as Alzheimer's [97]. In addition, the activation of P2RX7, NLRP3, and other genes is associated with the release of pro‐inflammatory cytokines, induction of neuronal deformation, and induction of the deterioration of Aβ plaque spread, and tau protein abnormalities [98, 99, 100]. The findings of these current studies confirm the results of our differential analysis, and we have also identified multiple under‐reported immunomodulators in the field of AD. We believe that these interactions occurring in the immune microenvironments, as a rich biological resource, provide more possibilities and flexibility for researchers to explore the mechanisms and drug targets of AD and are worthy of further exploration and verification in the future using single‐cell sequencing, spatial omics, and other technologies.

Currently, it is widely accepted that Aβ and Tau proteins are the primary causes of AD in clinical practice. However, during the asymptomatic progression of AD patients over several years, various biological reactions and regulatory mechanisms, including oxidative stress, changes in mitochondrial function, and neuroinflammatory factors, also play a significant role in the disease progression [101]. The modern approach to drug research focuses on single targets, which has led to a bottleneck in AD drug development over the past few decades [102]. In China, TCM has a rich history in disease treatment, and its characteristics of multiple components, targets, and pathways align well with the complex network regulatory mechanism of AD. Therefore, screening important compounds from TCM has become a promising avenue for AD drug development. Molecular docking is a valuable tool in drug design as it can predict the conformation of small molecules and targets, and it has been widely used in numerous studies for screening potential drugs [103]. MD is performed under controlled simulations and appropriate force fields, utilizing mighty computing power to test the feasibility of stable combinations in natural environments [104]. Thus, we utilized molecular docking and MD techniques to screen and validate core compounds to enhance the efficiency of our drug screening process. In this study, we searched the herbal medicine database using the keyword “Alzheimer's disease” and identified 27 Chinese medicines that may have therapeutic effects. Subsequently, we utilized the TCMSP database to identify the corresponding compounds and constructed the herb‐compound network. Among the identified compounds, seven candidate compounds, including quercetin, exhibited promising interactions and stability. We selected the three combinations with the lowest binding energies from the results above, specifically Gpi‐luteolin and GPI‐stigmasterol for further analysis using kinetic simulation. The stable binding of the two compounds to their respective proteins and their strong effects provide robust evidence to support the further development of drugs related to AD immunotherapy.

The recent study reveals the efficacy of luteolin in treating various neurological diseases [105]. For instance, luteolin has shown significant improvement in spatial learning and memory impairment in rats induced by streptozotocin (STZ), along with the elimination of STZ's inhibitory effect on the thickness of the pyramidal cell layer in the CA1 area of the hippocampus [106]. Choi et al. [107] discovered that luteolin and its glycoside derivatives effectively inhibit the activities of AChE, BChE, and BACE1, thereby exhibiting anti‐AD activity. Stigasterol can improve the inflammatory response of AD cells induced by Aβ42 by activating AMPK to mediate the NFκB and NLRP3 signaling pathways [108] and inhibit oxidative stress‐induced cell death by stimulating the expression of deacetylase (SIRT1) [109]. A recent Mendelian randomization study on phytosterols and AD showed a potentially beneficial effect of stigmasterol and sitosterol in the blood in reducing the risk of AD [110], which also indirectly demonstrated the indirect value of stigmasterol in clinical drug and functional food development. Many current literature reports [111, 112, 113, 114]have shown the improvement effect of two compounds on the inflammation level of AD, such as TNF‐α, IL‐1β, and IL‐6. However, these studies only observed at the concentration level. The potential of treating AD through anti‐inflammatory pathways has been illustrated from various perspectives [115]. However, relatively little has been reported on the interaction of compounds with these critical factors in immune inflammation.

Natural compounds are typically derived from herbal plants, which possess considerable medicinal value and historical usage experience, making them significant resources for new drug development. As of 2023, more than 2865 clinical trials have been conducted for the treatment of AD, with only 13 compounds currently under investigation or reporting results following a rigorous screening process. These drugs include d‐mannitol, quercetin, isorhamnetin, and ginkgolide, the majority of which are administered via single injections or oral intake [116]. The research and development of natural drugs for AD primarily emphasize computer‐based virtual screening alongside in vitro and in vivo experimental validation. Many natural compounds, including crocin, astaxanthin, and carotenes, exhibit significant antioxidant and anti‐inflammatory properties; however, their low solubility and poor stability considerably limit their clinical applications [117]. For instance, Dhas and Mehta have developed spherical nanoparticles using electrostatic interactions and nanoprecipitation techniques, featuring a chitosan shell and a PLGA core, with particle sizes under 150 nm [118]. When administered through the nasal cavity, these nanoparticles can more rapidly reach the brain, mitigating the effects of environmental factors such as temperature, light, and oxygen on molecular stability. Experimental findings demonstrate that these nanoparticles penetrate the blood‐brain barrier more effectively than pure lutein suspensions and PLGA nanoparticles while exhibiting high reactive oxygen species scavenging activity, thereby achieving therapeutic effects against AD. Compared to small molecules, natural polyphenol nanoparticles exhibit stronger anti‐amyloidogenic properties and can also penetrate the blood‐brain barrier, offering an attractive model for the development of AD therapies [119]. Currently, studies on compounds such as luteolin primarily focus on verifying their pharmacological mechanisms, with relatively few clinical trials conducted. We screened potential compounds for target markers based on molecular docking technology and simulated the microscopic evolution of the binding states of GPI‐Luteolin and GPI‐Stigmasterol through MD, providing scientific basis for the development of new drugs and the design of administration routes based on three compounds. we speculate that these two compounds have strong pharmacodynamic effects, are promising for improving AD by regulating immunity, providing some insights for the treatment and drug development of AD.

Future research should conduct both in vivo and in vitro experiments on the core biomarkers identified in this study to validate the specific regulatory mechanisms. Simultaneously, clinical practitioners may combine compounds with similar effects or integrate them into mainstream therapeutic approaches to innovate treatment strategies and conduct clinical trials. Studies on natural compounds for AD should emphasize their intrinsic properties, including multi‐targets and multi‐pathways. Mechanistic investigations must incorporate contemporary biological techniques, such as multi‐omics and single‐cell sequencing, to identify common targets across various biological pathways associated with immunity, oxidative stress, autophagy, and mitochondrial function in AD. This strategy can effectively optimize the pharmacological effects of natural compounds. Additionally, certain physicochemical properties of these compounds pose significant challenges in drug development. It is essential for researchers in medicine, physical chemistry, materials science, and pharmacology to collaborate and focus on aspects such as molecular structure, innovative materials, and drug delivery systems to enhance bioavailability and improve anti‐AD efficacy. However, some limitations need to be highlighted. This study provides a preliminary analysis of AD‐related immune markers, utilizing chips obtained from various brain regions, which may introduce certain biases. Future screening efforts should differentiate among these brain regions to identify more precise markers that accurately represent their functional characteristics. The machine learning component of this study employed a limited number of screening methods and validation datasets. In subsequent research, we will expand both the variety of machine learning methods and the number of validation datasets to enhance the stability of these biomarkers. Furthermore, the selection of Chinese herbs and core compounds is based on existing database information and topological parameters. In the future, we plan to establish a natural compound organoid screening platform to efficiently and rapidly identify potential anti‐AD drugs. Ultimately, this study was conducted using bioinformatics analysis, and further experimental research is necessary to validate the regulatory relationships between core targets and compounds.

## 5 Conclusion

We have identified five immune‐related markers, PGF, GFAP, SST, GPI, and NFKBIA that can be utilized for early diagnosis and pathological mechanism research of AD. These markers are associated with various immune cell types, modulators, and related biological functions and involve multiple pathways such as the Hippo pathway and glycosaminoglycan synthesis pathway. These findings further underscore the role of immune regulatory mechanisms in the progression of AD. Finally, we provide seven promising natural compounds such as luteolin and stigmasterol that target these biomarkers. Our study offers fresh perspectives on utilizing immunotherapy for treating AD and creating medications and nutritious products derived from plants.

## Author Contributions

**Pengpeng Liang** and **Hongyan Wu:** conceptualization. **Pengpeng Liang** and **Yale Wang:** methodology. **Jiamin Liu** and **Hai Huang:** software. **Jinhua Kang** and **Yue Li:** validation. **Pengpeng Liang:** formal analysis. **Yale Wang** and **Jiamin Liu:** investigation. **Hai Huang** and **Yue Li:** resources. **Jinhua Kang:** data curation. **Pengpeng Liang** and **Yale Wang:** visualization, writing — original draft preparation. **Pengpeng Liang** and **Jiamin Liu:** writing — review and editing. **Guiyun Li:** supervision. **Guiyun Li** and **Hongyan Wu:** project administration. **Hongyan Wu:** funding acquisition. All authors have read and agreed to the published version of the manuscript.

## Ethics Statement

The authors have nothing to report.

## Consent

The authors have nothing to report.

## Conflicts of Interest

The authors declare no conflicts of interest.

## Supporting information

Supporting information.

## Data Availability

The original contributions presented in this study are detailed in the article and Supporting Material. Further inquiries can be directed to the corresponding authors. The datasets utilized and analyzed during the current study are accessible from GEO (http://www.ncbi.nlm.nih.gov/geo).
