# Supplementary material for: Identification and Exploration of Immunity‐Related Genes and Natural Products for Alzheimer's Disease Based on Bioinformatics, Molecular Docking, and Molecular Dynamics
Source: Immun Inflamm Dis. 2025 Apr 7;13(4):e70166. doi: 10.1002/iid3.70166 (PMC11973734; doi:10.1002/iid3.70166)
Supplement: Supplementary file 1 — Supporting information. [file IID3-13-e70166-s001.zip › Supplementary materials/S7-herb and ingredients/27 core herb.pdf]

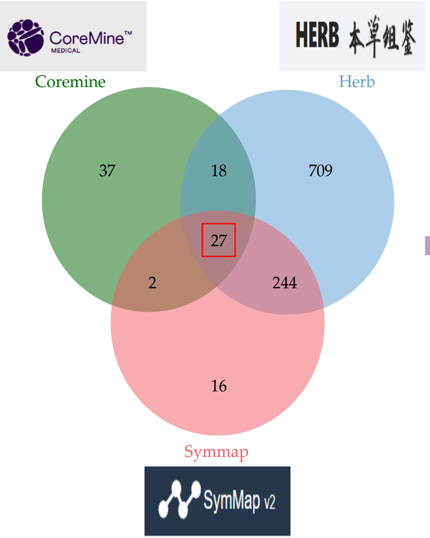

List of core herbs

银杏叶(Ginkgo biloba leaf) 白果(Ginkgo biloba fruit) 积雪草(Centella asiatica)
钩藤(Uncaria rhynchophylla) 郁金(Curcuma aromatica) 益智(Alpinia oxyphylla)
五味子(Schisandra chinensis)淫羊藿(Epimedium brevicornu) 山茱萸(Cornus officinalis)
丹参(Cornus officinalis) 巴戟天(Morinda officinalis) 厚朴(Houpoea officinalis)
黄芩(Scutellaria baicalensis) 黄连(Coptis chinensis) 肉桂(Cinnamomum cassia)
玉米须(Zea mays L. style and stigma)
莲子心(Nelumbo nucifera Gaertn, the dried young leaves and radicles of mature seeds)
吴茱萸(Tetradium ruticarpum) 干姜(Zingiber oj-jicinale Rosc.rhizome)
火麻仁(Cannabis sativa L. seed)
葛花(Pueraria lobata（Willd.）Ohwi, P. thomsonii Benth.flower)
荷叶(Ligularia sibirica) 枸杞子(Lycium chinense Miller fruit) 杜仲(Eucommia ulmoides)
石榴皮(Punica granatum L dried peel) 余甘子(Phyllanthus emblica)
连翘(Forsythia suspensa)
