# Supplementary figures and images for: Identification and Exploration of Immunity‐Related Genes and Natural Products for Alzheimer's Disease Based on Bioinformatics, Molecular Docking, and Molecular Dynamics
Source: Immun Inflamm Dis. 2025 Apr 7;13(4):e70166. doi: 10.1002/iid3.70166 (PMC11973734; doi:10.1002/iid3.70166)

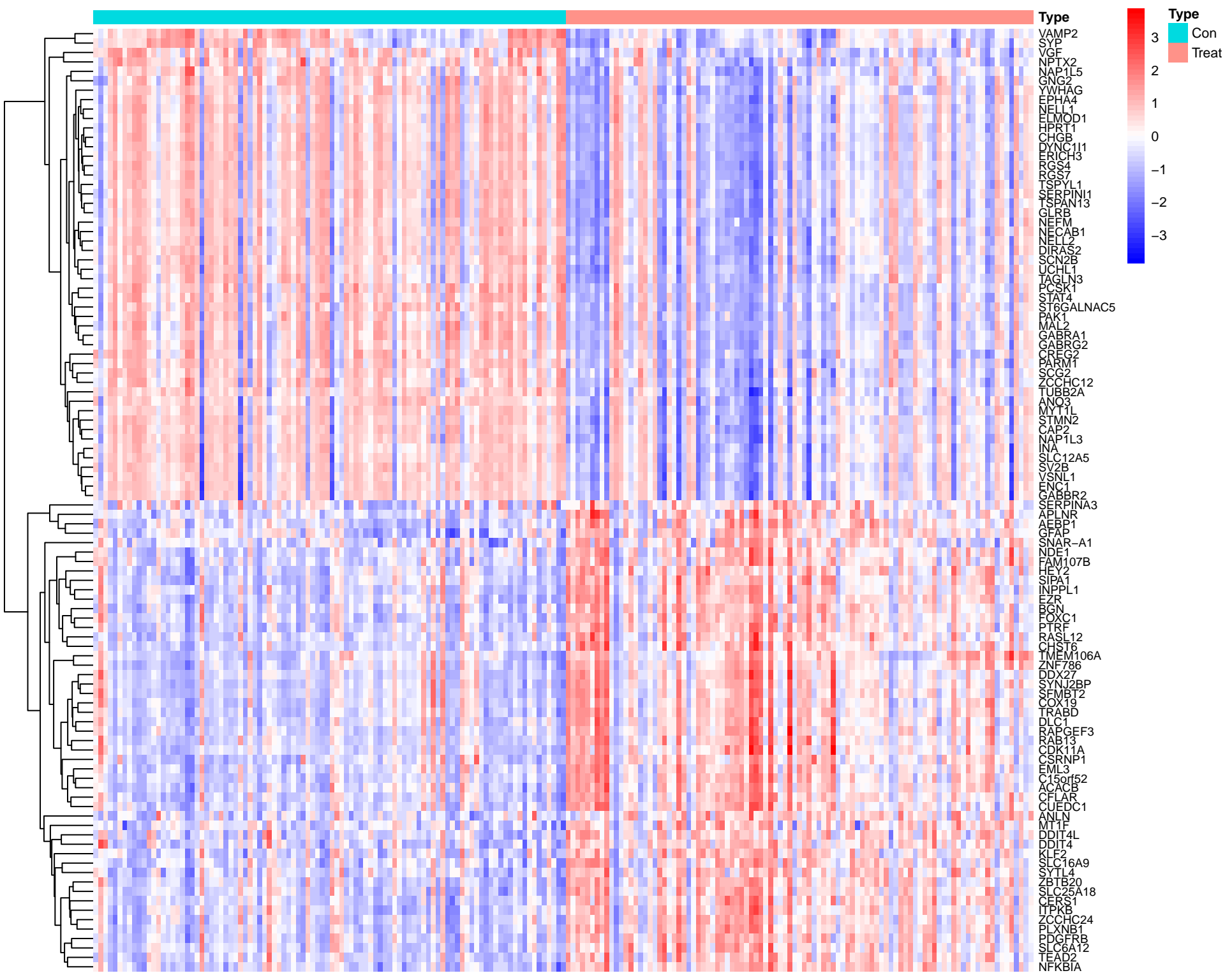

Supplement: Supplementary file 1 — Supporting information. [file IID3-13-e70166-s001.zip › Supplementary materials/S1-DEGS/1.GSE132903/heatmap.pdf]

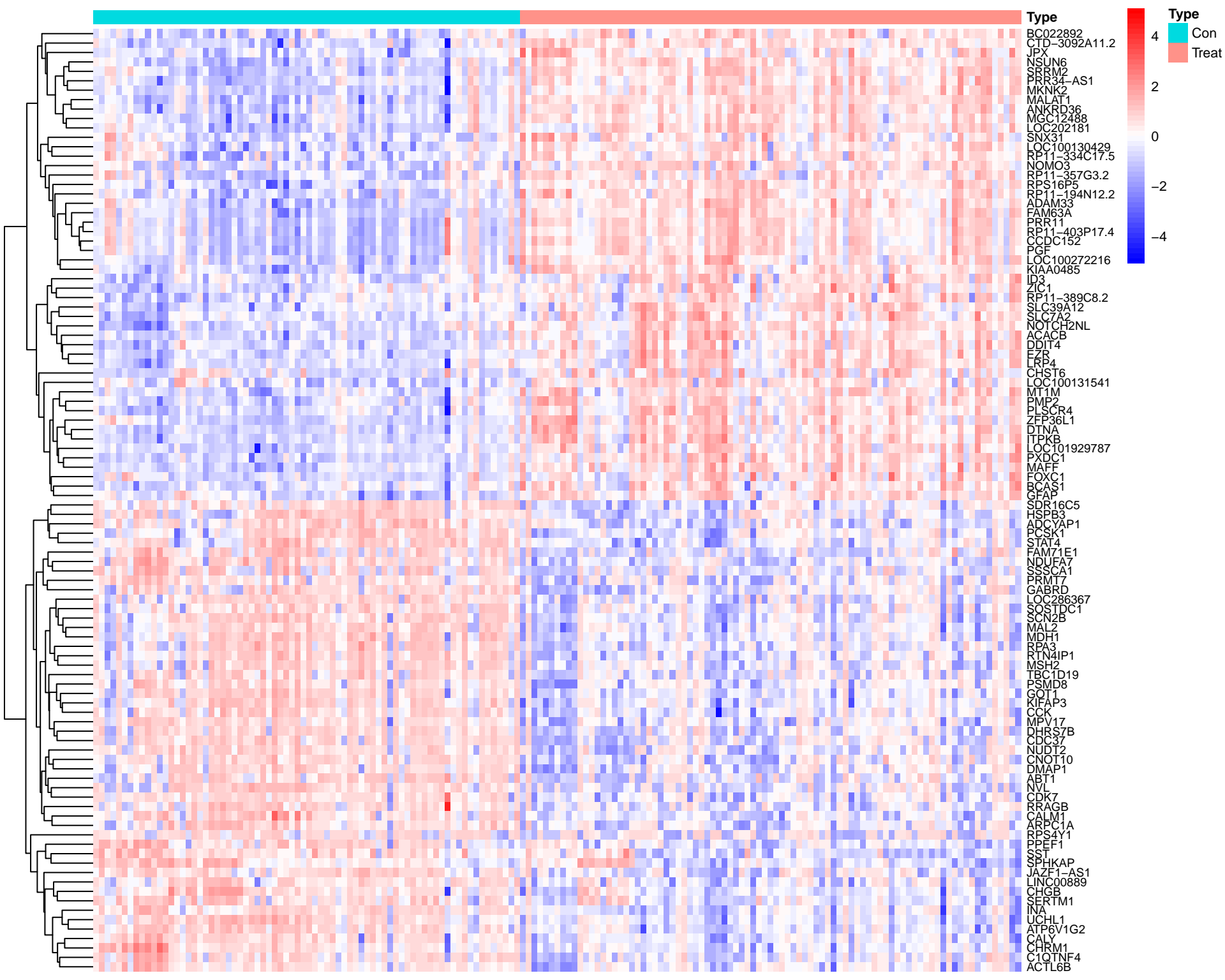

Supplement: Supplementary file 1 — Supporting information. [file IID3-13-e70166-s001.zip › Supplementary materials/S1-DEGS/1.GSE5281/heatmap.pdf]

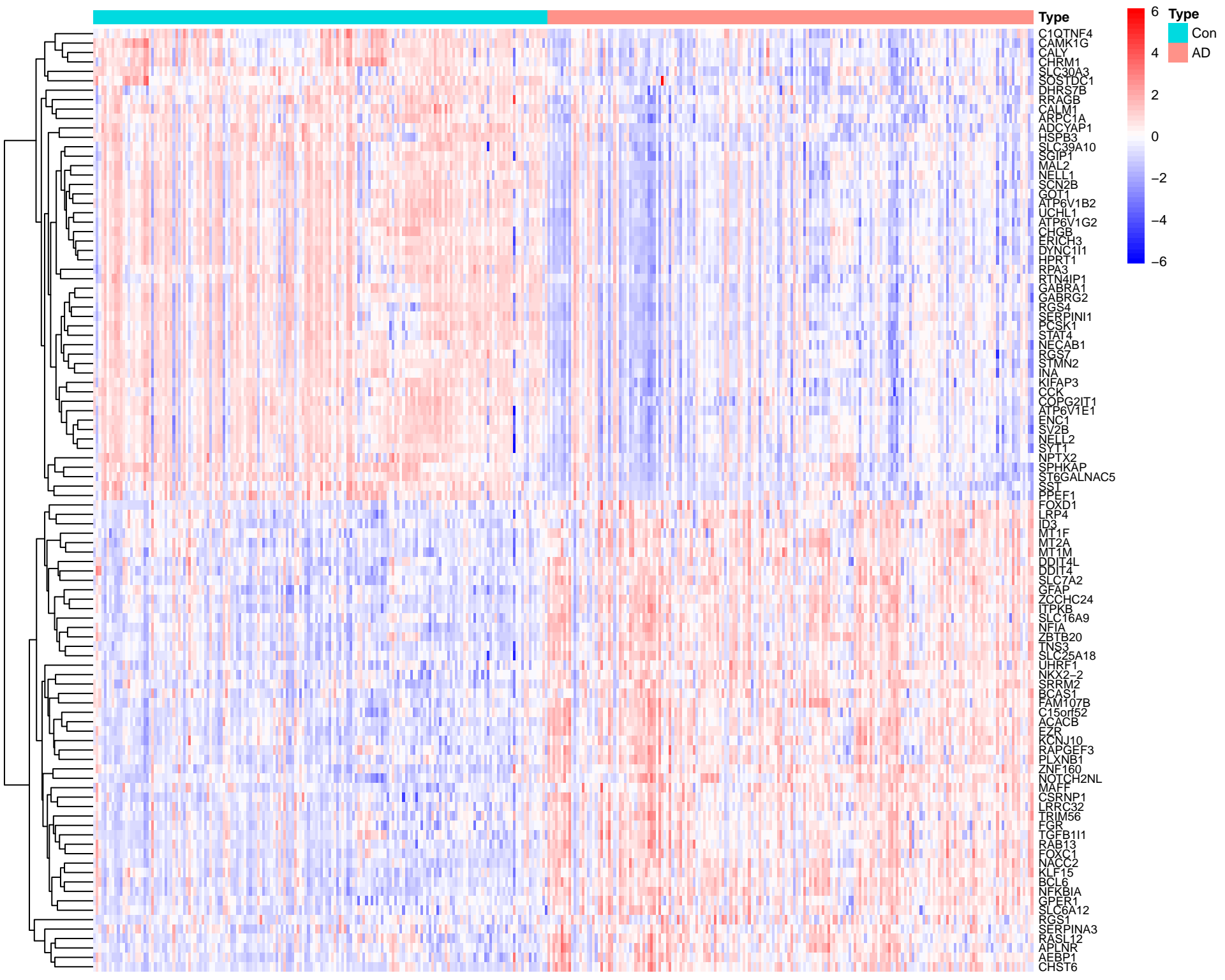

Supplement: Supplementary file 1 — Supporting information. [file IID3-13-e70166-s001.zip › Supplementary materials/S1-DEGS/3.mergediff-train/heatmap.pdf]

**Module membership vs. gene significance**  
**cor=0.38, p=0.006**

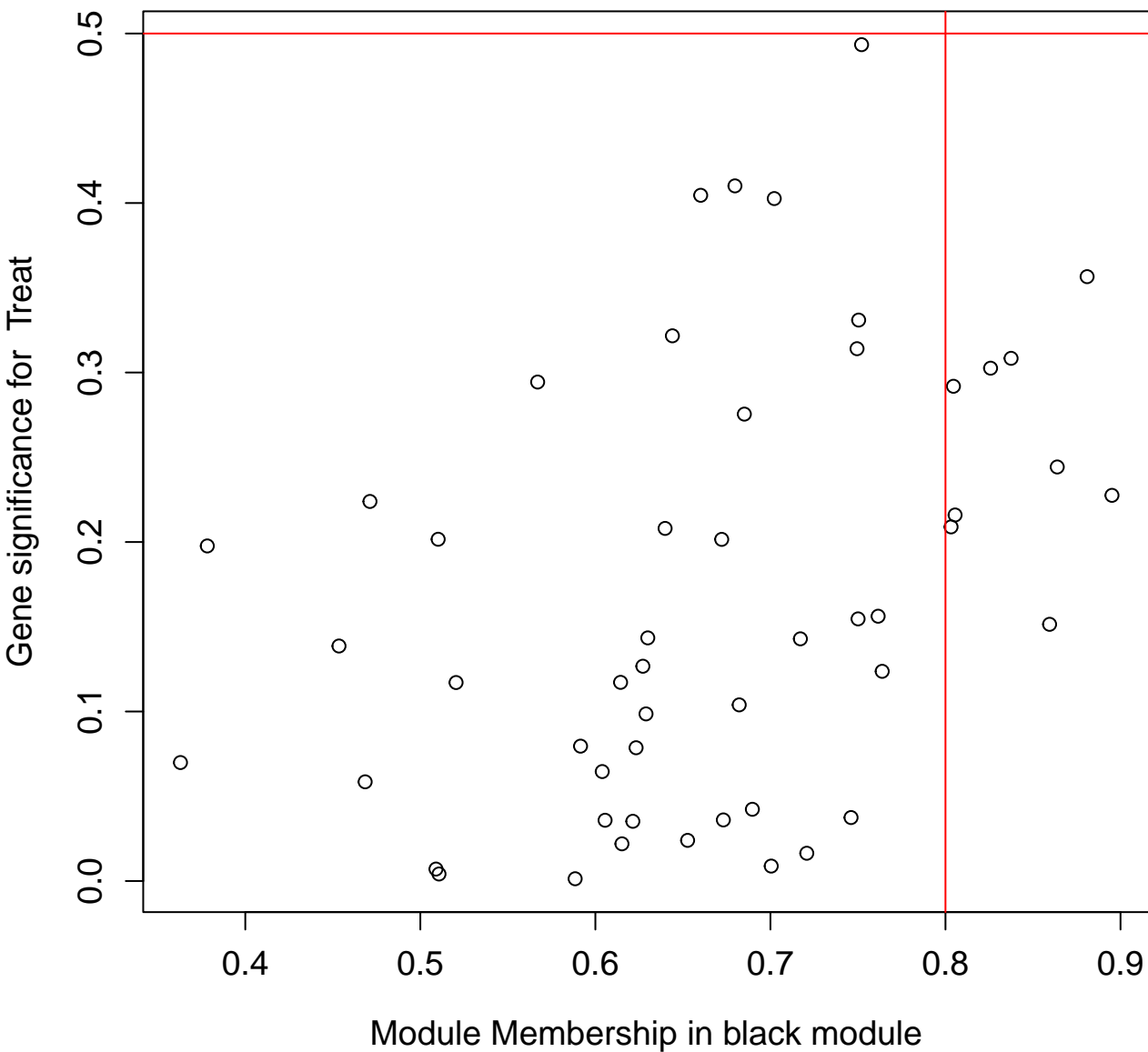

Supplement: Supplementary file 1 — Supporting information. [file IID3-13-e70166-s001.zip › Supplementary materials/S2-WGCNA/10_Treat_black.pdf]

**Module membership vs. gene significance**  
**cor=0.68, p=5.7e-185**

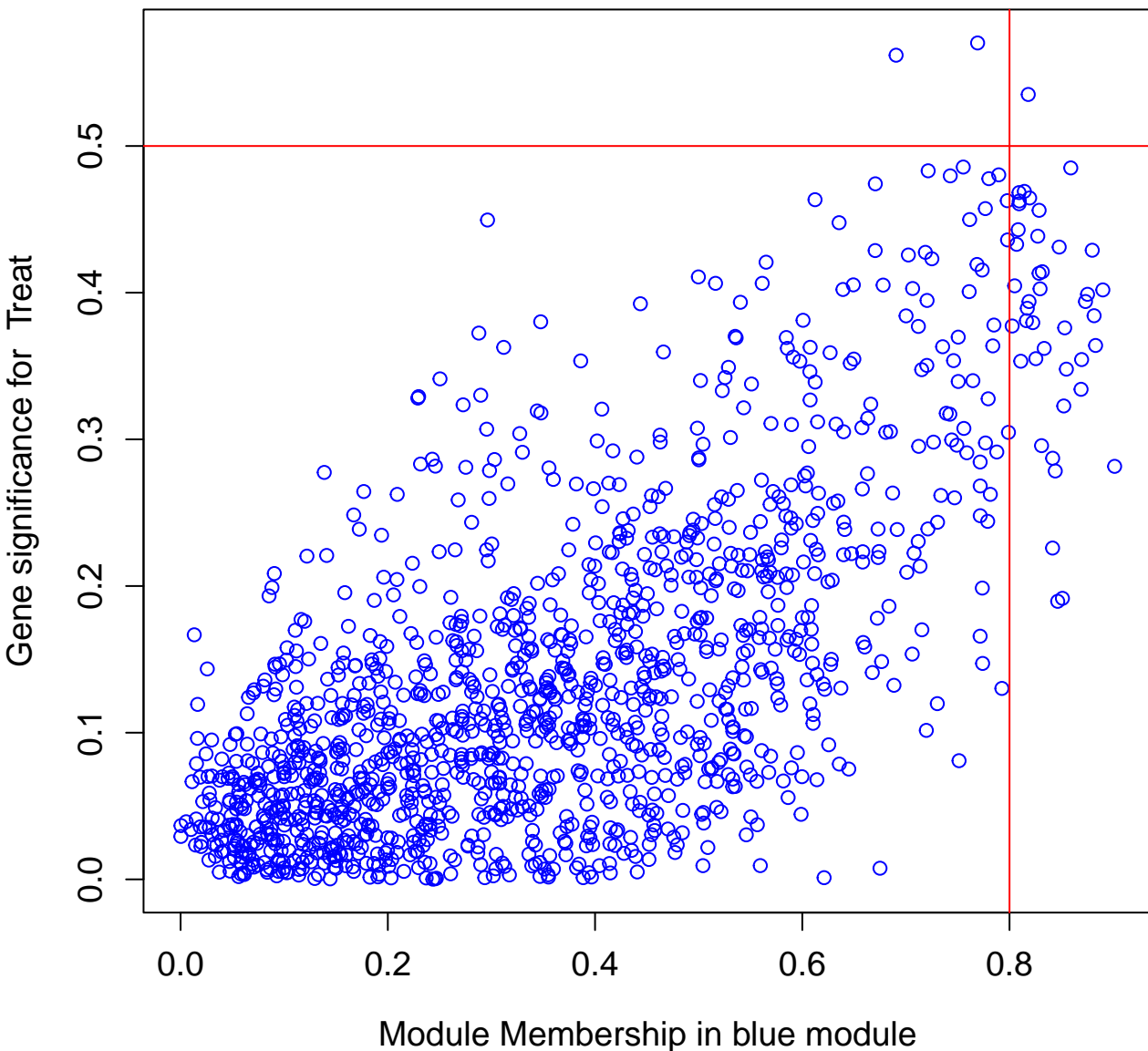

Supplement: Supplementary file 1 — Supporting information. [file IID3-13-e70166-s001.zip › Supplementary materials/S2-WGCNA/10_Treat_blue.pdf]

**Module membership vs. gene significance**  
**cor=0.31, p=1.4e-14**

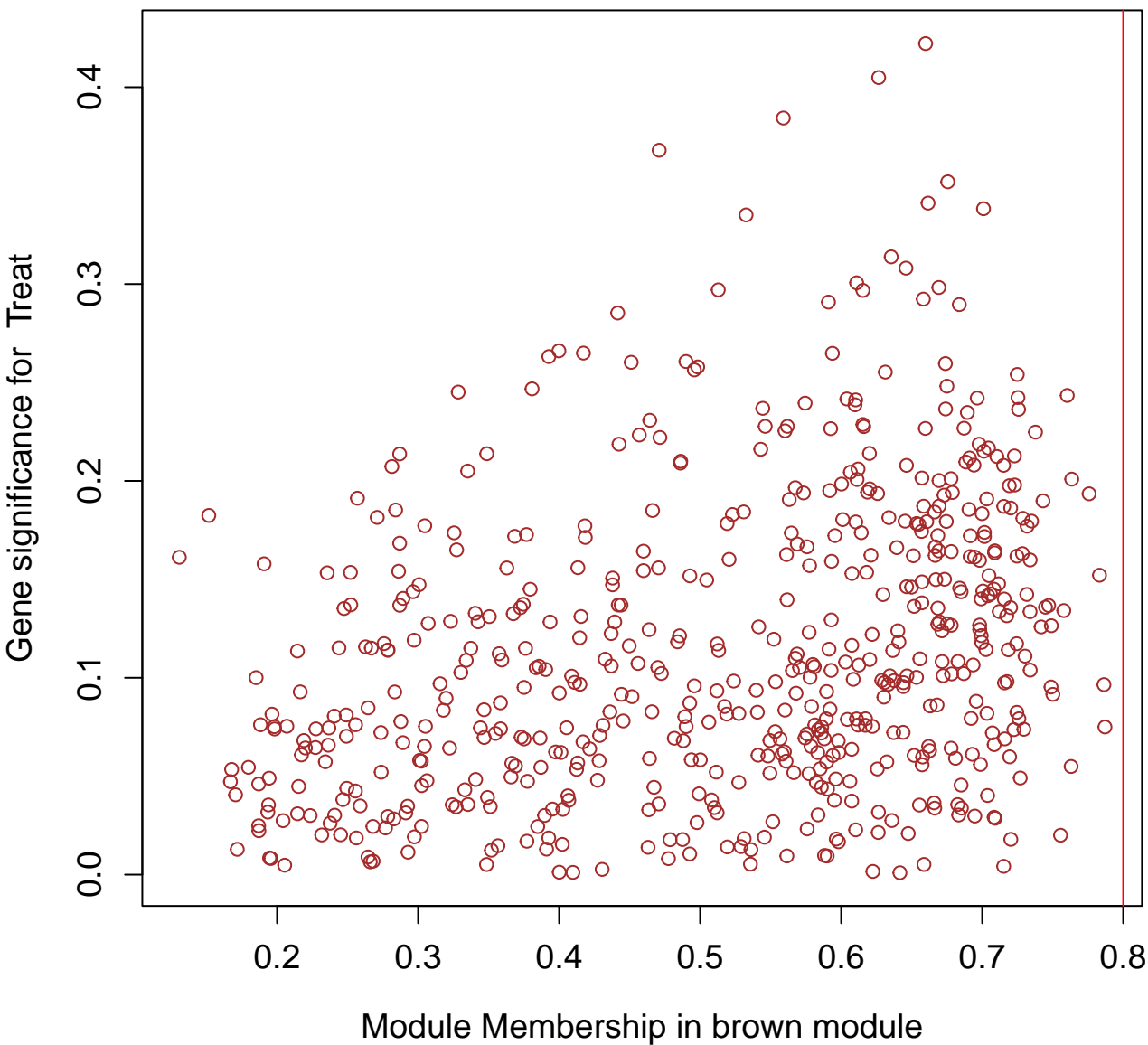

Supplement: Supplementary file 1 — Supporting information. [file IID3-13-e70166-s001.zip › Supplementary materials/S2-WGCNA/10_Treat_brown.pdf]

**Module membership vs. gene significance**  
**cor=0.55, p=1.7e-16**

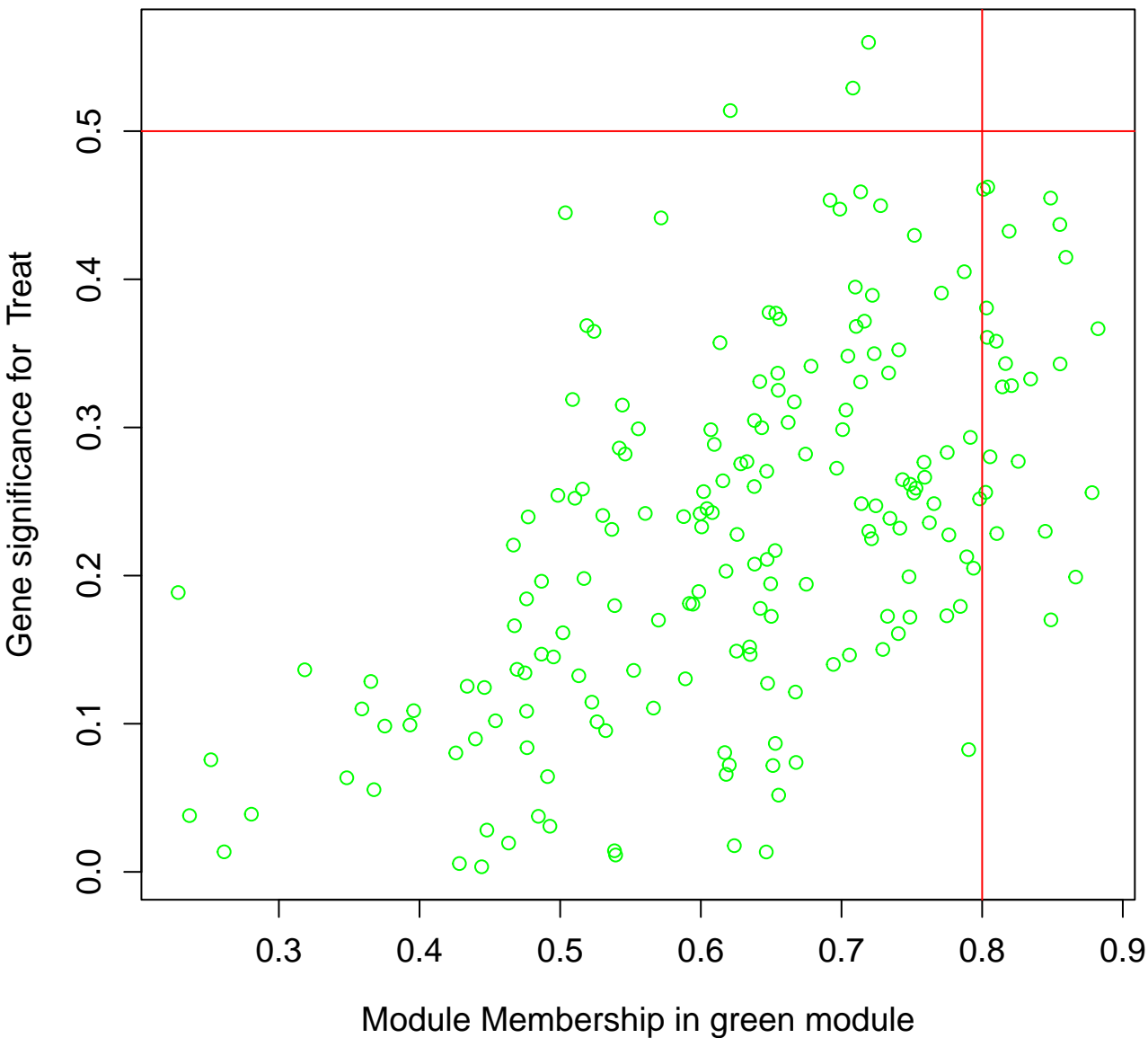

Supplement: Supplementary file 1 — Supporting information. [file IID3-13-e70166-s001.zip › Supplementary materials/S2-WGCNA/10_Treat_green.pdf]

**Module membership vs. gene significance**  
**cor=0.22, p=0.03**

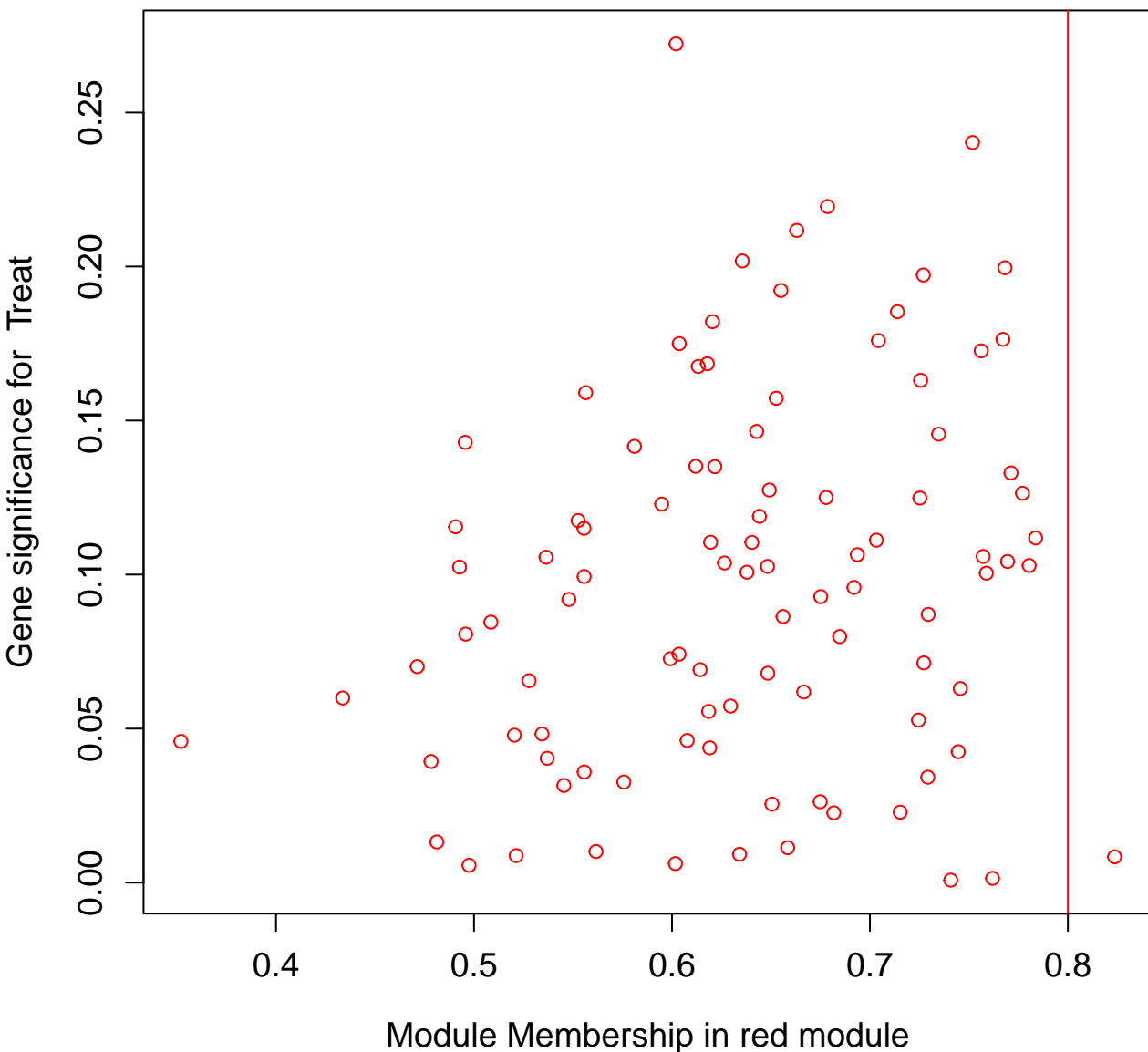

Supplement: Supplementary file 1 — Supporting information. [file IID3-13-e70166-s001.zip › Supplementary materials/S2-WGCNA/10_Treat_red.pdf]

**Module membership vs. gene significance**  
**cor=0.83,  $p < 1e-200$**

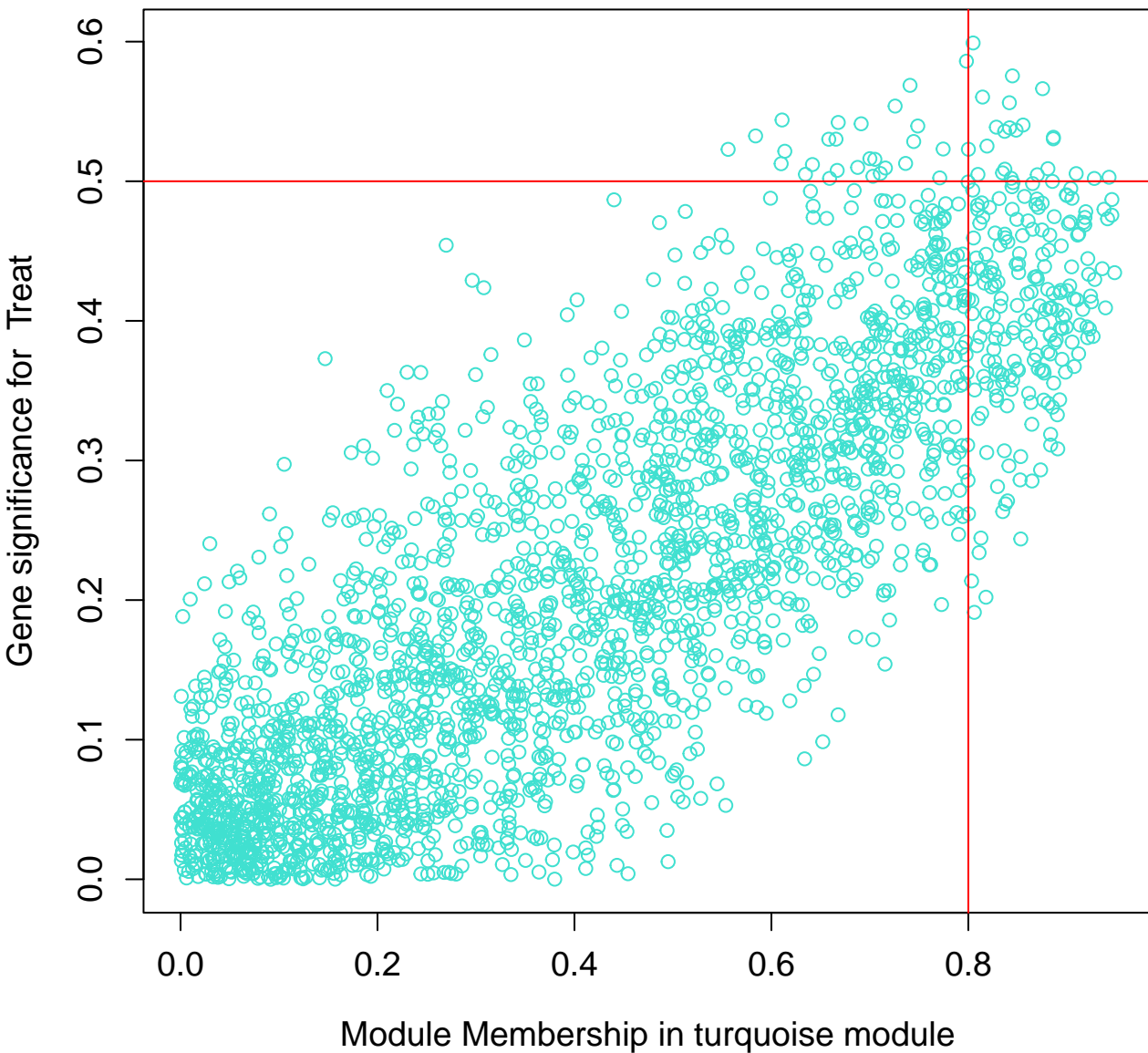

Supplement: Supplementary file 1 — Supporting information. [file IID3-13-e70166-s001.zip › Supplementary materials/S2-WGCNA/10_Treat_turquoise.pdf]

**Module membership vs. gene significance**  
**cor=0.36, p=2.9e-07**

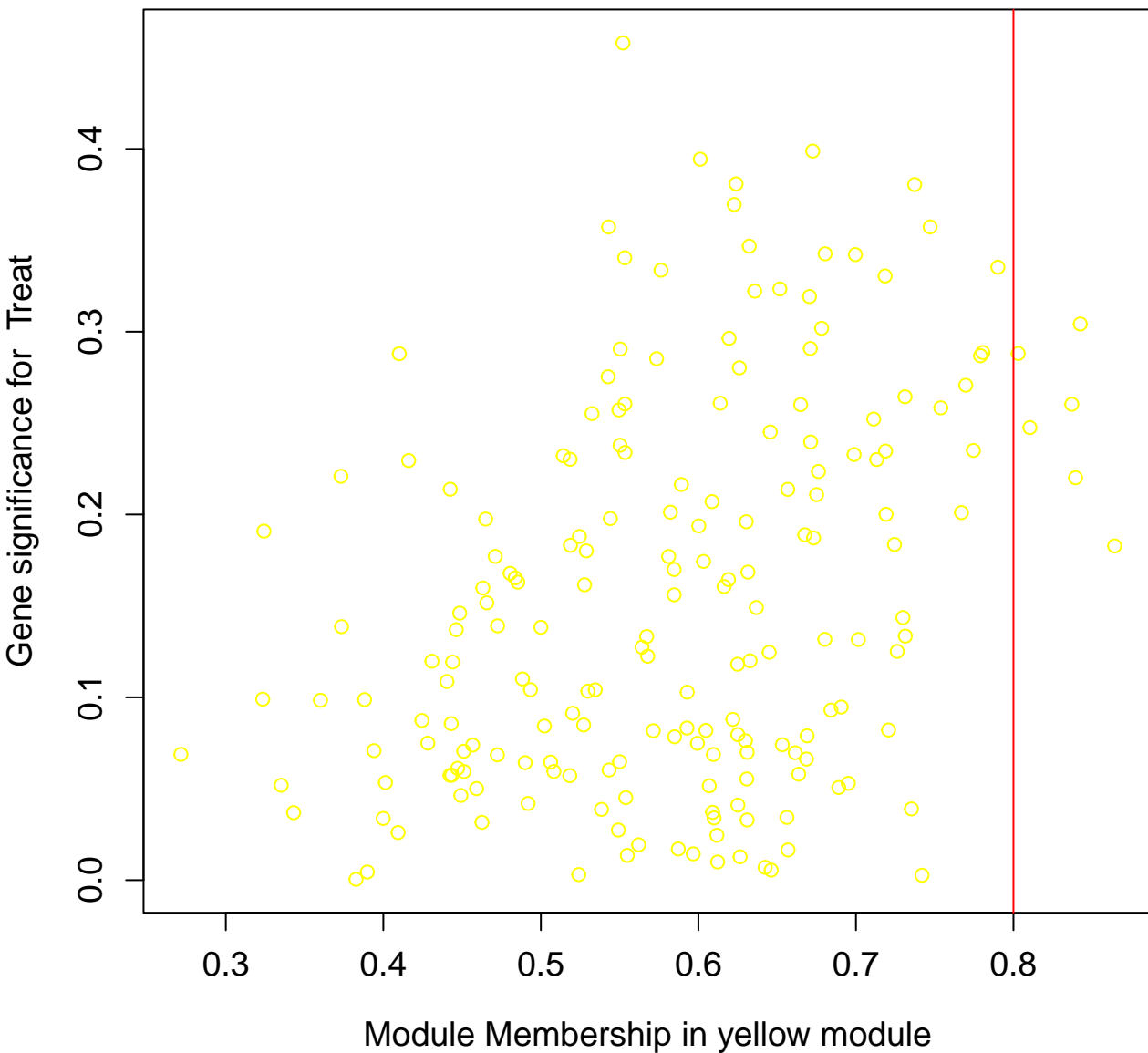

Supplement: Supplementary file 1 — Supporting information. [file IID3-13-e70166-s001.zip › Supplementary materials/S2-WGCNA/10_Treat_yellow.pdf]

# Sample clustering to detect outliers

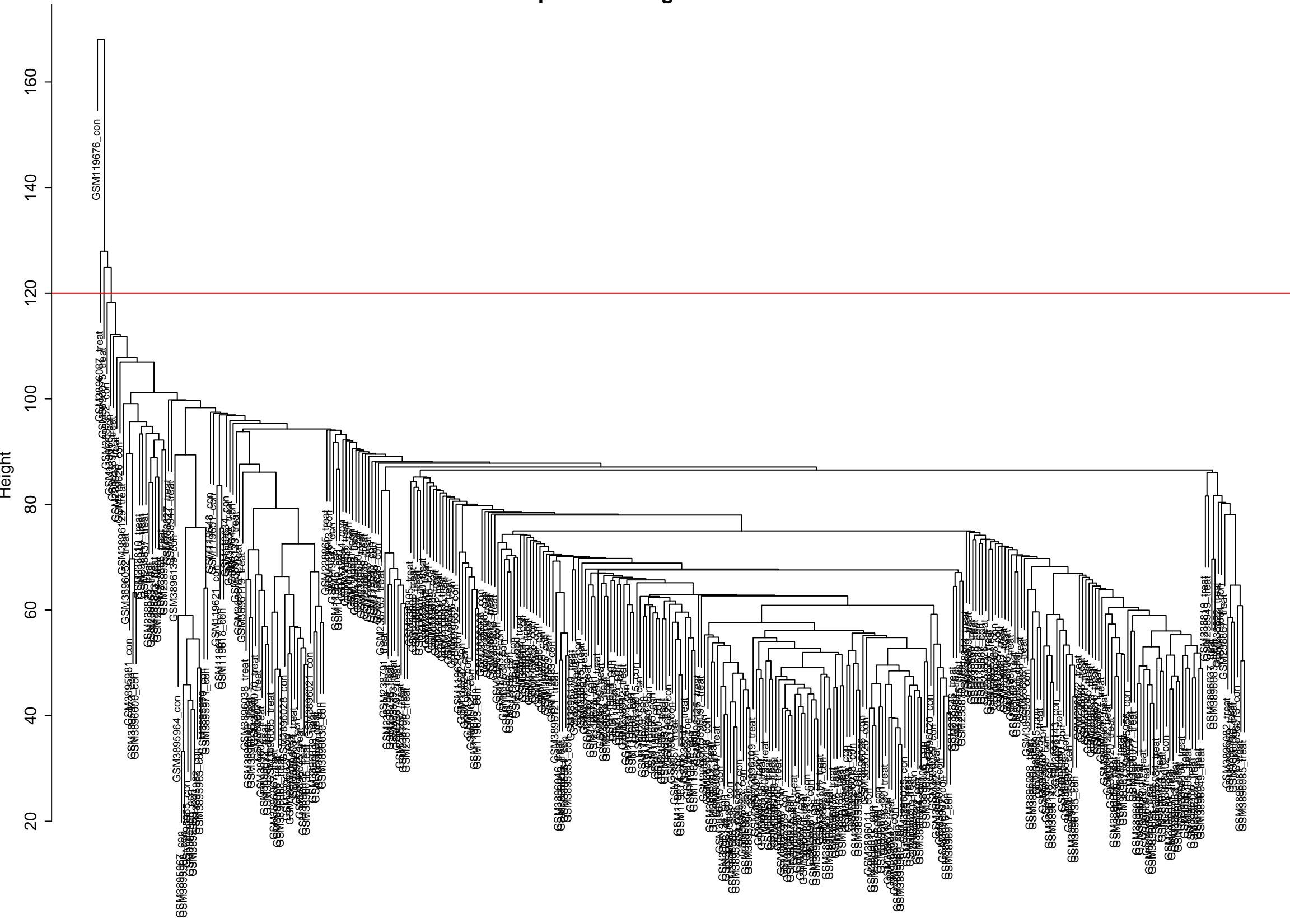

Supplement: Supplementary file 1 — Supporting information. [file IID3-13-e70166-s001.zip › Supplementary materials/S2-WGCNA/1_sample_cluster.pdf]

## Sample dendrogram and trait heatmap

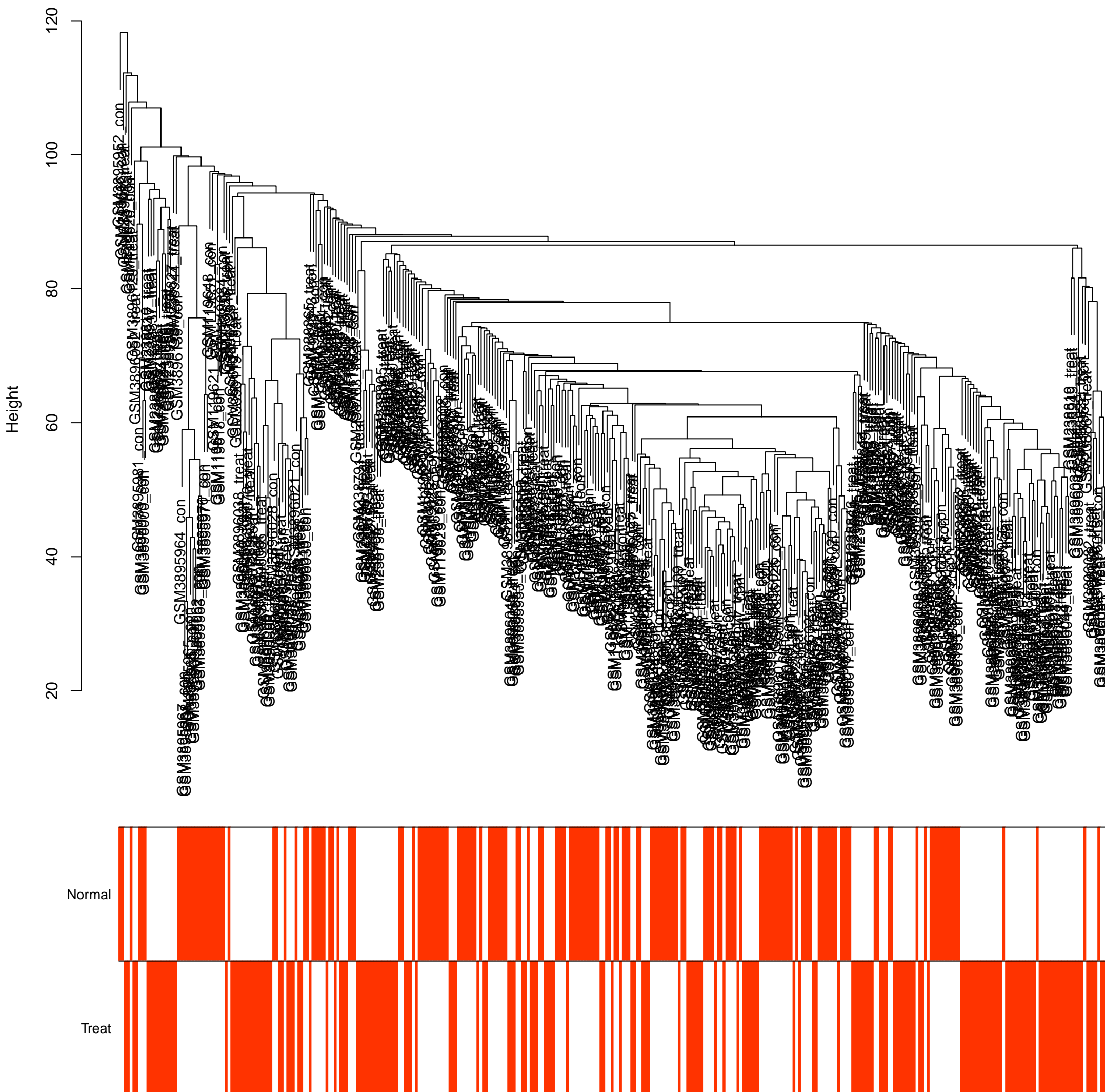

Supplement: Supplementary file 1 — Supporting information. [file IID3-13-e70166-s001.zip › Supplementary materials/S2-WGCNA/2_sample_heatmap.pdf]

### Scale independence

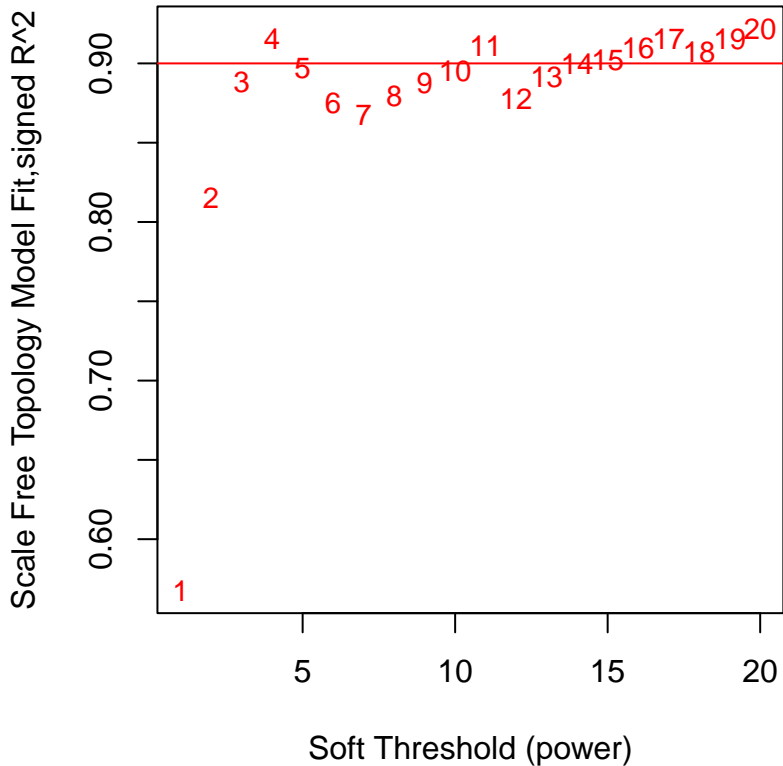

### Mean connectivity

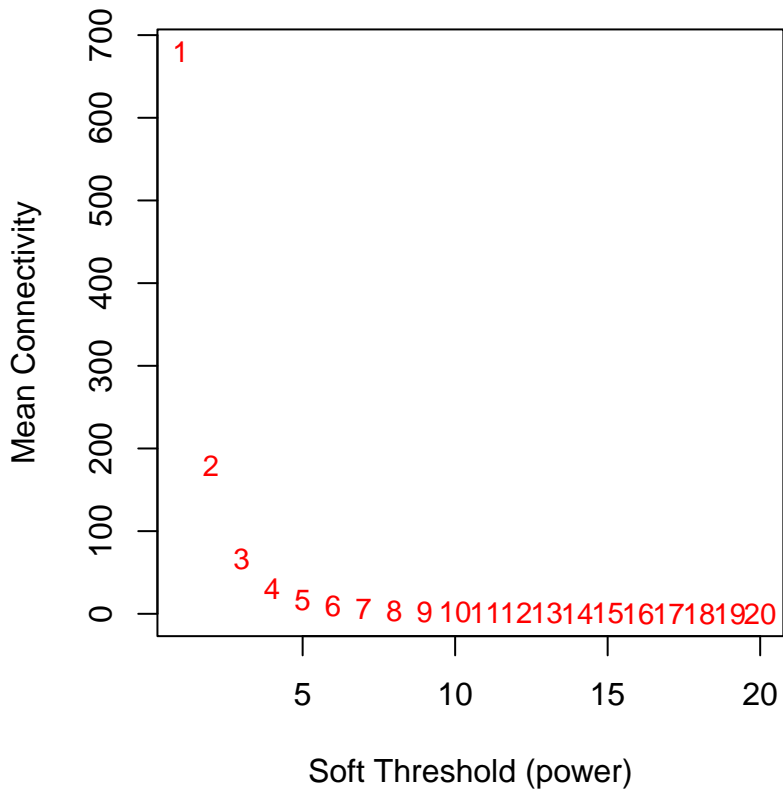

Supplement: Supplementary file 1 — Supporting information. [file IID3-13-e70166-s001.zip › Supplementary materials/S2-WGCNA/3_scale_independence.pdf]

**Histogram of k**

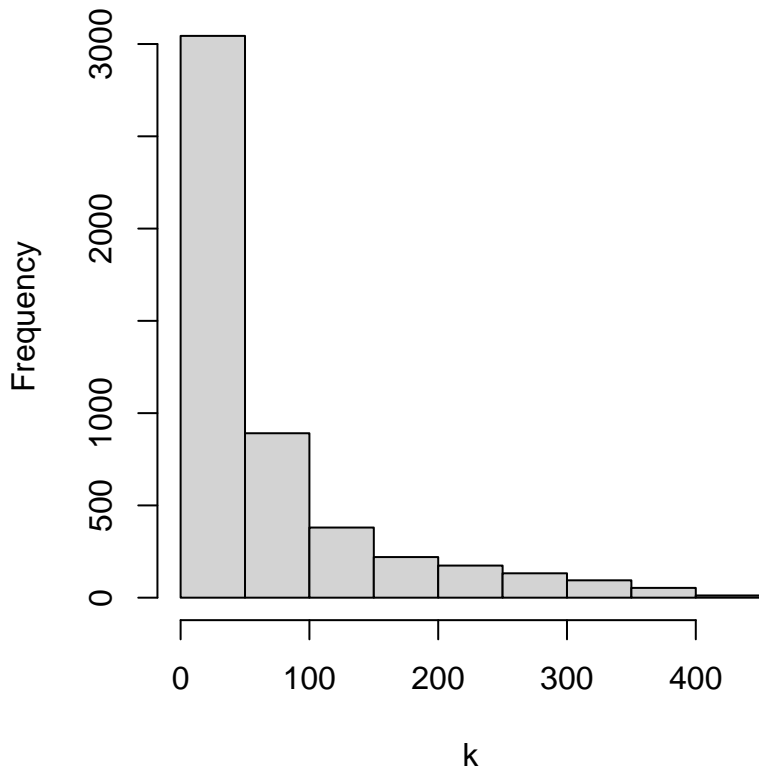

**Check Scale free topology**  
scale  $R^2 = 0.87$  , slope =  $-1.42$

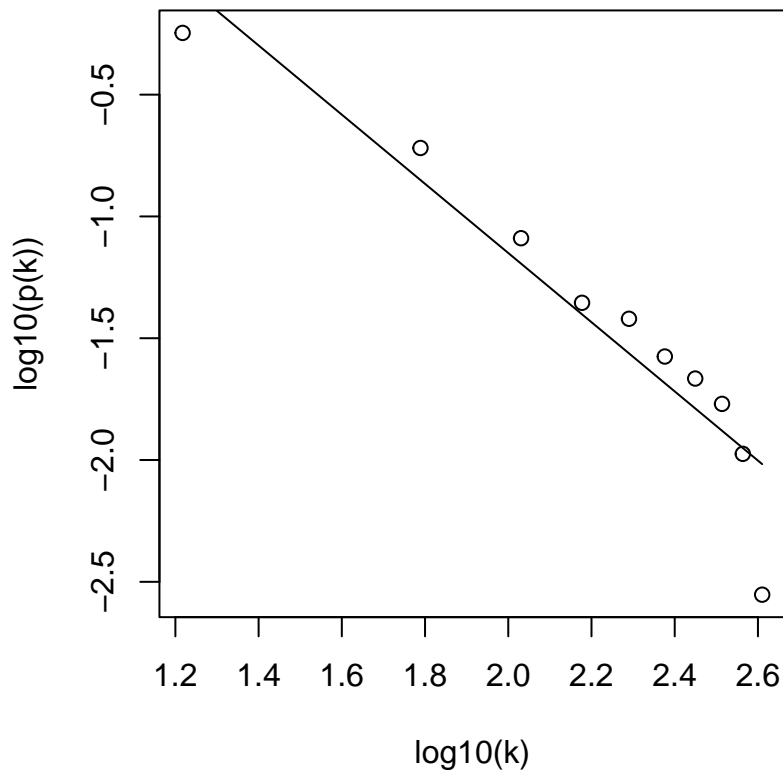

Supplement: Supplementary file 1 — Supporting information. [file IID3-13-e70166-s001.zip › Supplementary materials/S2-WGCNA/3_softConnectivity.pdf]

# Gene clustering on TOM-based dissimilarity

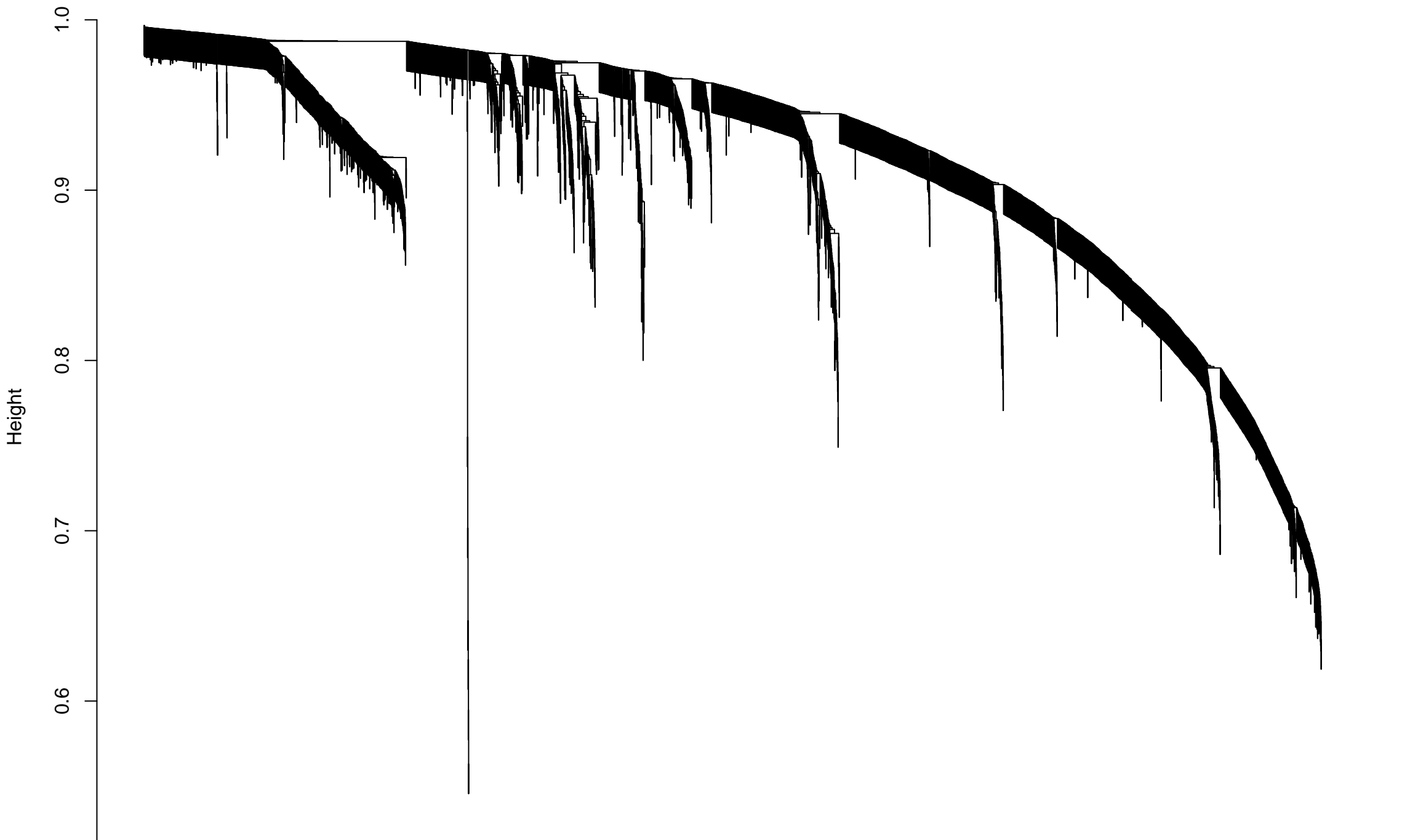

Supplement: Supplementary file 1 — Supporting information. [file IID3-13-e70166-s001.zip › Supplementary materials/S2-WGCNA/4_gene_clustering.pdf]

# Gene dendrogram and module colors

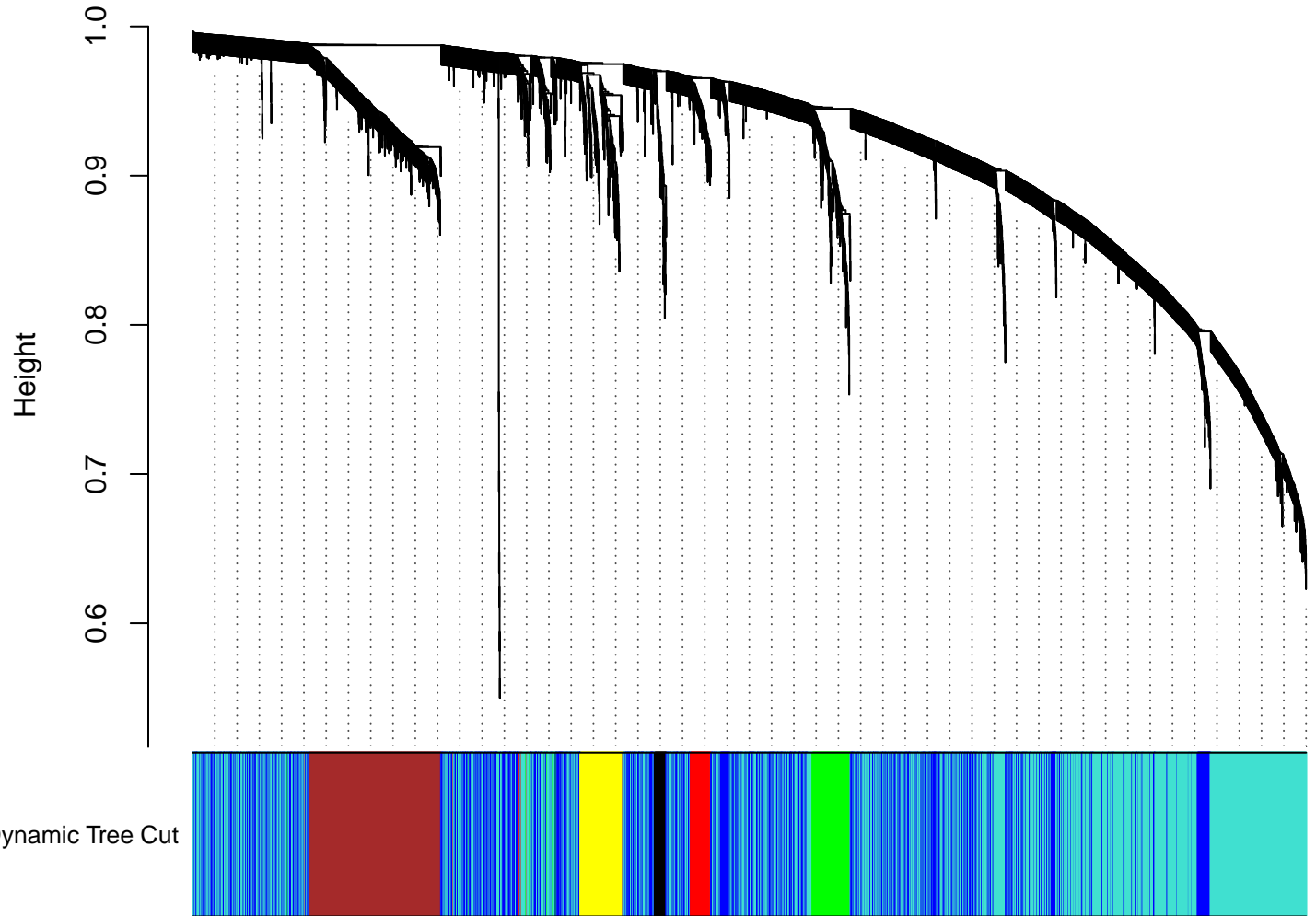

Supplement: Supplementary file 1 — Supporting information. [file IID3-13-e70166-s001.zip › Supplementary materials/S2-WGCNA/5_Dynamic_Tree.pdf]

## Clustering of module eigengenes

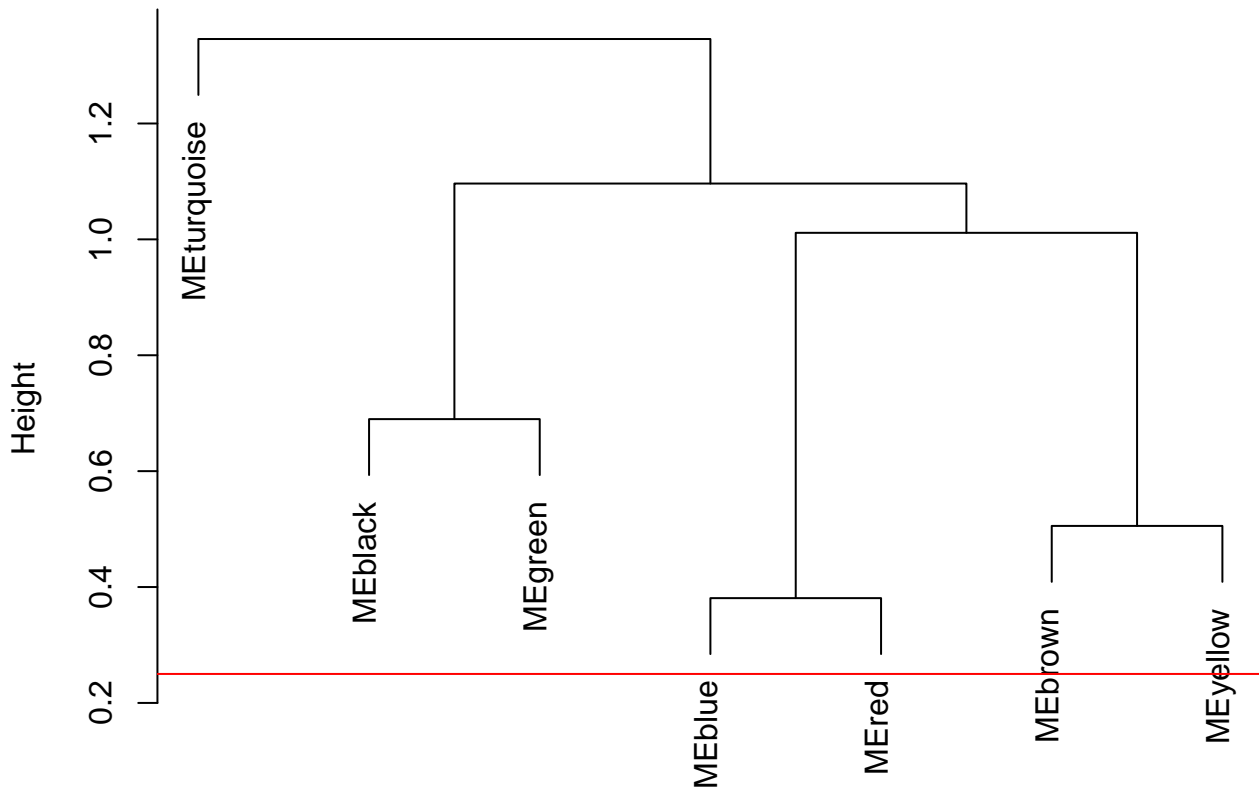

Supplement: Supplementary file 1 — Supporting information. [file IID3-13-e70166-s001.zip › Supplementary materials/S2-WGCNA/6_Clustering_module.pdf]

Gene dendrogram and module colors

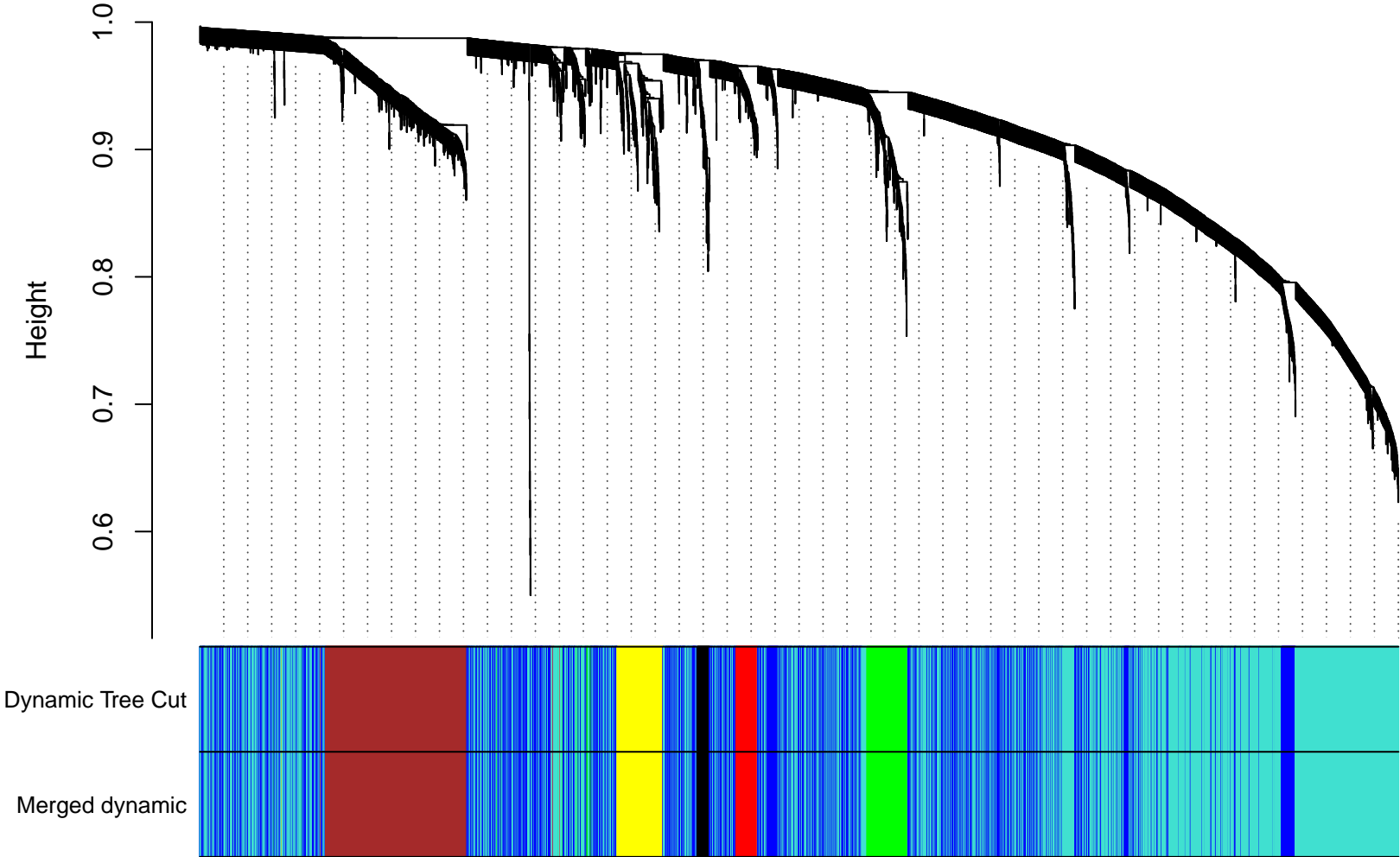

Supplement: Supplementary file 1 — Supporting information. [file IID3-13-e70166-s001.zip › Supplementary materials/S2-WGCNA/7_merged_dynamic.pdf]

## Module-trait relationships

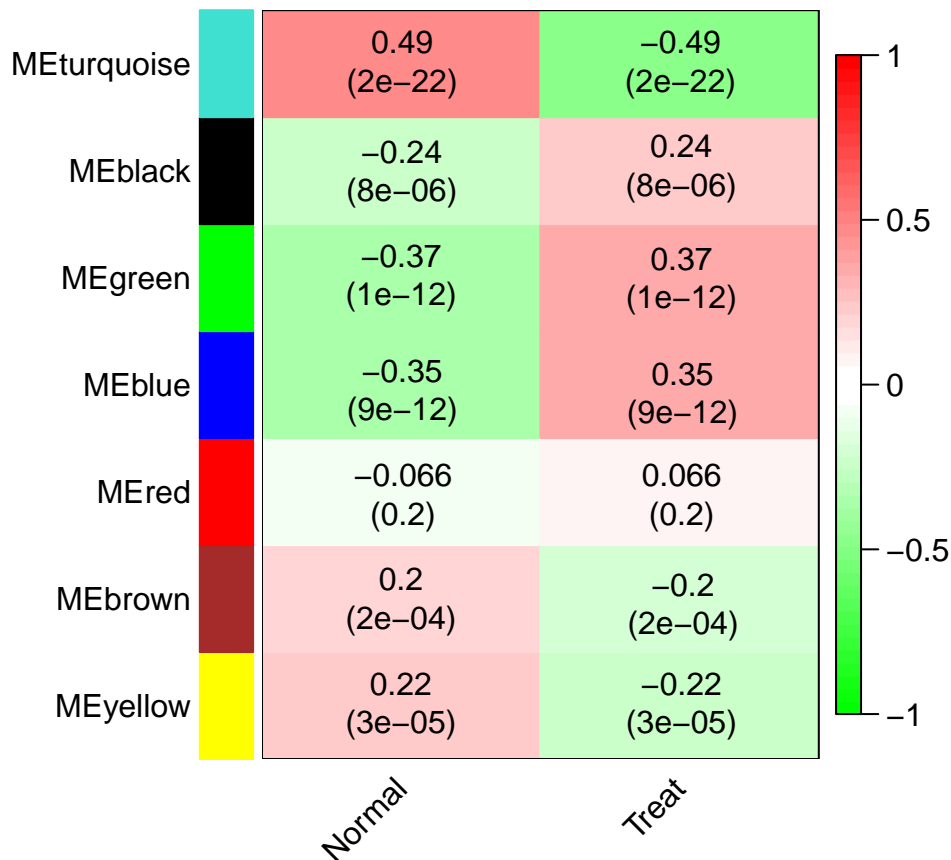

Supplement: Supplementary file 1 — Supporting information. [file IID3-13-e70166-s001.zip › Supplementary materials/S2-WGCNA/8_Module_trait.pdf]

Gene significance across modules, p-value=9.4e-86

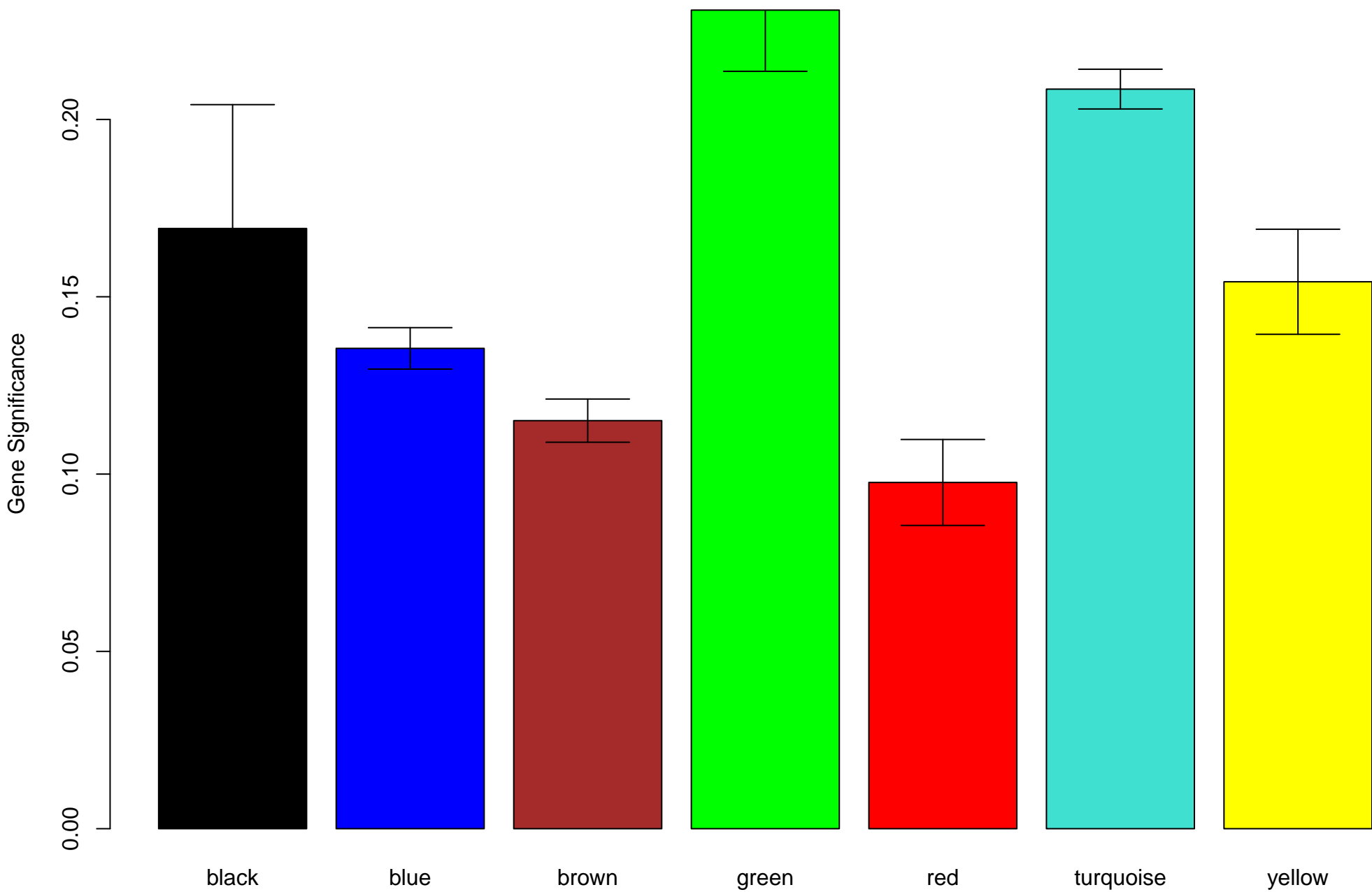

Supplement: Supplementary file 1 — Supporting information. [file IID3-13-e70166-s001.zip › Supplementary materials/S2-WGCNA/9_GeneSignificance.pdf]

Importance

15

10

5

0

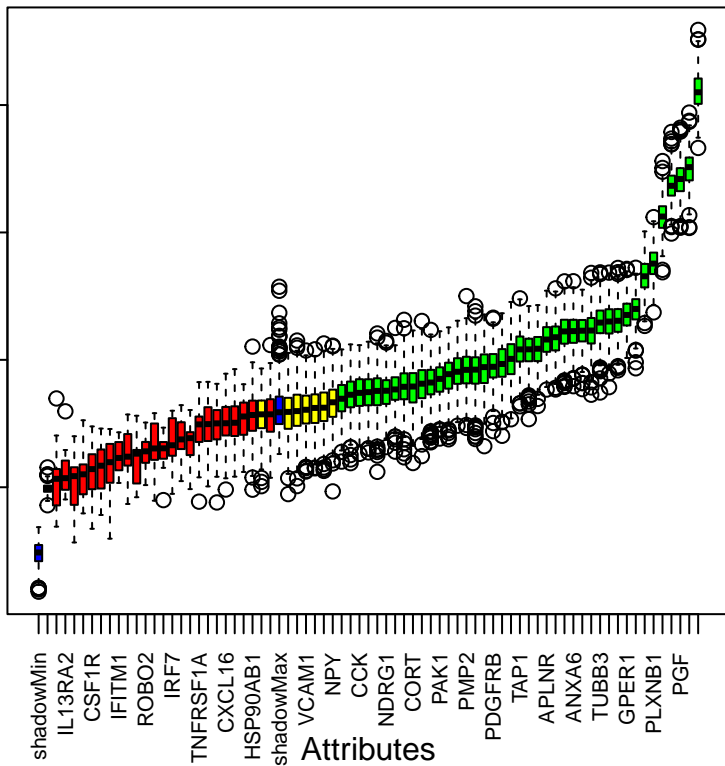

Attributes

Supplement: Supplementary file 1 — Supporting information. [file IID3-13-e70166-s001.zip › Supplementary materials/S4-Machine learning/boruta/A_Boruta.pdf]

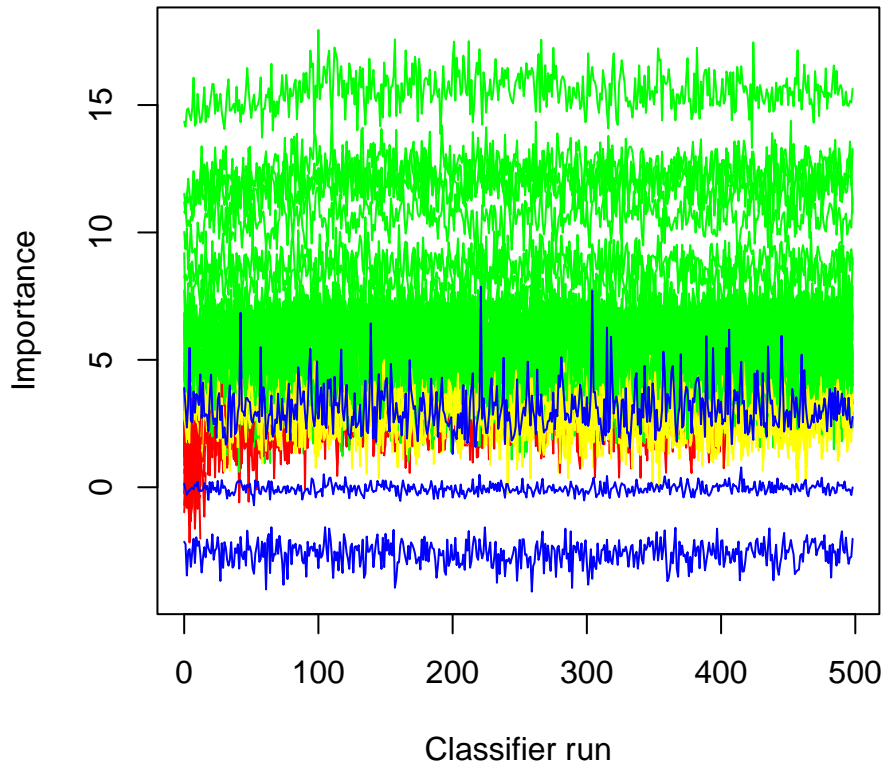

Supplement: Supplementary file 1 — Supporting information. [file IID3-13-e70166-s001.zip › Supplementary materials/S4-Machine learning/boruta/B_Boruta.pdf]

Binomial Deviance

72 72 71 70 70 66 60 57 53 41 32 25 18 9 6 6 5 3 1

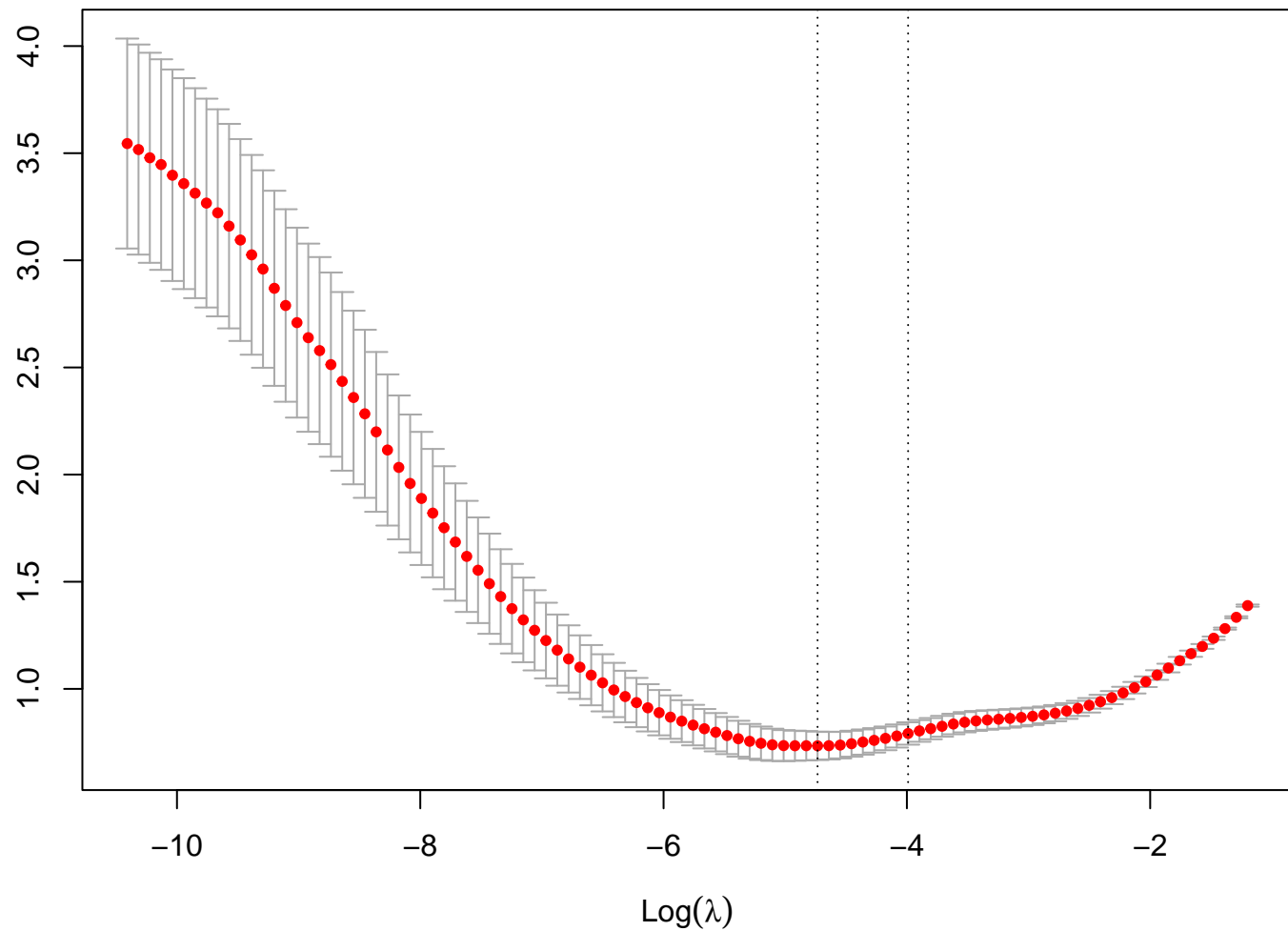

Supplement: Supplementary file 1 — Supporting information. [file IID3-13-e70166-s001.zip › Supplementary materials/S4-Machine learning/LASSO/lasso1.pdf]

Coefficients

Log Lambda

72

70

55

24

5

10

5

0

-5

-10

-8

-6

-4

-2

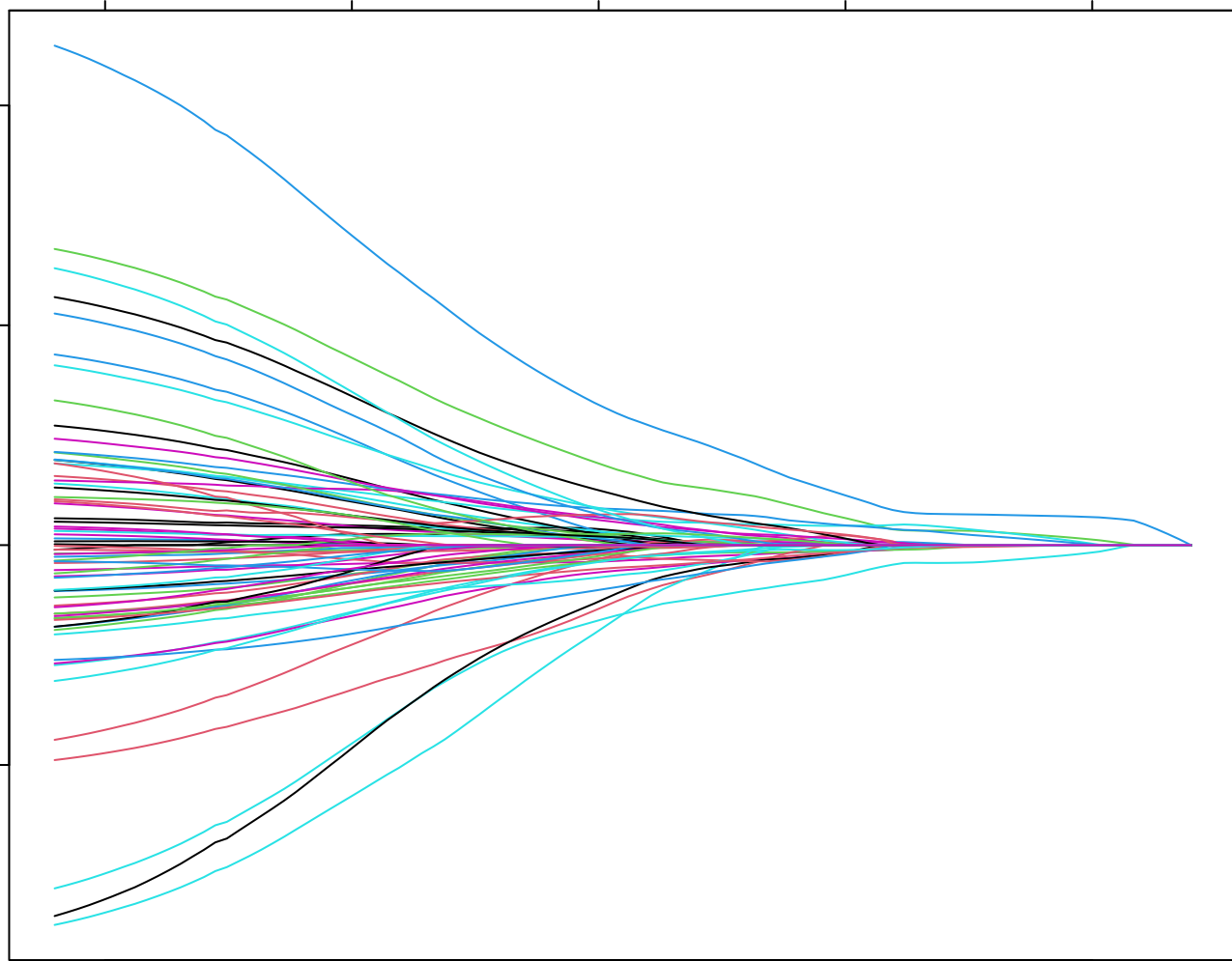

Supplement: Supplementary file 1 — Supporting information. [file IID3-13-e70166-s001.zip › Supplementary materials/S4-Machine learning/LASSO/lasso2.pdf]

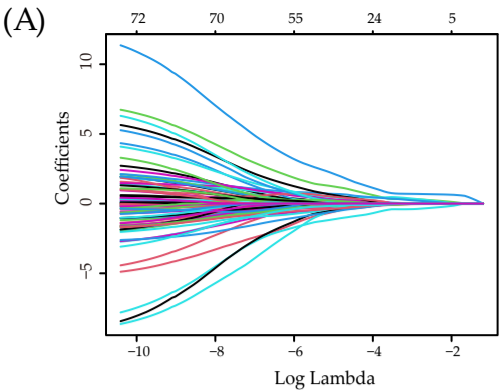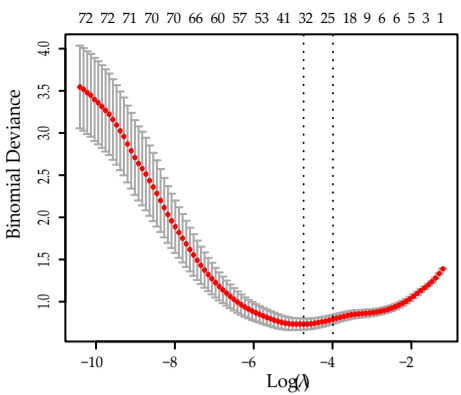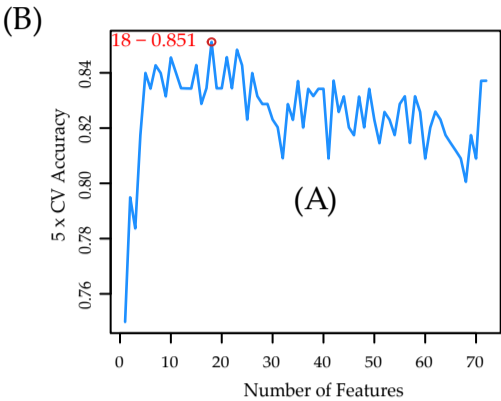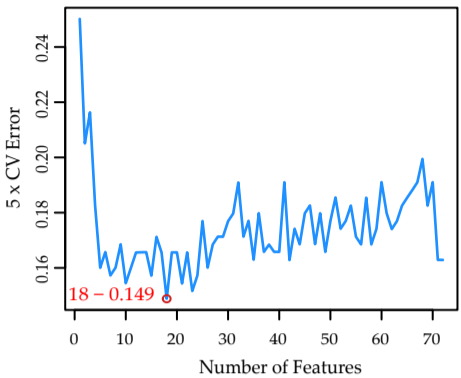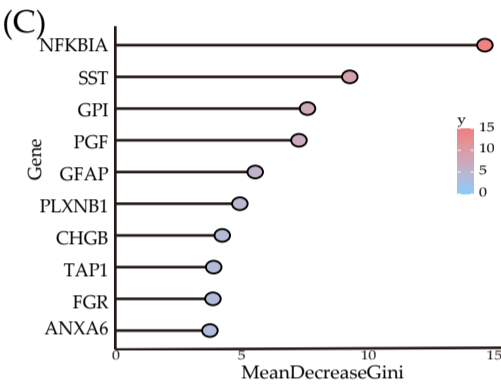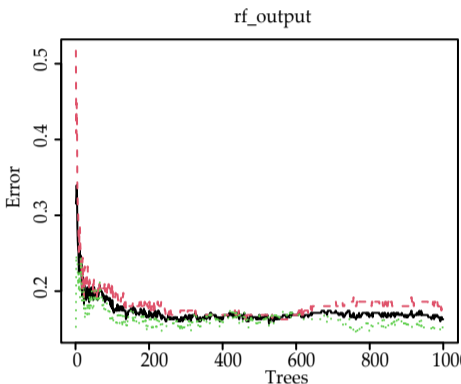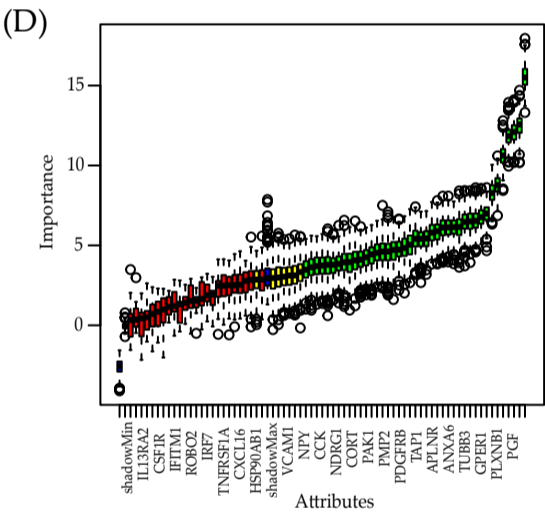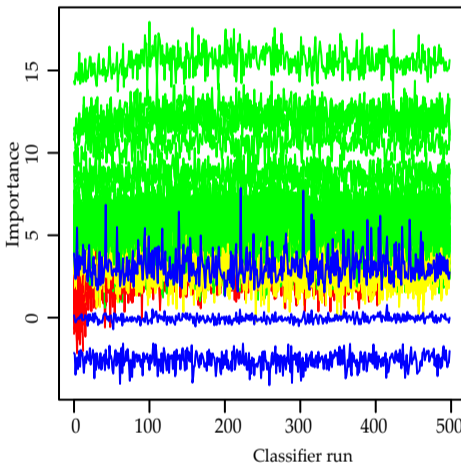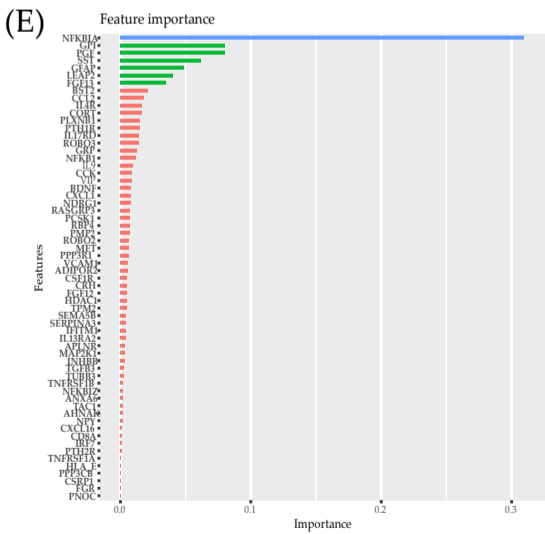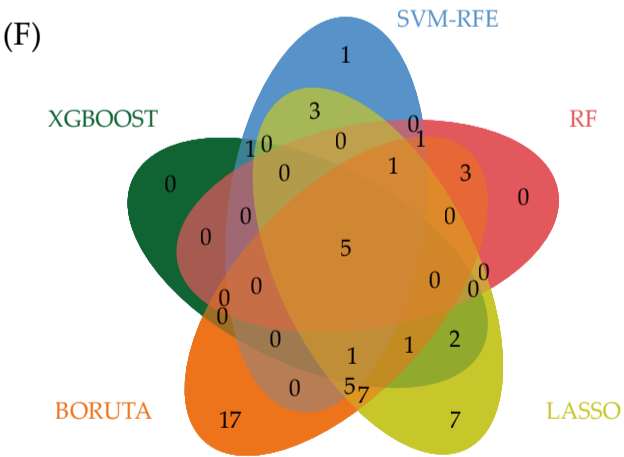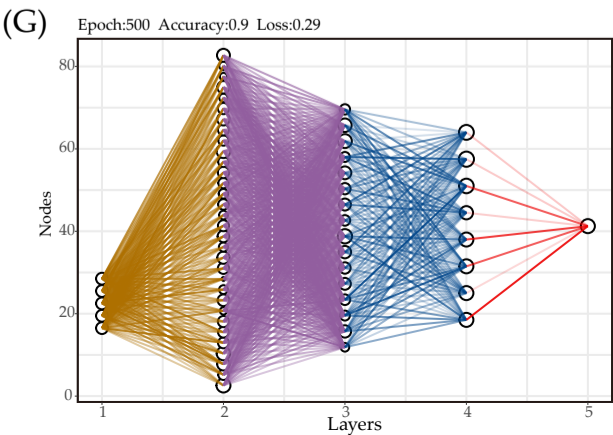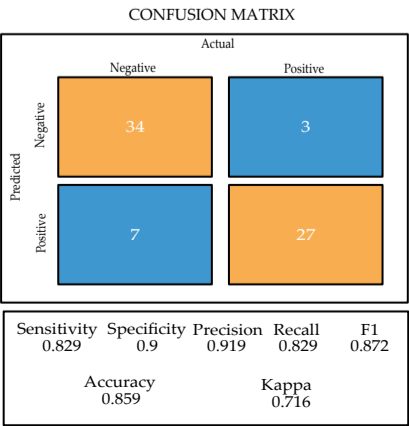

Supplement: Supplementary file 1 — Supporting information. [file IID3-13-e70166-s001.zip › Supplementary materials/S4-Machine learning/machine learning original figure.pdf]

# CONFUSION MATRIX

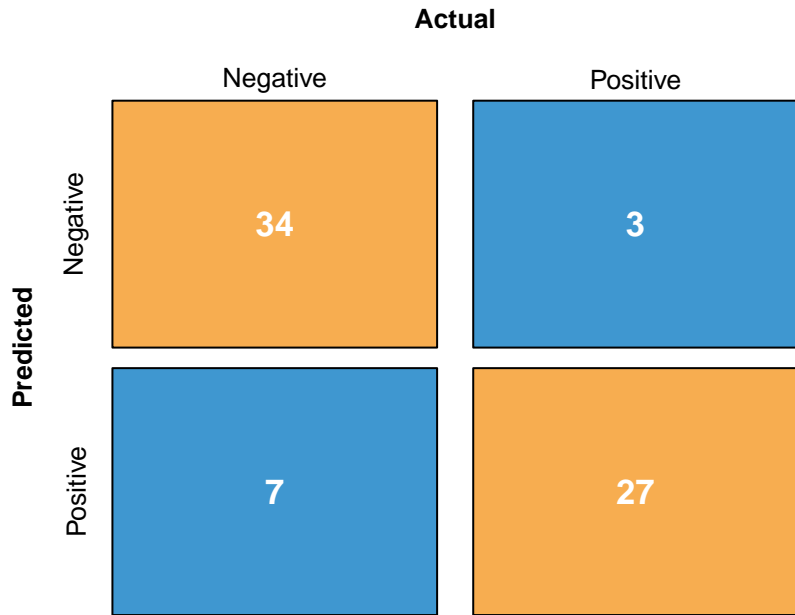

## DETAILS

**Sensitivity**

0.829

**Specificity**

0.9

**Precision**

0.919

**Recall**

0.829

**F1**

0.872

**Accuracy**

0.859

**Kappa**

0.716

Supplement: Supplementary file 1 — Supporting information. [file IID3-13-e70166-s001.zip › Supplementary materials/S4-Machine learning/MLP/conf_matrix.pdf]

Epoch:500 Accuracy:0.9 Loss:0.29

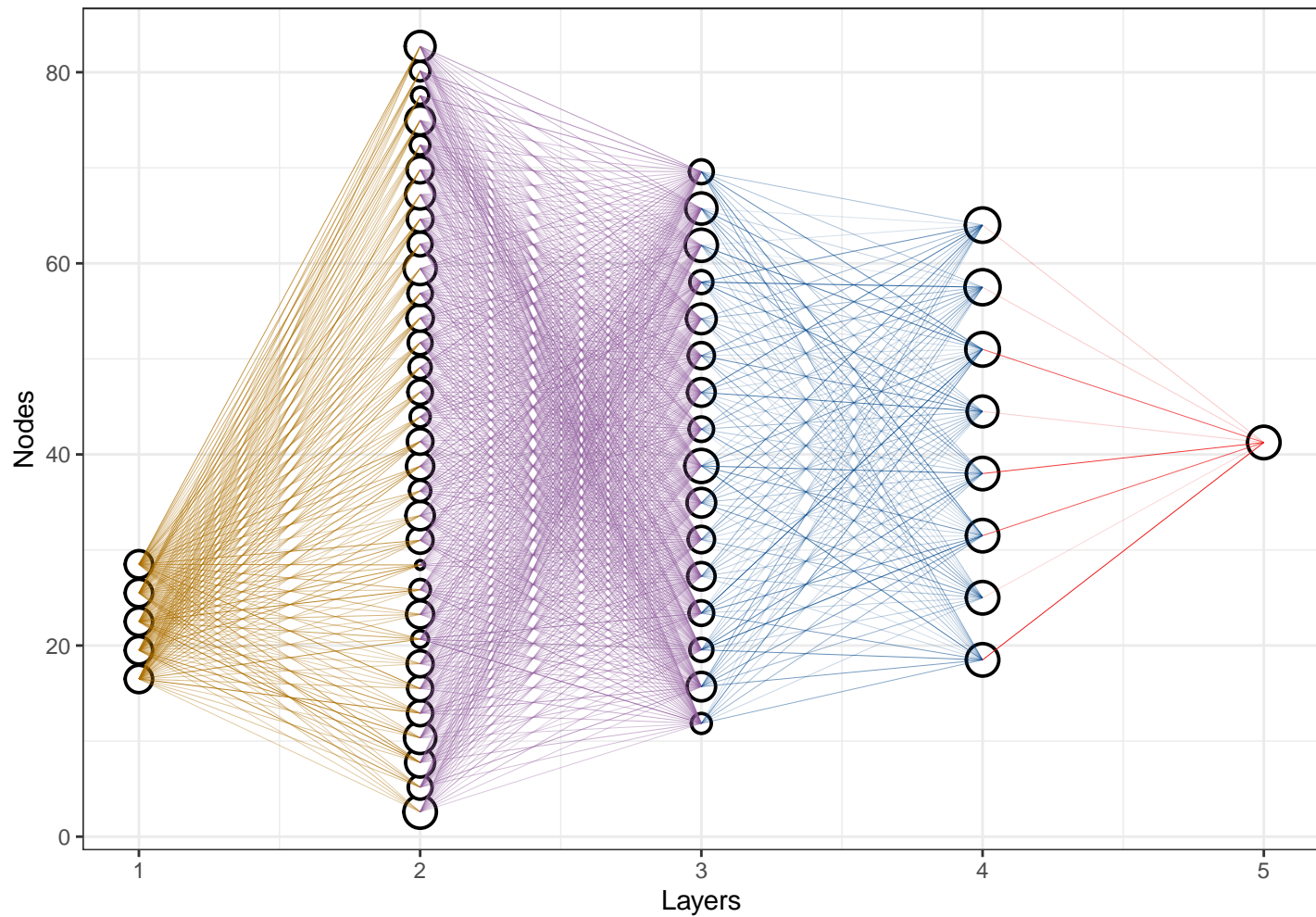

Supplement: Supplementary file 1 — Supporting information. [file IID3-13-e70166-s001.zip › Supplementary materials/S4-Machine learning/MLP/network.pdf]

# Prediction vs. Real Labels

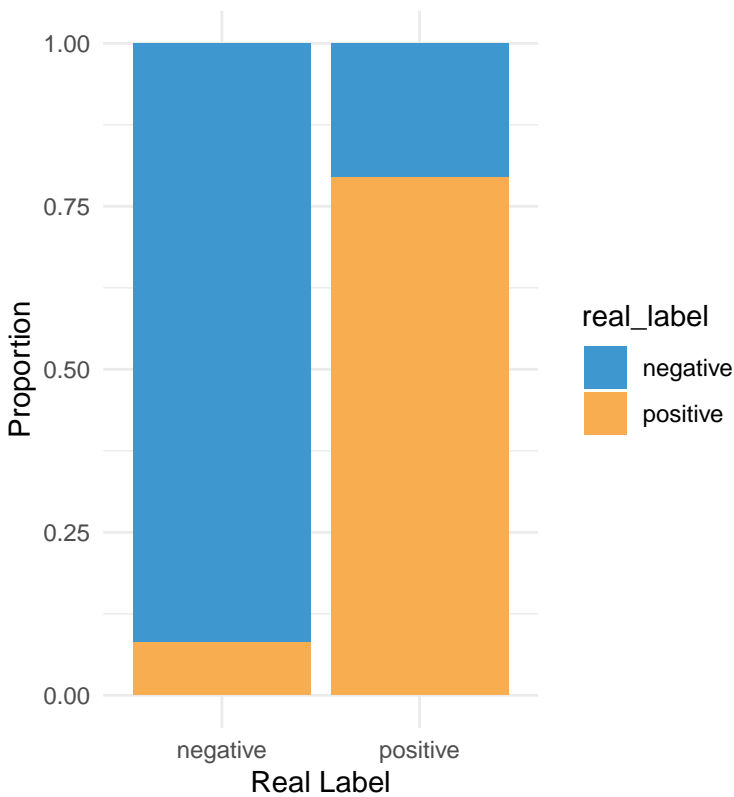

Supplement: Supplementary file 1 — Supporting information. [file IID3-13-e70166-s001.zip › Supplementary materials/S4-Machine learning/MLP/result_ration.pdf]

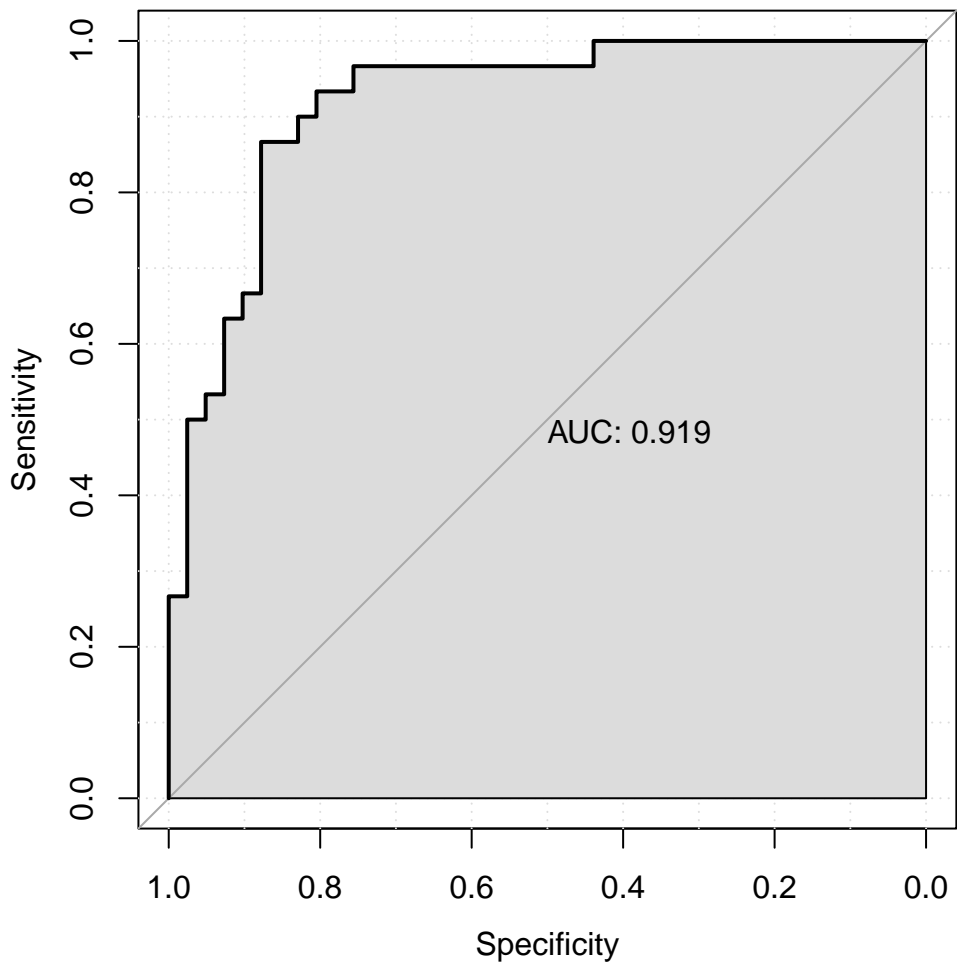

Supplement: Supplementary file 1 — Supporting information. [file IID3-13-e70166-s001.zip › Supplementary materials/S4-Machine learning/MLP/roc.pdf]

rf\_output

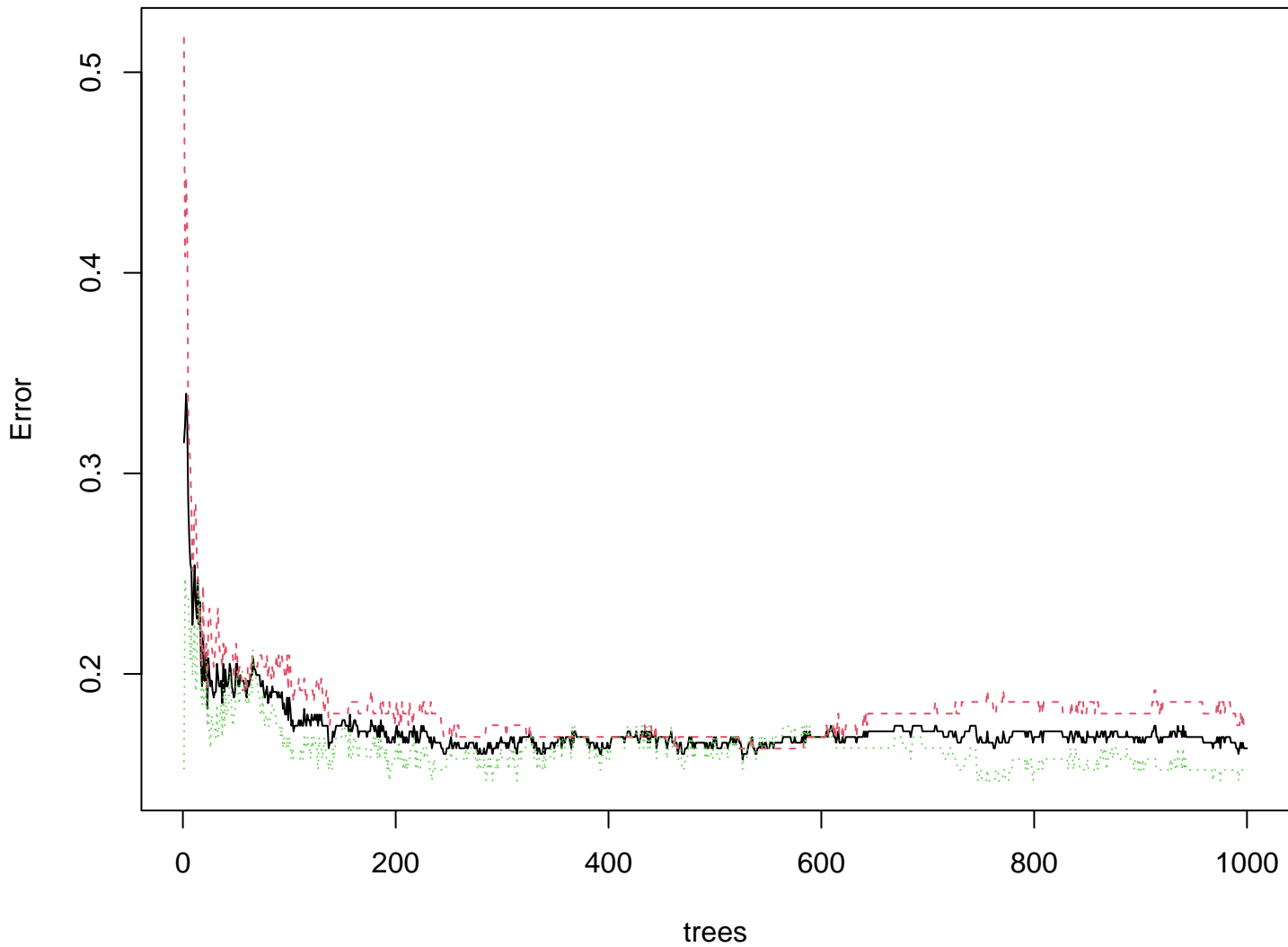

Supplement: Supplementary file 1 — Supporting information. [file IID3-13-e70166-s001.zip › Supplementary materials/S4-Machine learning/RF/Rplot.pdf]

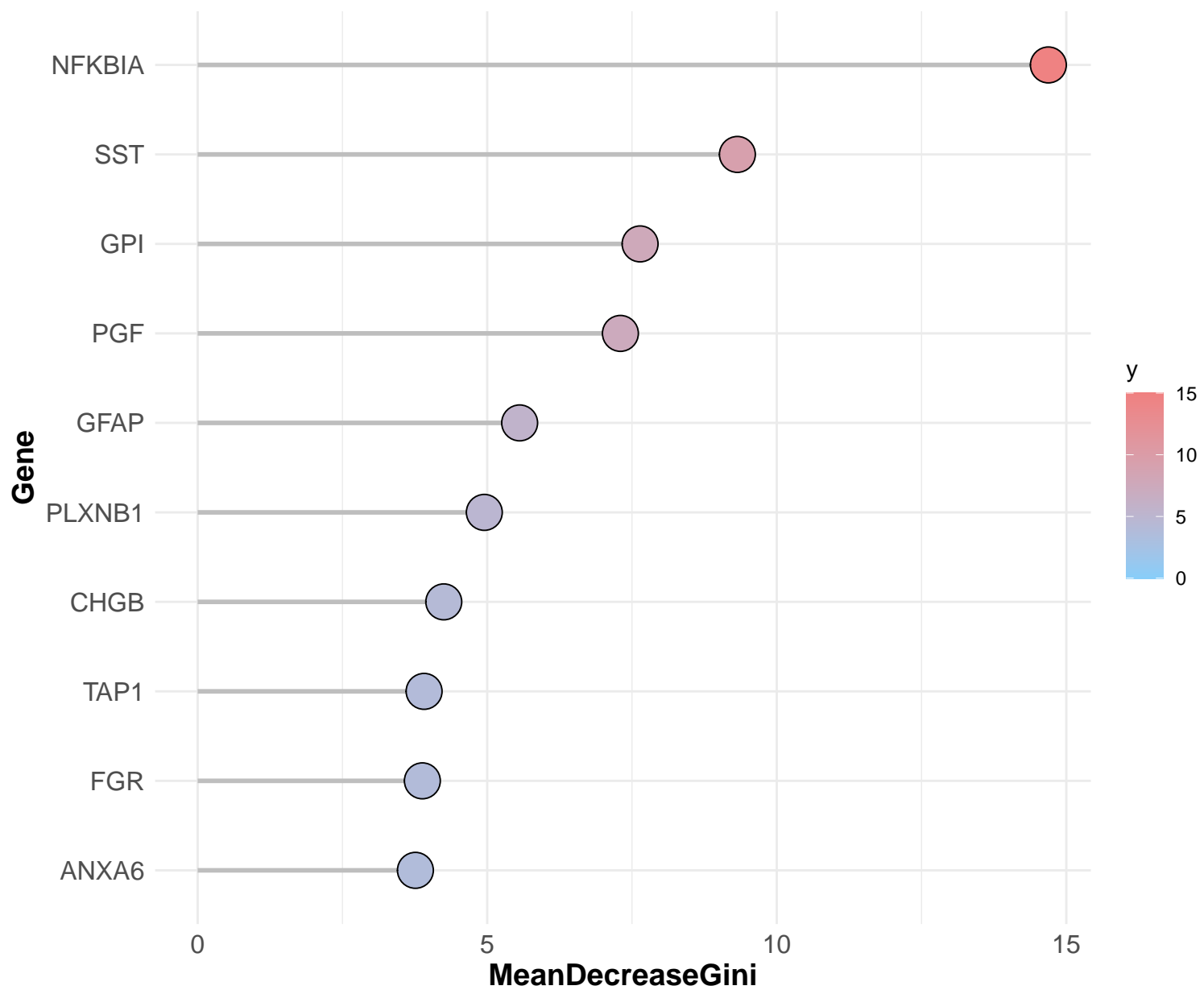

Supplement: Supplementary file 1 — Supporting information. [file IID3-13-e70166-s001.zip › Supplementary materials/S4-Machine learning/RF/Rplot05.pdf]

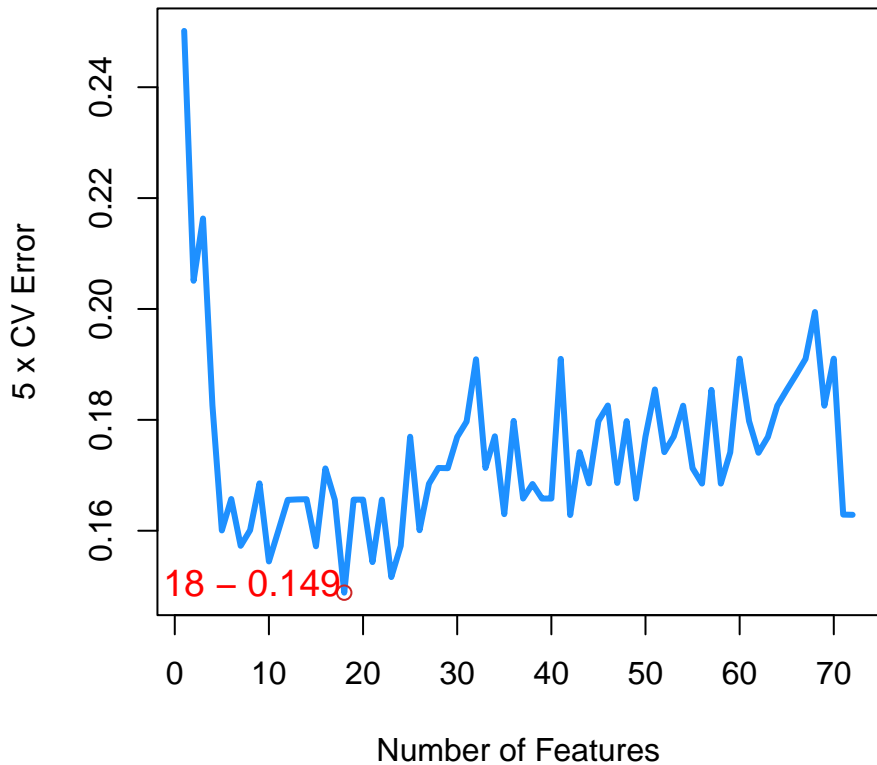

Supplement: Supplementary file 1 — Supporting information. [file IID3-13-e70166-s001.zip › Supplementary materials/S4-Machine learning/SVM/B_svm-error.pdf]

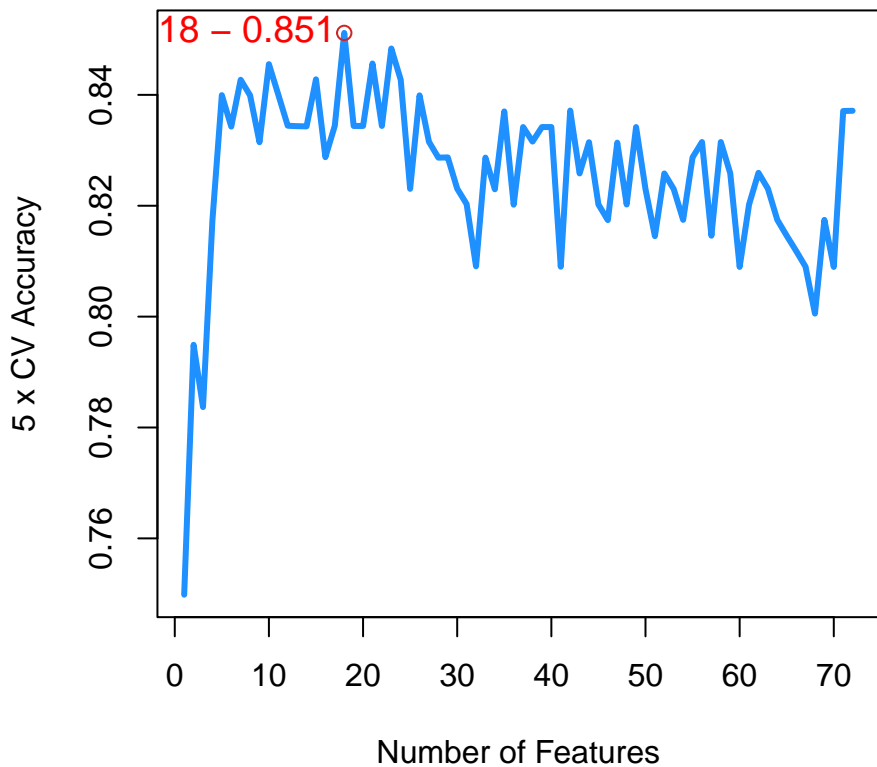

Supplement: Supplementary file 1 — Supporting information. [file IID3-13-e70166-s001.zip › Supplementary materials/S4-Machine learning/SVM/svm-accuracy.pdf]

# Feature importance

Features

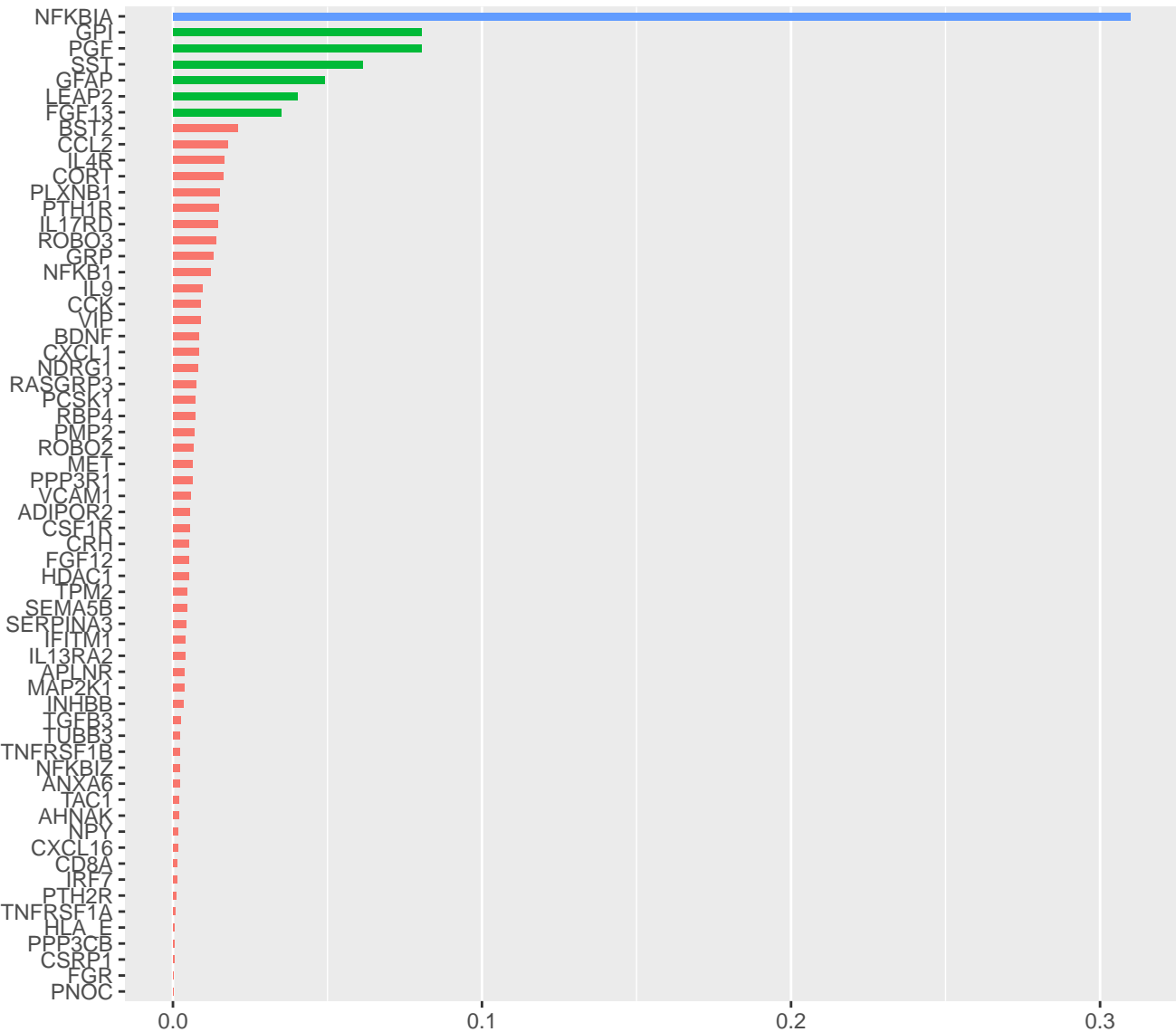

Cluster

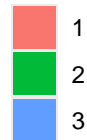

Importance

Supplement: Supplementary file 1 — Supporting information. [file IID3-13-e70166-s001.zip › Supplementary materials/S4-Machine learning/xgboost/boost.pdf]

# Feature importance

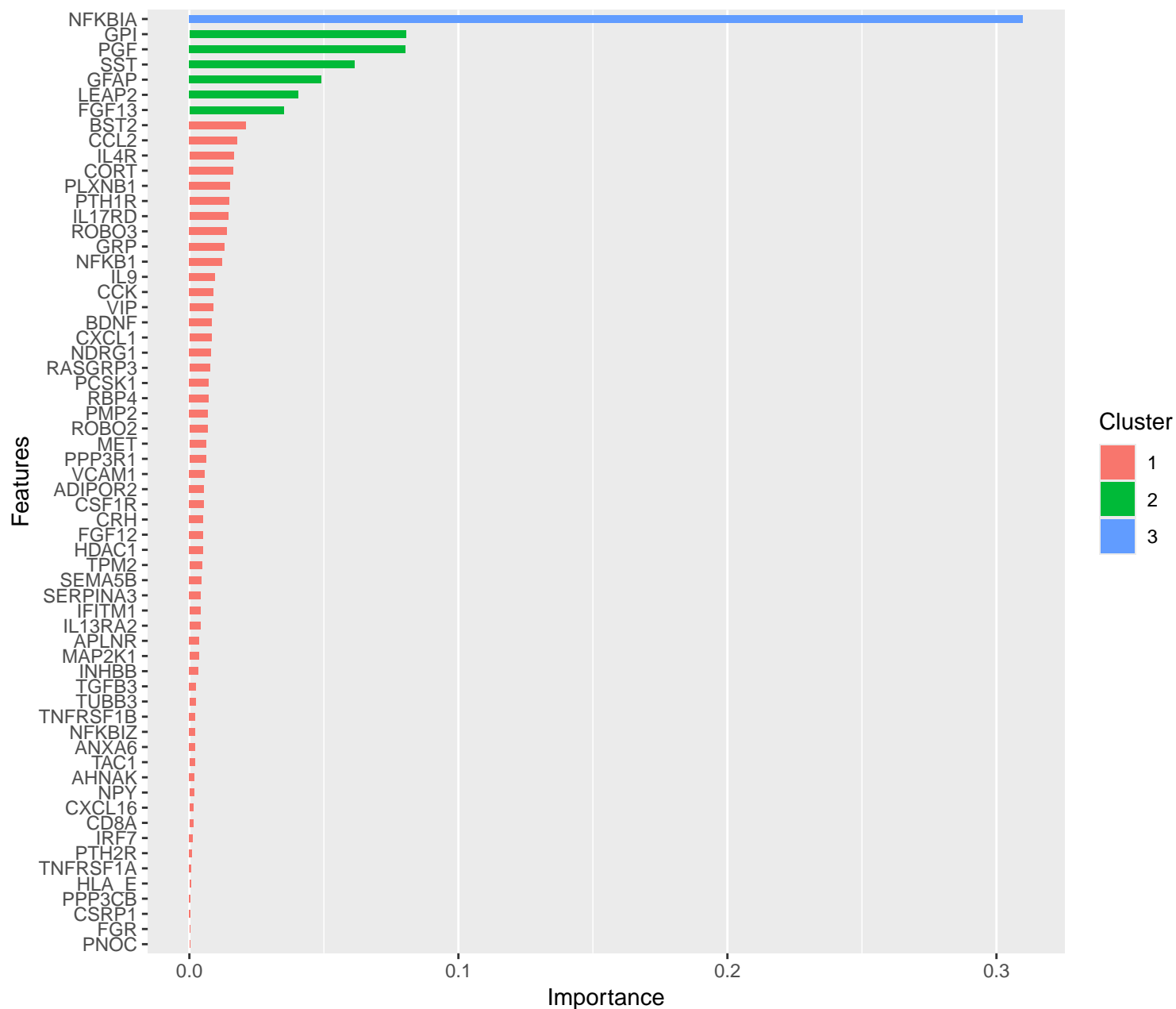

Supplement: Supplementary file 1 — Supporting information. [file IID3-13-e70166-s001.zip › Supplementary materials/S4-Machine learning/xgboost/Rplot.pdf]

Type AD Con

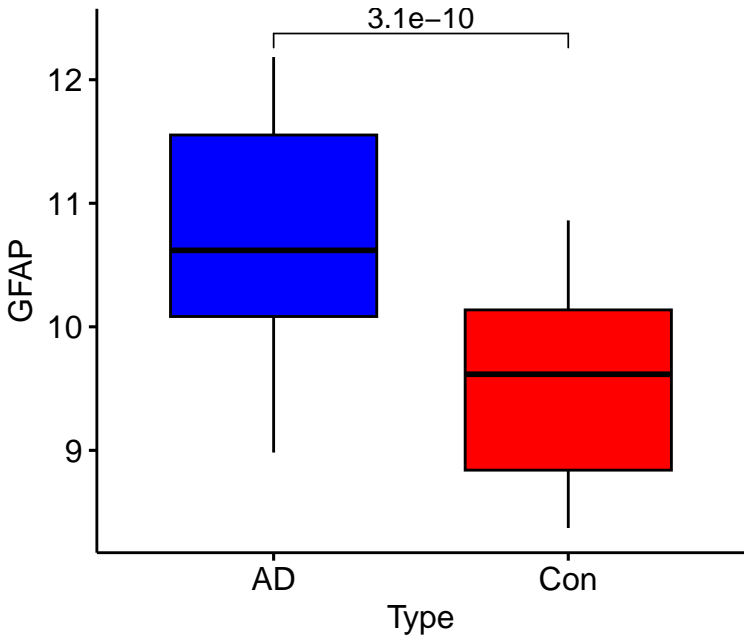

Supplement: Supplementary file 1 — Supporting information. [file IID3-13-e70166-s001.zip › Supplementary materials/S5-Clinical correlation curves and Key gene expression/test/GFAP_boxplotdiff.pdf]

# GFAP

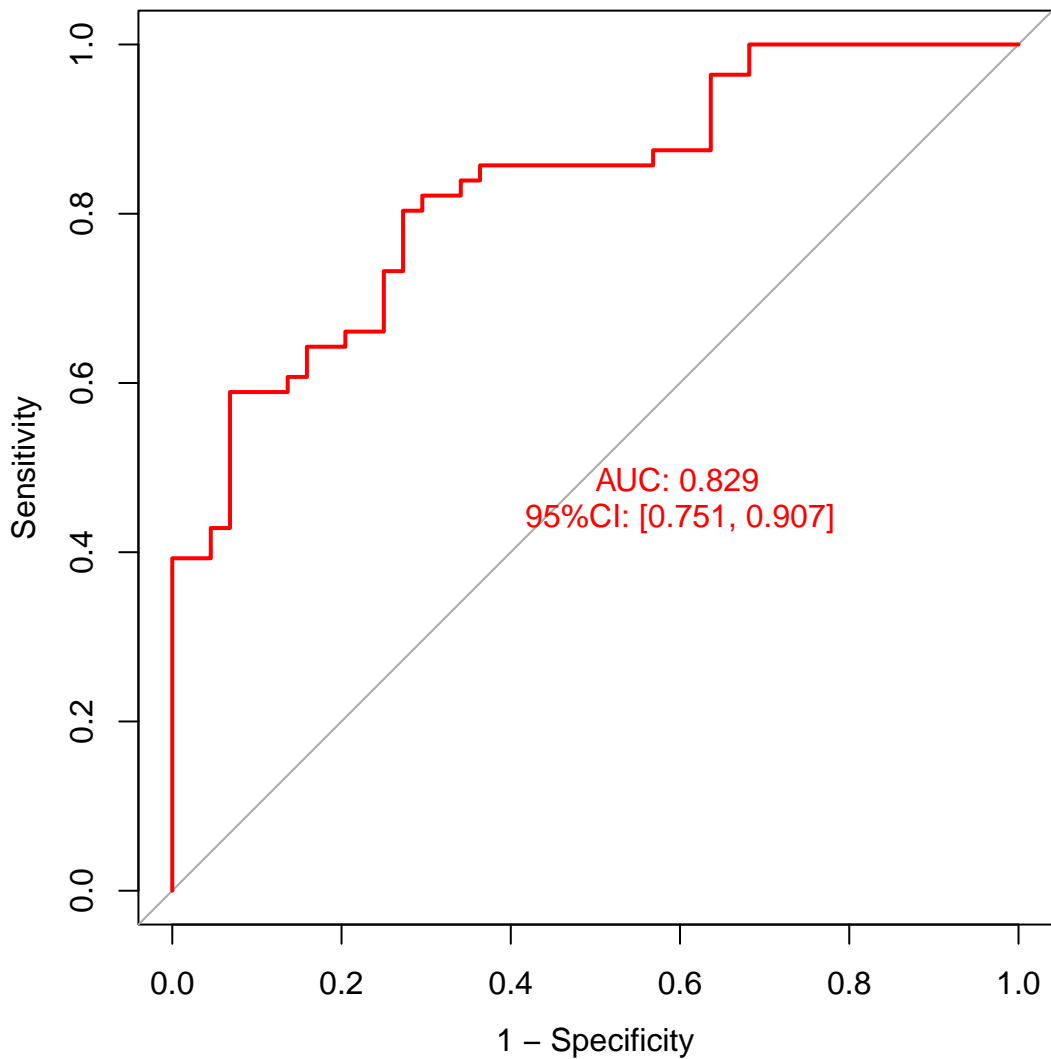

Supplement: Supplementary file 1 — Supporting information. [file IID3-13-e70166-s001.zip › Supplementary materials/S5-Clinical correlation curves and Key gene expression/test/GFAP_ROC.pdf]

Type 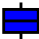 AD 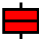 Con

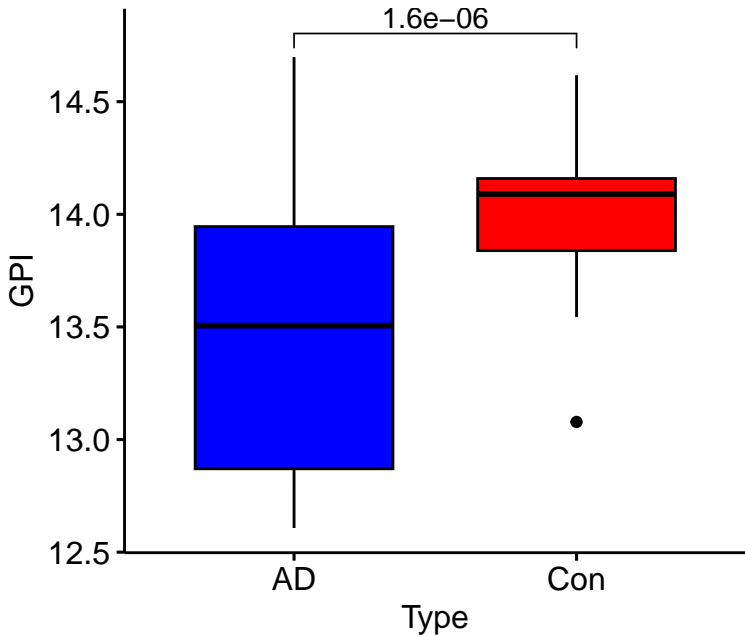

Supplement: Supplementary file 1 — Supporting information. [file IID3-13-e70166-s001.zip › Supplementary materials/S5-Clinical correlation curves and Key gene expression/test/GPI_boxplotdiff.pdf]

# GPI

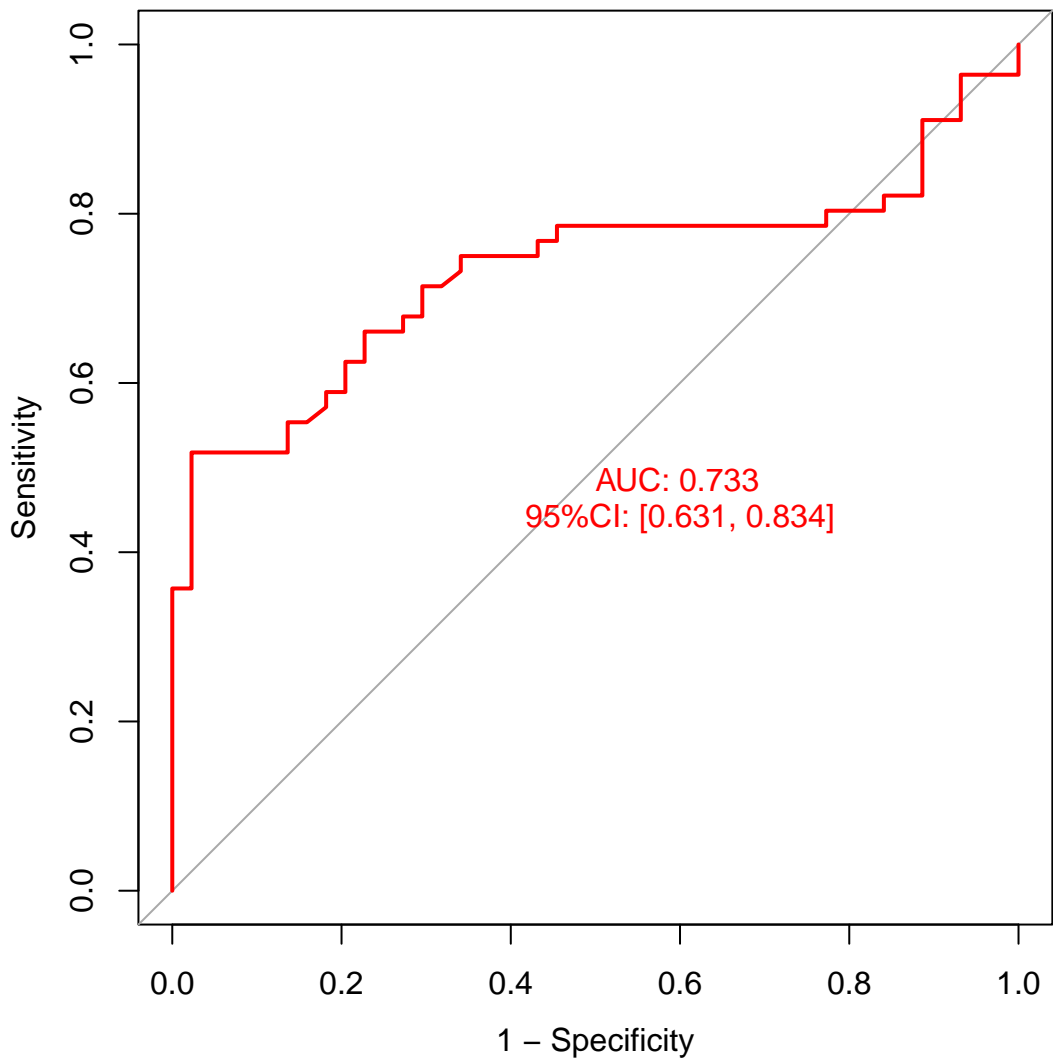

Supplement: Supplementary file 1 — Supporting information. [file IID3-13-e70166-s001.zip › Supplementary materials/S5-Clinical correlation curves and Key gene expression/test/GPI_ROC.pdf]

Type AD Con

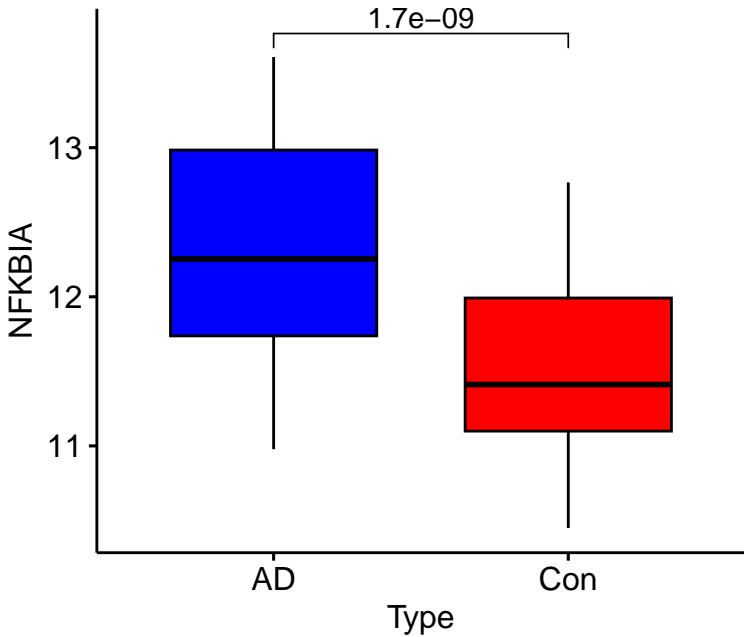

Supplement: Supplementary file 1 — Supporting information. [file IID3-13-e70166-s001.zip › Supplementary materials/S5-Clinical correlation curves and Key gene expression/test/NFKBIA_boxplotdiff.pdf]

# NFKBIA

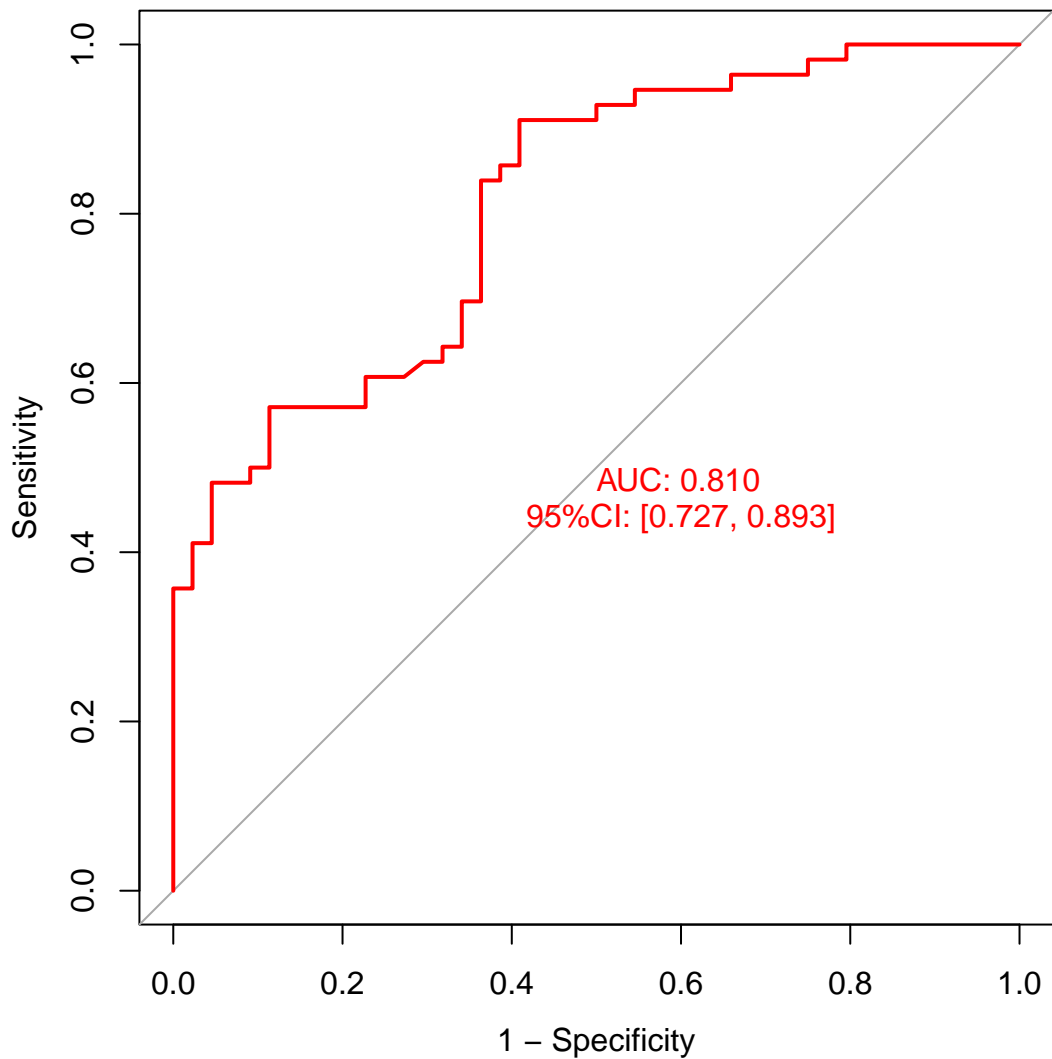

Supplement: Supplementary file 1 — Supporting information. [file IID3-13-e70166-s001.zip › Supplementary materials/S5-Clinical correlation curves and Key gene expression/test/NFKBIA_ROC.pdf]

Points

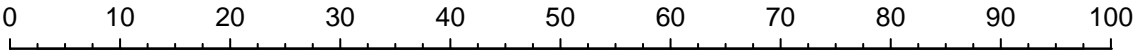

NFKBIA

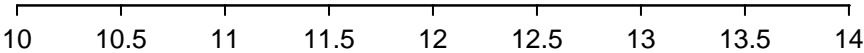

GFAP

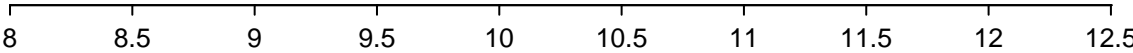

SST

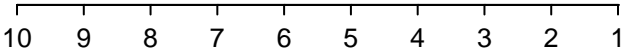

GPI

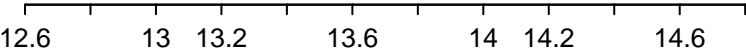

PGF

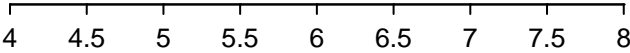

Total Points

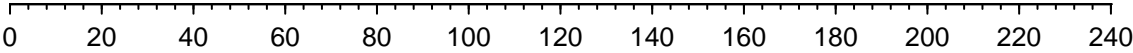

Linear Predictor

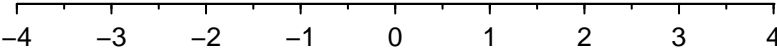

Supplement: Supplementary file 1 — Supporting information. [file IID3-13-e70166-s001.zip › Supplementary materials/S5-Clinical correlation curves and Key gene expression/test/nomogram.pdf]

Type 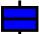 AD 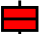 Con

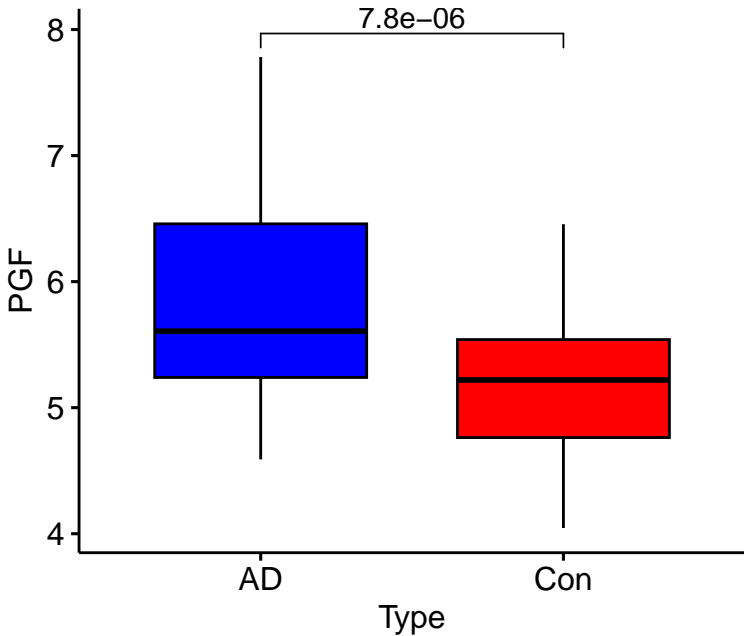

Supplement: Supplementary file 1 — Supporting information. [file IID3-13-e70166-s001.zip › Supplementary materials/S5-Clinical correlation curves and Key gene expression/test/PGF_boxplotdiff.pdf]

# PGF

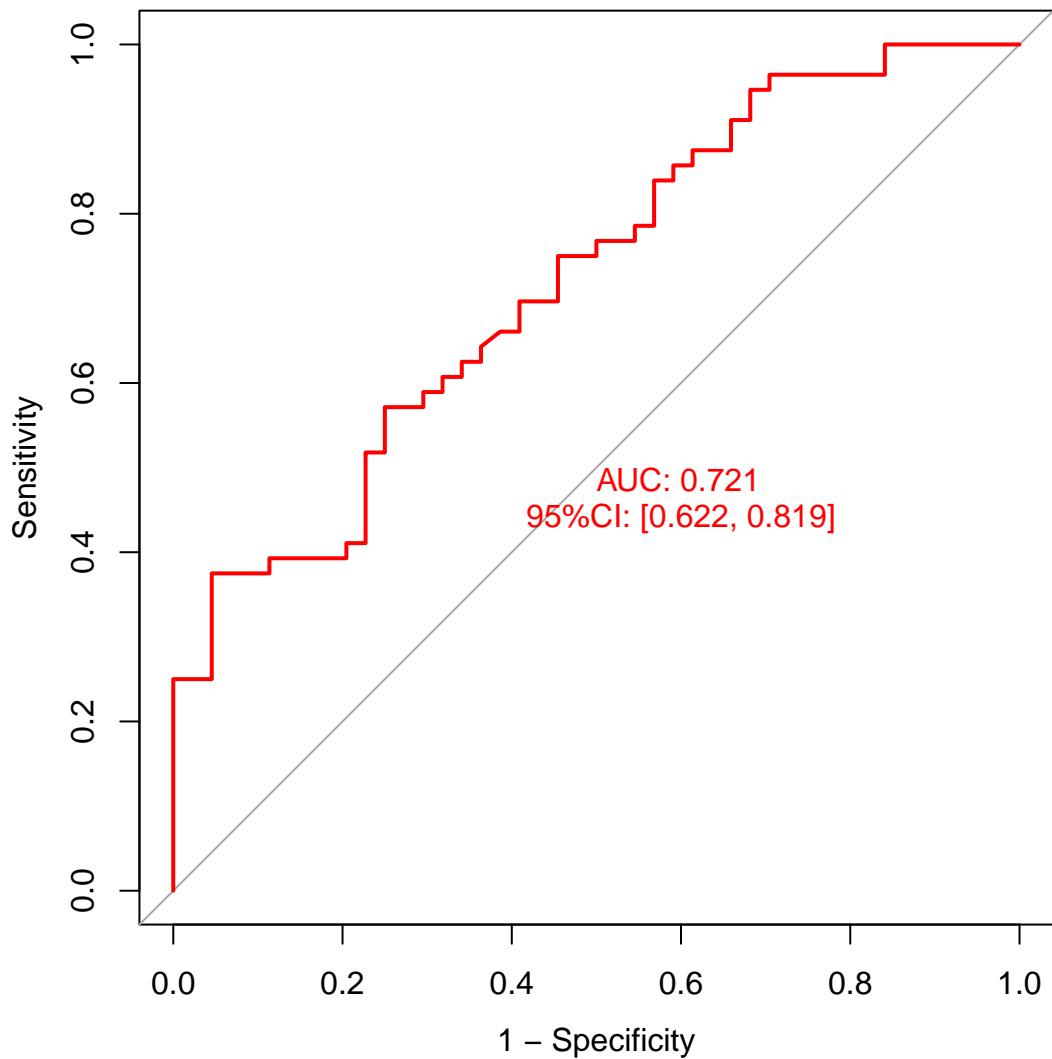

Supplement: Supplementary file 1 — Supporting information. [file IID3-13-e70166-s001.zip › Supplementary materials/S5-Clinical correlation curves and Key gene expression/test/PGF_ROC.pdf]

Type 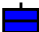 AD 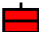 Con

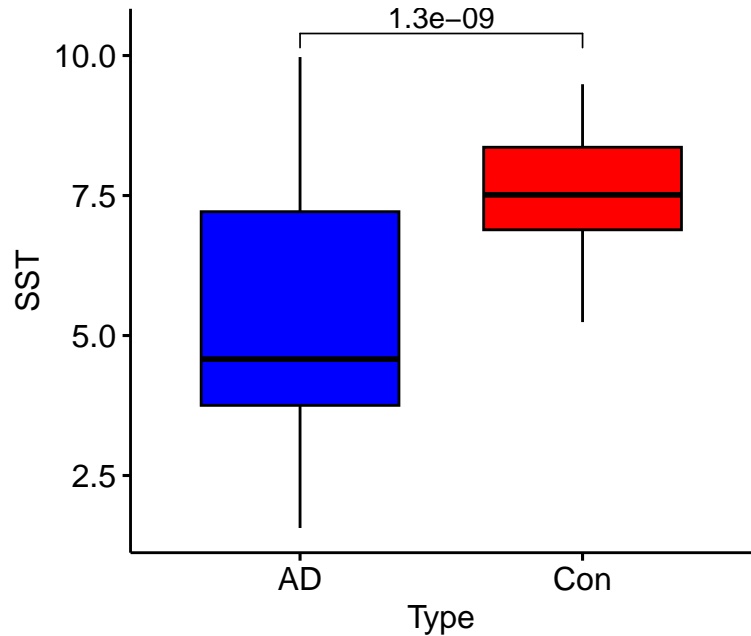

Supplement: Supplementary file 1 — Supporting information. [file IID3-13-e70166-s001.zip › Supplementary materials/S5-Clinical correlation curves and Key gene expression/test/SST_boxplotdiff.pdf]

# SST

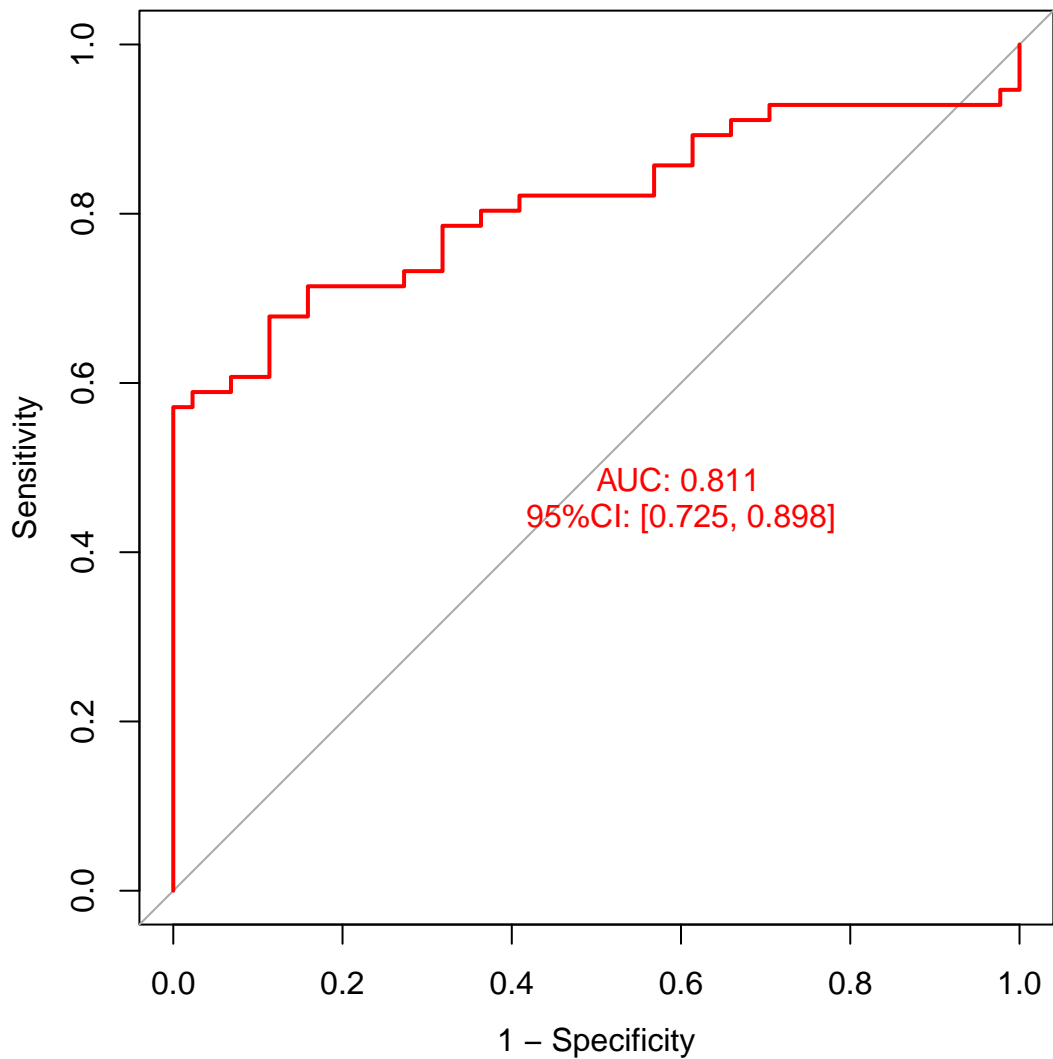

Supplement: Supplementary file 1 — Supporting information. [file IID3-13-e70166-s001.zip › Supplementary materials/S5-Clinical correlation curves and Key gene expression/test/SST_ROC.pdf]

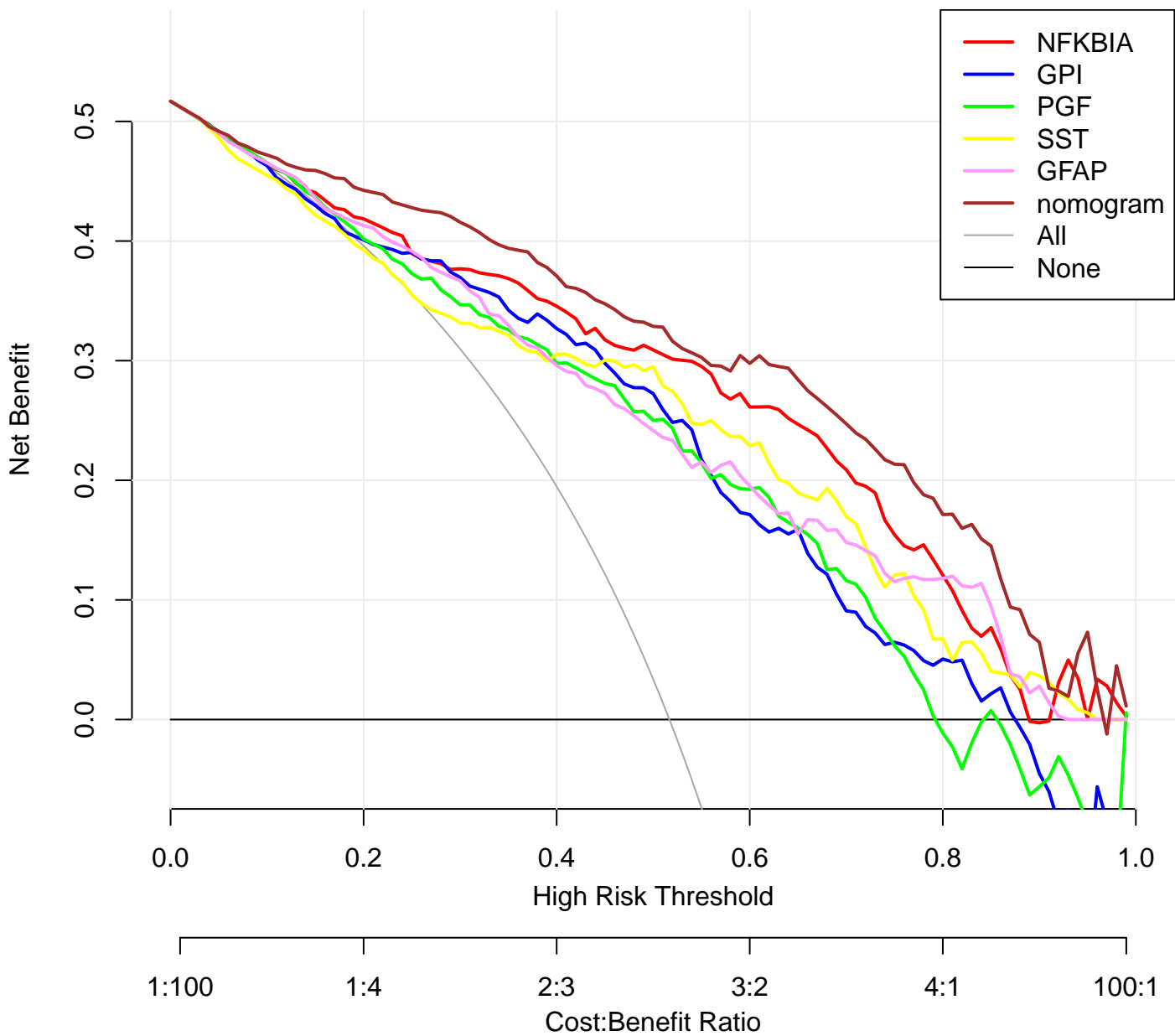

Supplement: Supplementary file 1 — Supporting information. [file IID3-13-e70166-s001.zip › Supplementary materials/S5-Clinical correlation curves and Key gene expression/train/clinical decision curve.pdf]

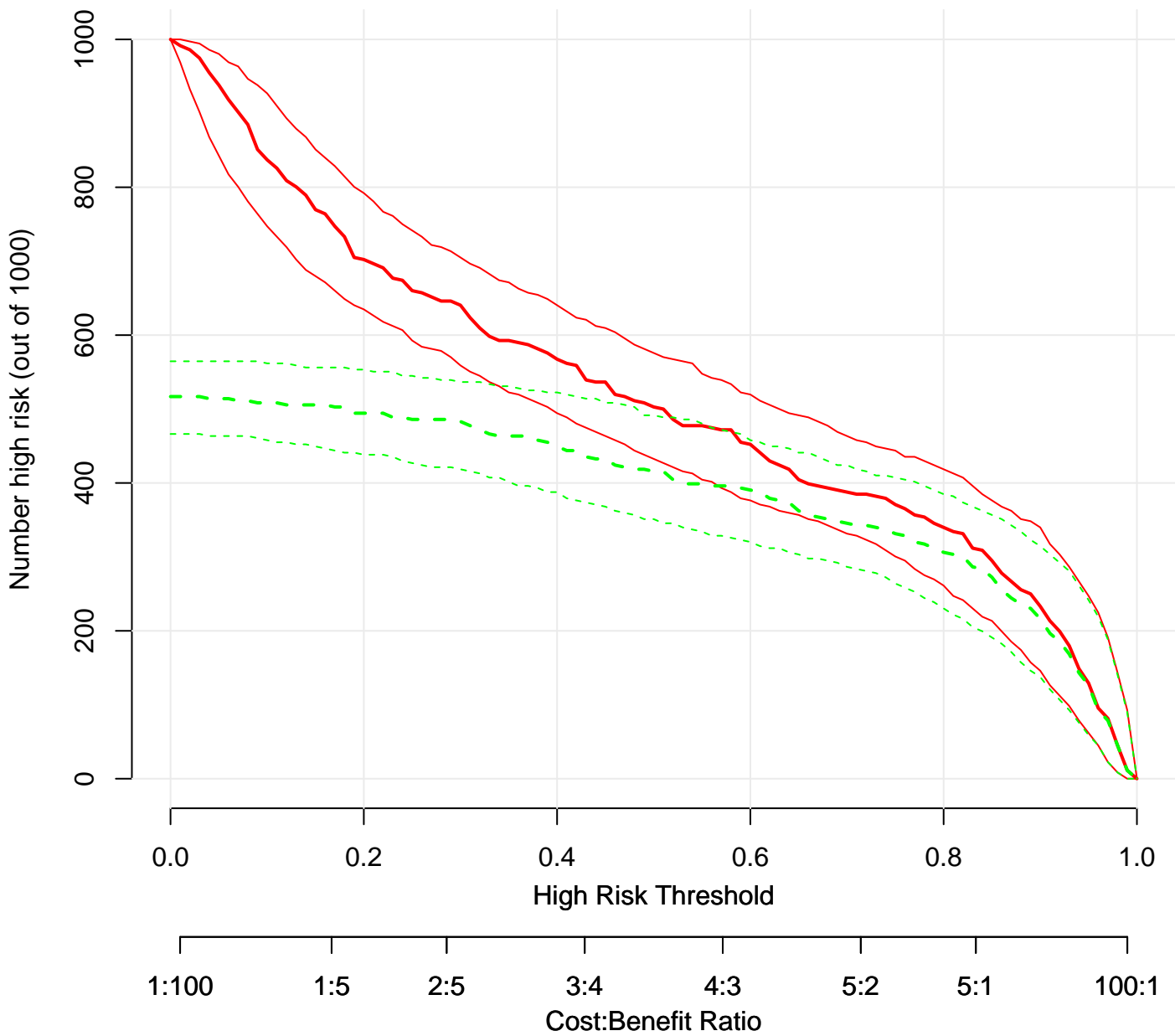

Supplement: Supplementary file 1 — Supporting information. [file IID3-13-e70166-s001.zip › Supplementary materials/S5-Clinical correlation curves and Key gene expression/train/clinical impact curve.pdf]

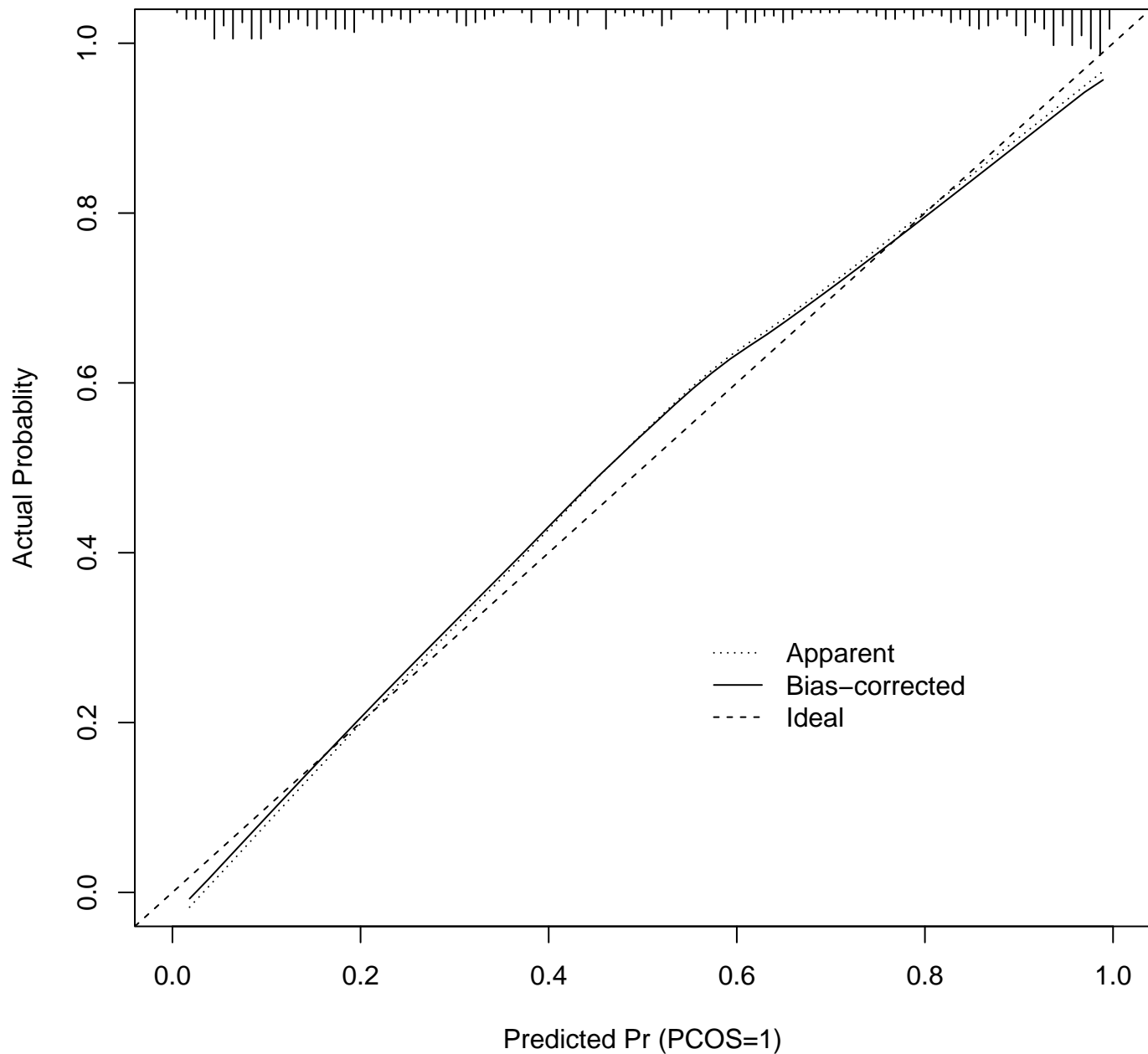

B= 1000 repetitions, boot

Mean absolute error=0.019 n=356

Supplement: Supplementary file 1 — Supporting information. [file IID3-13-e70166-s001.zip › Supplementary materials/S5-Clinical correlation curves and Key gene expression/train/correction curve.pdf]

Type C P

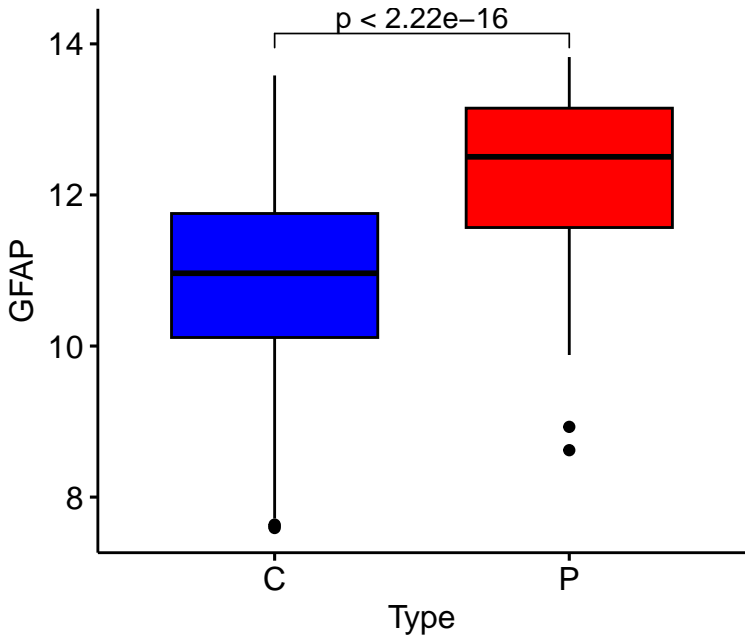

Supplement: Supplementary file 1 — Supporting information. [file IID3-13-e70166-s001.zip › Supplementary materials/S5-Clinical correlation curves and Key gene expression/train/GFAP_boxplotdiff.pdf]

# GFAP

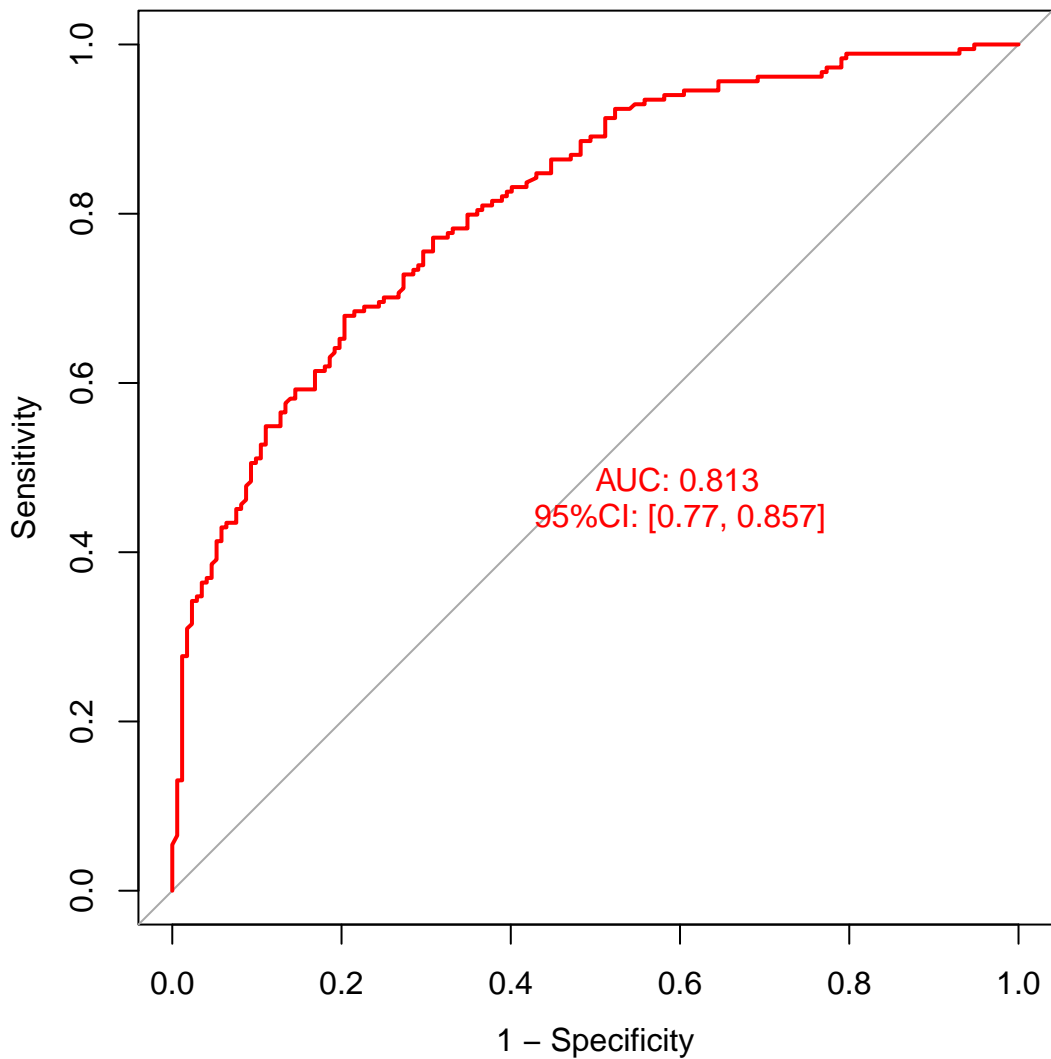

Supplement: Supplementary file 1 — Supporting information. [file IID3-13-e70166-s001.zip › Supplementary materials/S5-Clinical correlation curves and Key gene expression/train/GFAP_ROC.pdf]

Type C P

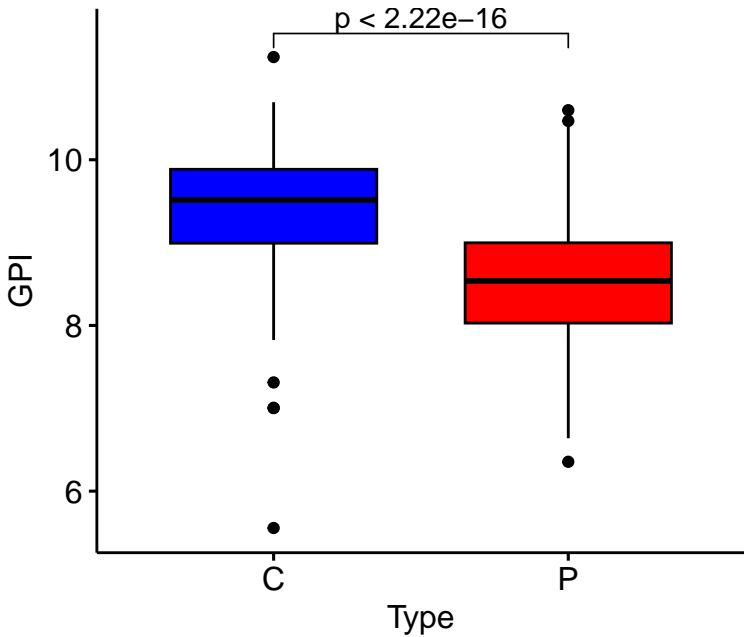

Supplement: Supplementary file 1 — Supporting information. [file IID3-13-e70166-s001.zip › Supplementary materials/S5-Clinical correlation curves and Key gene expression/train/GPI_boxplotdiff.pdf]

# GPI

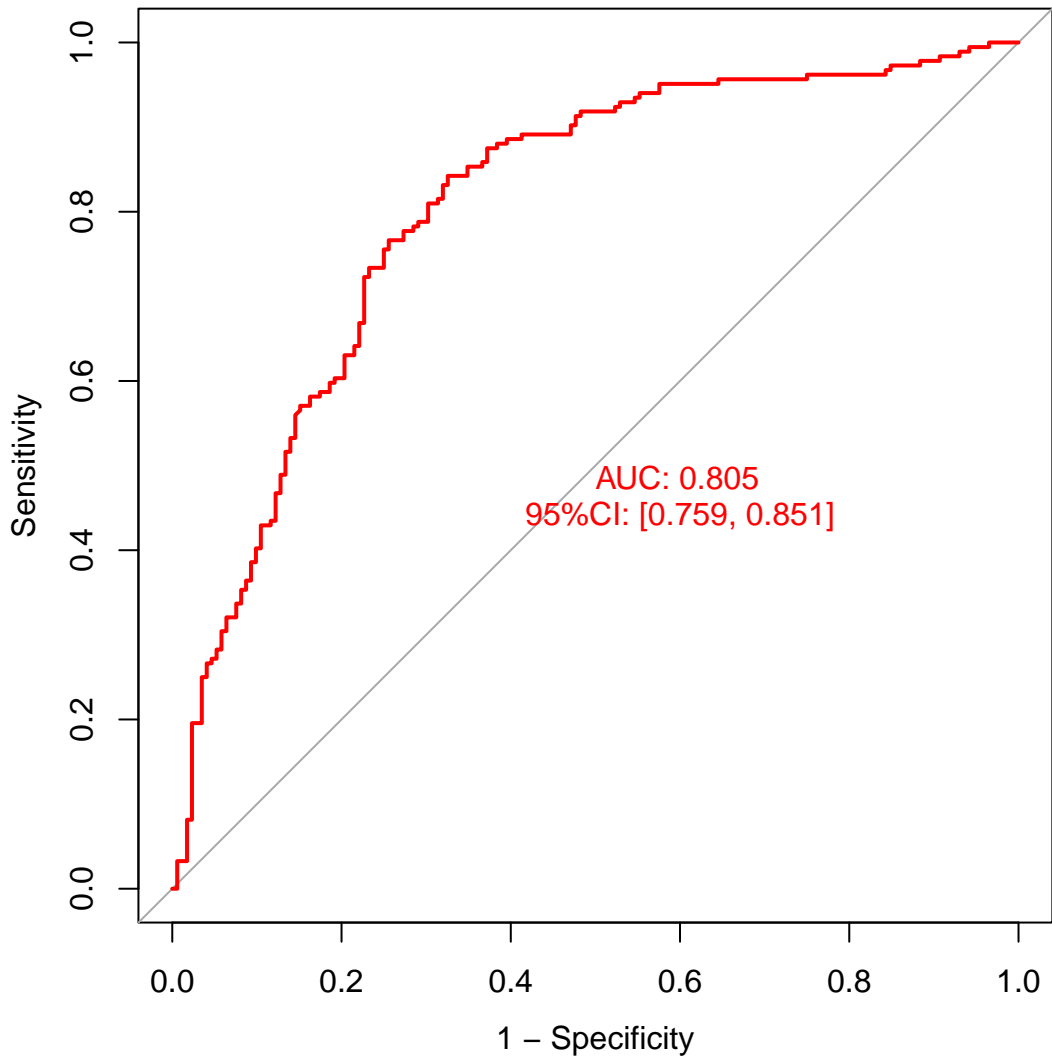

Supplement: Supplementary file 1 — Supporting information. [file IID3-13-e70166-s001.zip › Supplementary materials/S5-Clinical correlation curves and Key gene expression/train/GPI_ROC.pdf]

Type C P

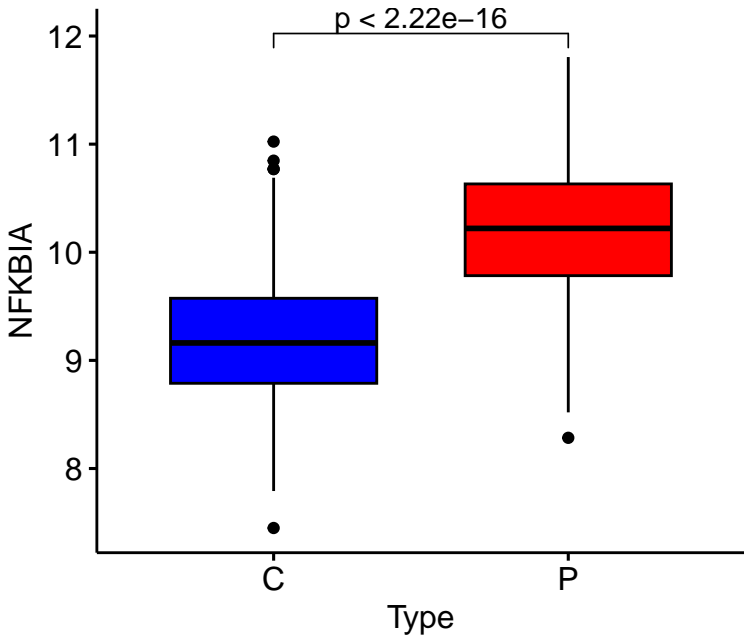

Supplement: Supplementary file 1 — Supporting information. [file IID3-13-e70166-s001.zip › Supplementary materials/S5-Clinical correlation curves and Key gene expression/train/NFKBIA_boxplotdiff.pdf]

# NFKBIA

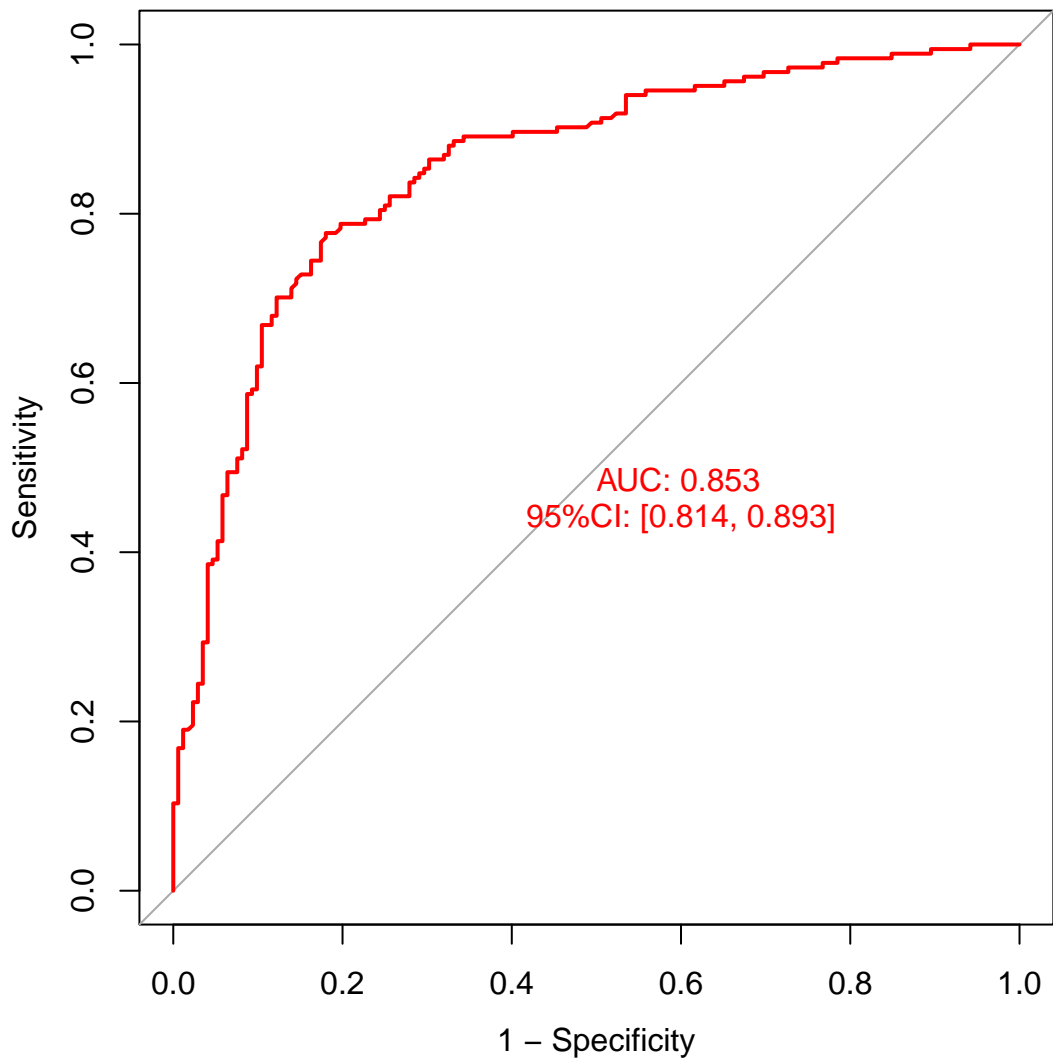

Supplement: Supplementary file 1 — Supporting information. [file IID3-13-e70166-s001.zip › Supplementary materials/S5-Clinical correlation curves and Key gene expression/train/NFKBIA_ROC.pdf]

# Nomogram

$\beta(X-m)$  terms

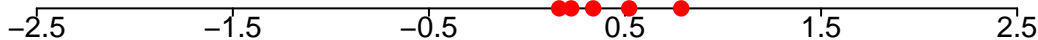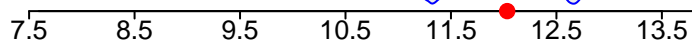

GFAP\*\*\*

SST

PGF

GPI\*\*

NFKBIA\*\*\*

Total score

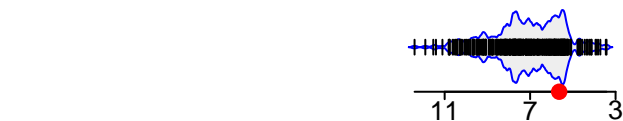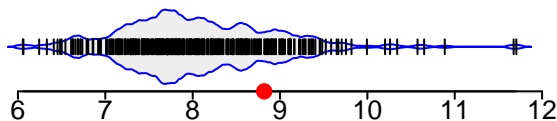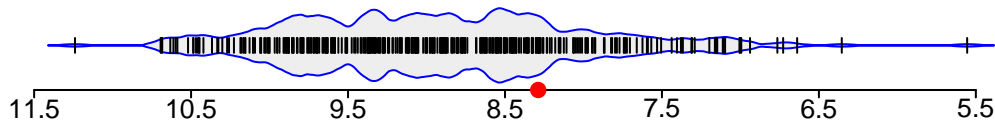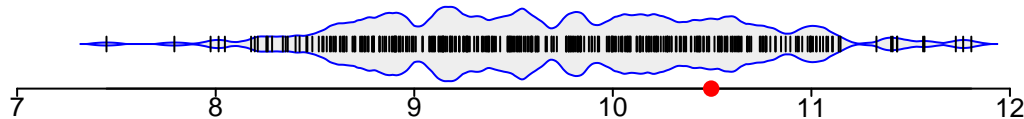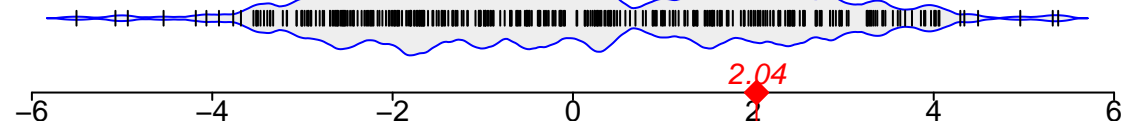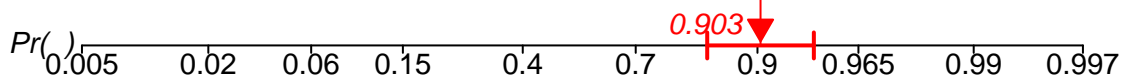

2.04

0.903

Supplement: Supplementary file 1 — Supporting information. [file IID3-13-e70166-s001.zip › Supplementary materials/S5-Clinical correlation curves and Key gene expression/train/Nomogram.pdf]

Type C P

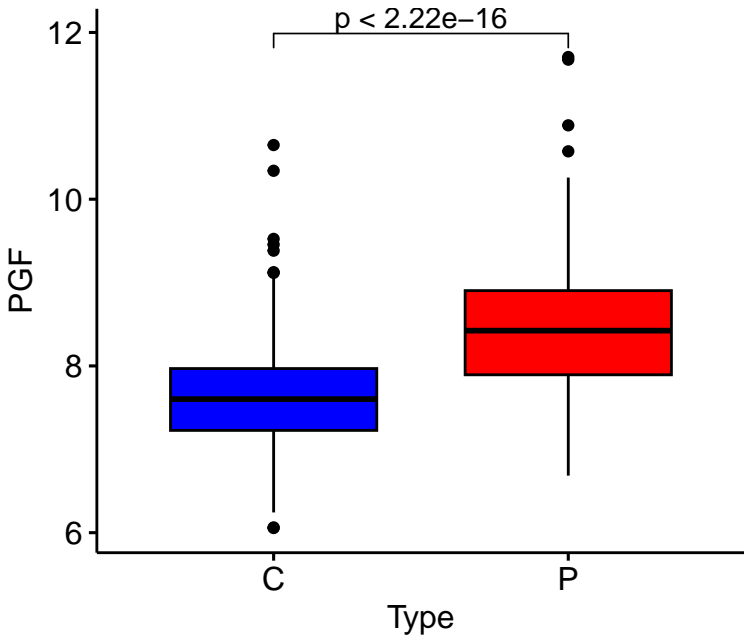

Supplement: Supplementary file 1 — Supporting information. [file IID3-13-e70166-s001.zip › Supplementary materials/S5-Clinical correlation curves and Key gene expression/train/PGF_boxplotdiff.pdf]

# PGF

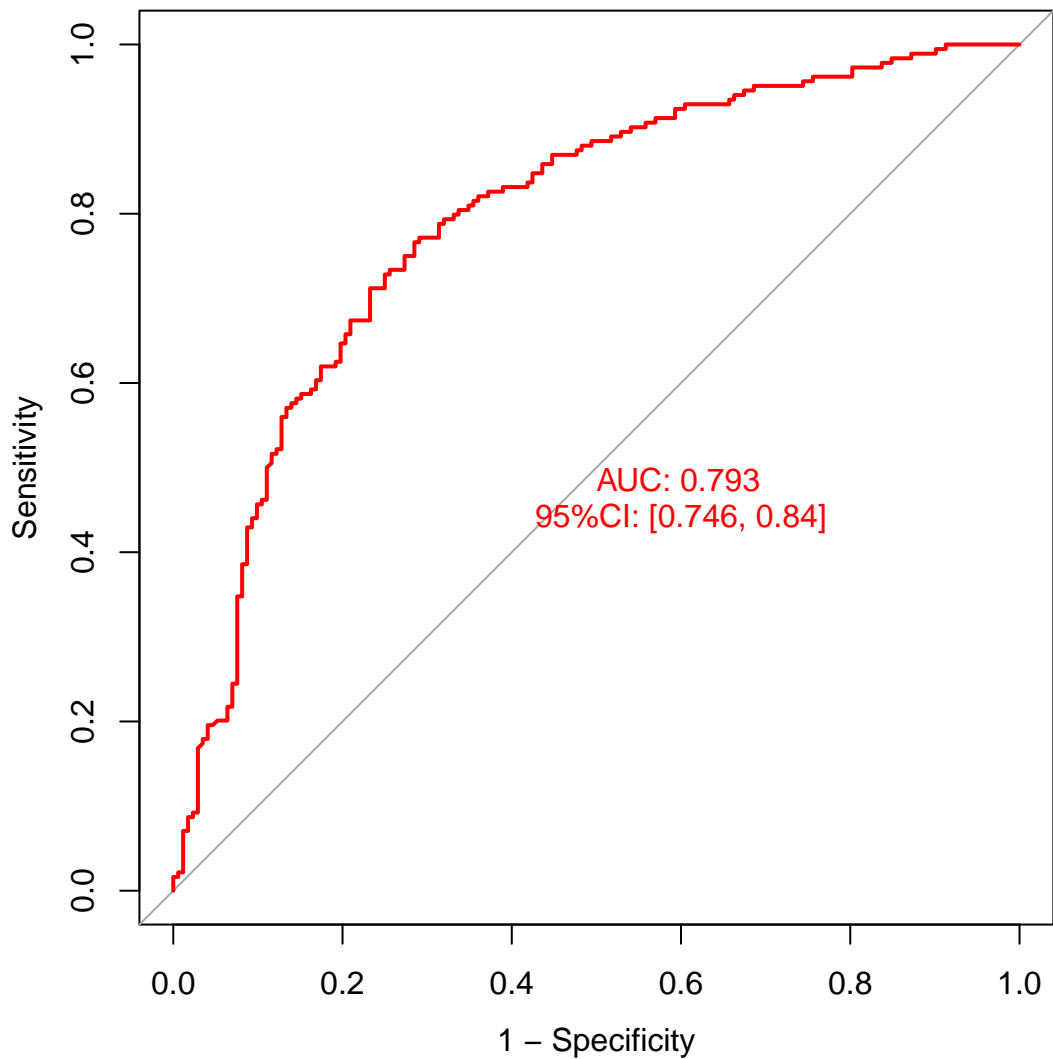

Supplement: Supplementary file 1 — Supporting information. [file IID3-13-e70166-s001.zip › Supplementary materials/S5-Clinical correlation curves and Key gene expression/train/PGF_ROC.pdf]

Type C P

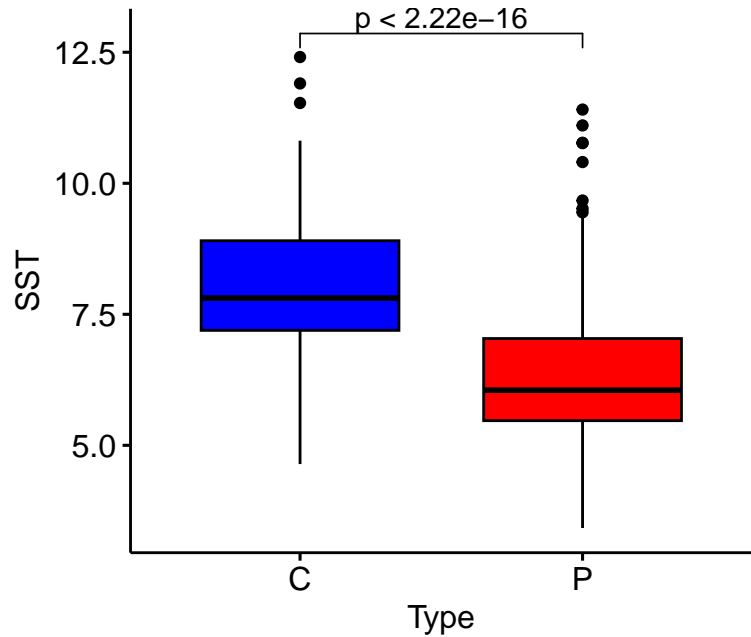

Supplement: Supplementary file 1 — Supporting information. [file IID3-13-e70166-s001.zip › Supplementary materials/S5-Clinical correlation curves and Key gene expression/train/SST_boxplotdiff.pdf]

# SST

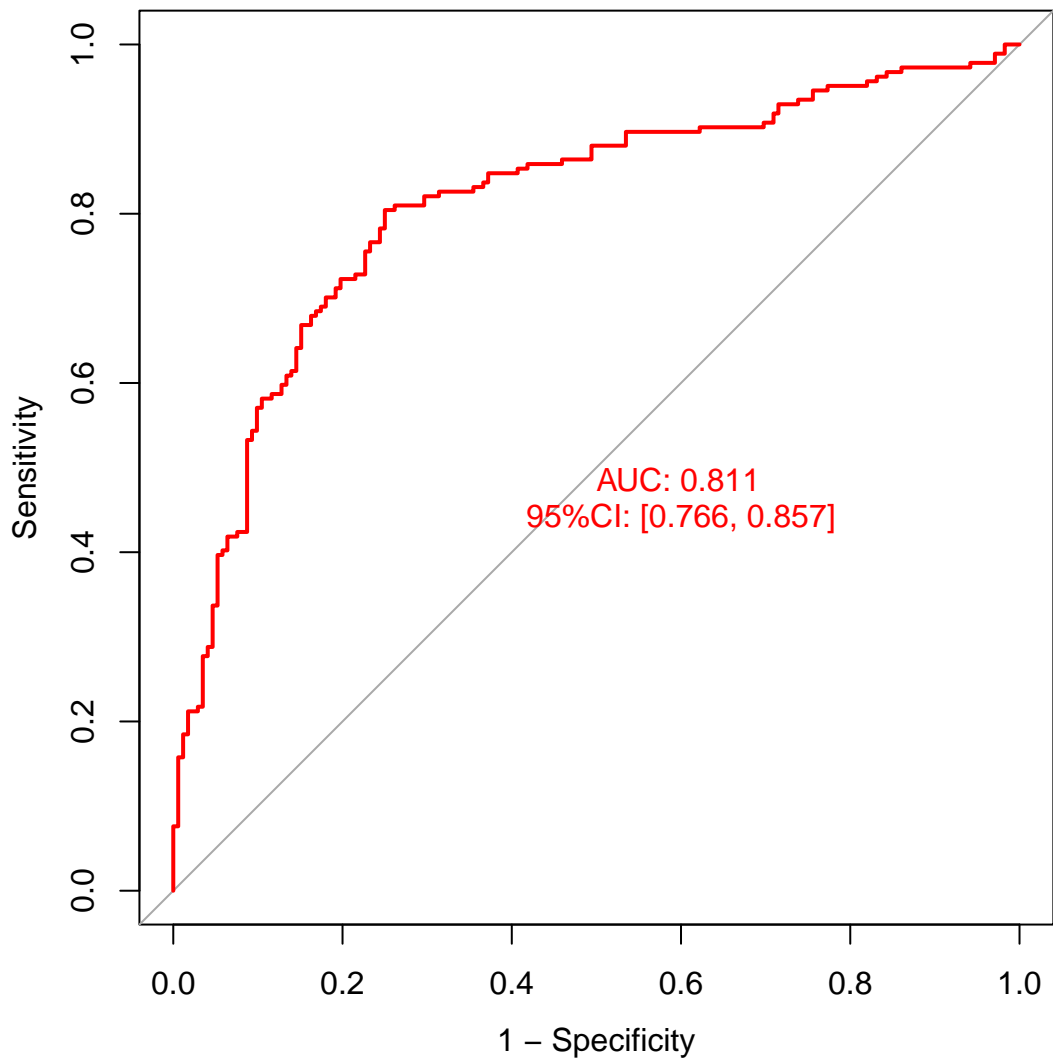

Supplement: Supplementary file 1 — Supporting information. [file IID3-13-e70166-s001.zip › Supplementary materials/S5-Clinical correlation curves and Key gene expression/train/SST_ROC.pdf]

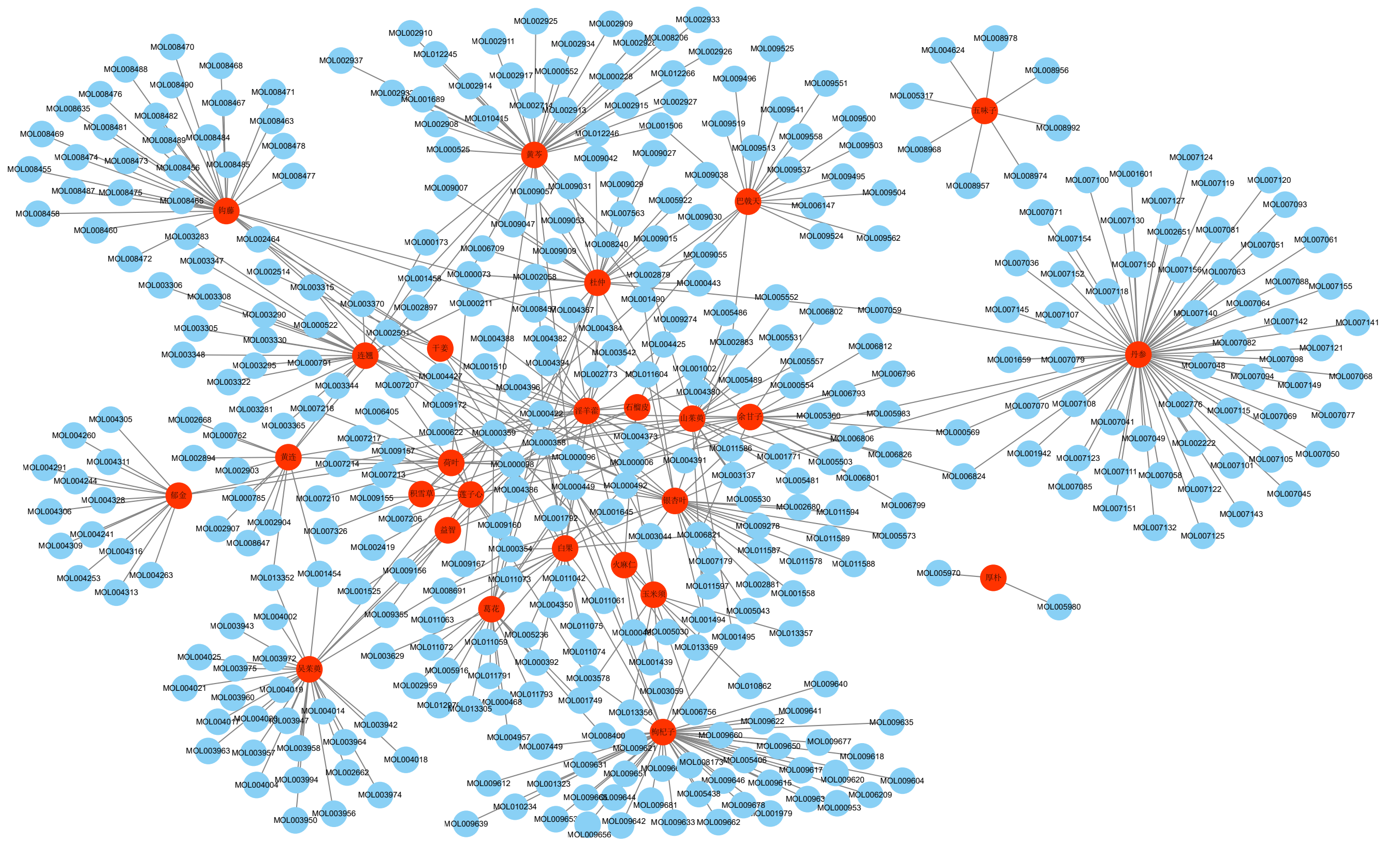

Supplement: Supplementary file 1 — Supporting information. [file IID3-13-e70166-s001.zip › Supplementary materials/S7-herb and ingredients/Sheet1.pdf]

Residue Cross Correlation

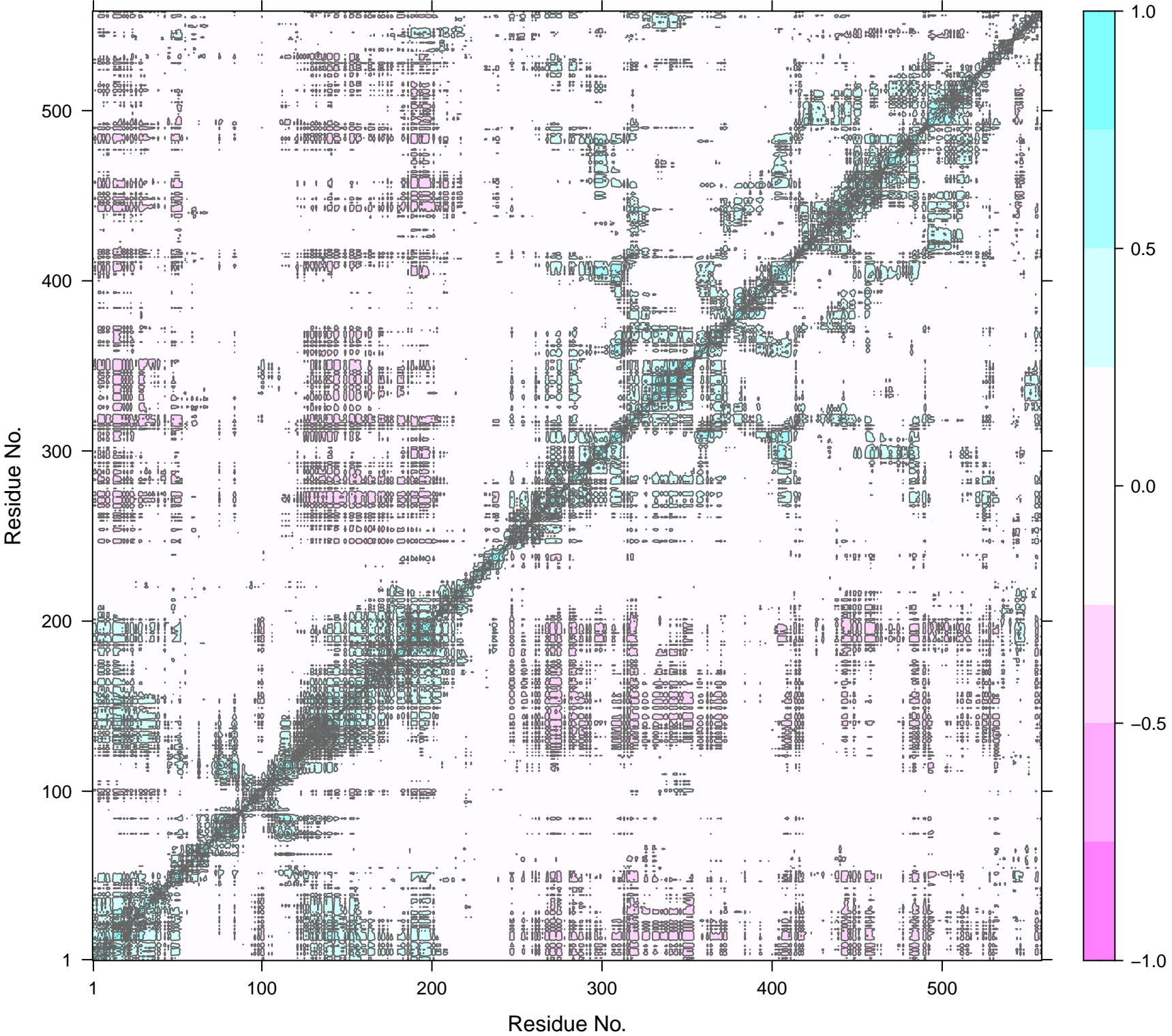

Supplement: Supplementary file 1 — Supporting information. [file IID3-13-e70166-s001.zip › Supplementary materials/S8-Molecular docking and kinetic simulation/Dynamic simulation/gpi-apodccp.pdf]

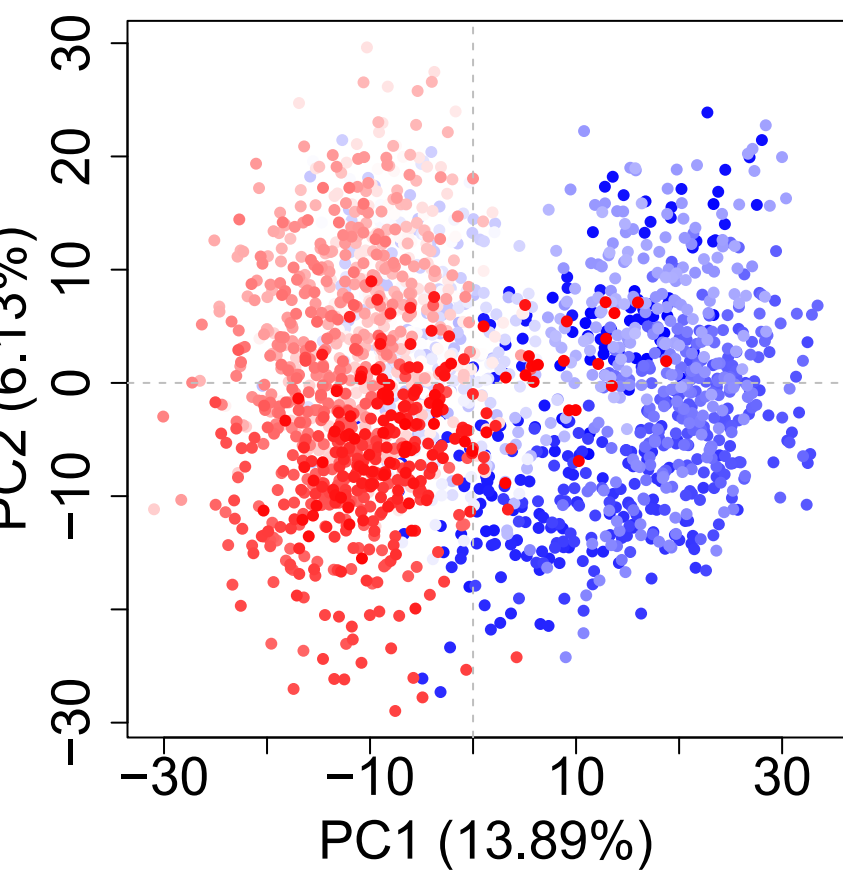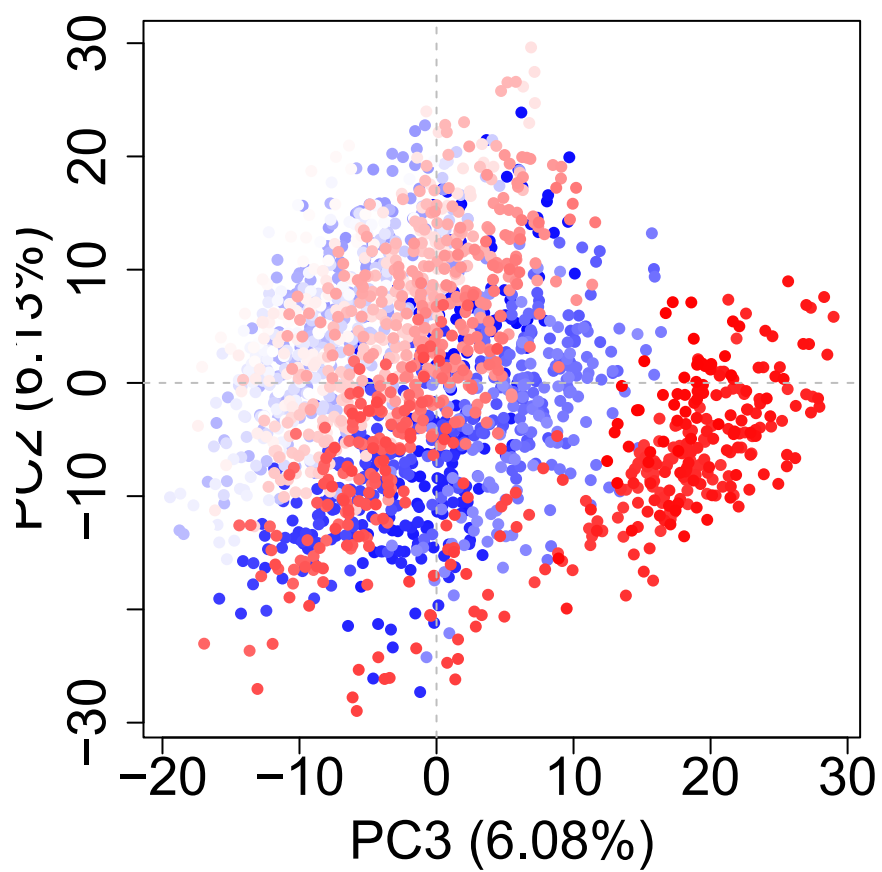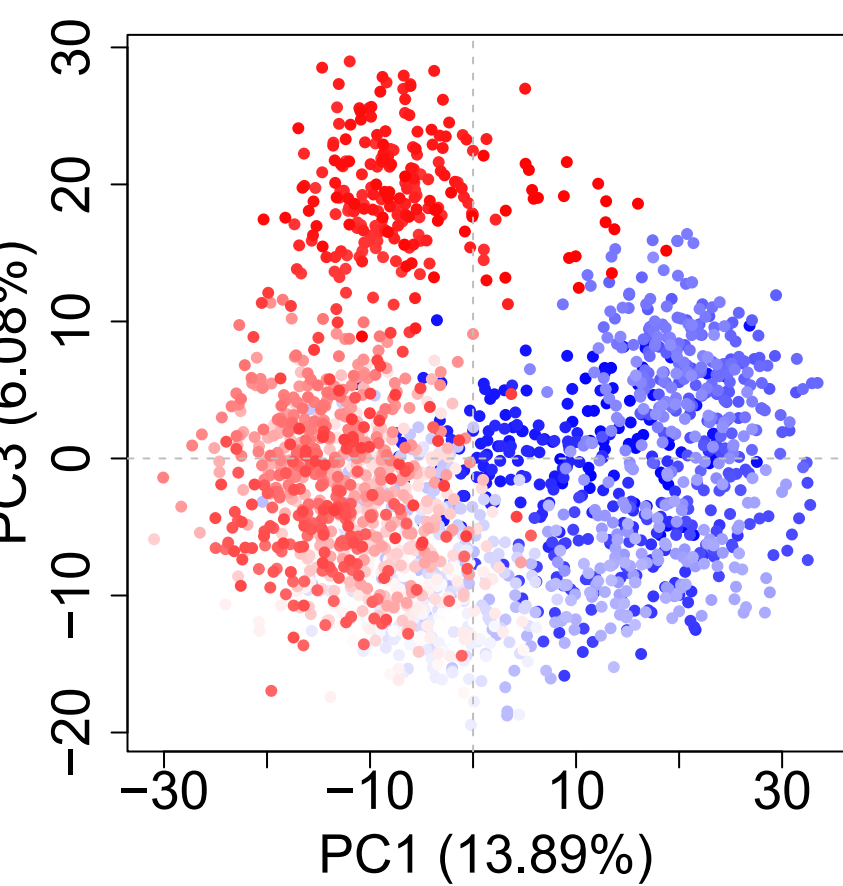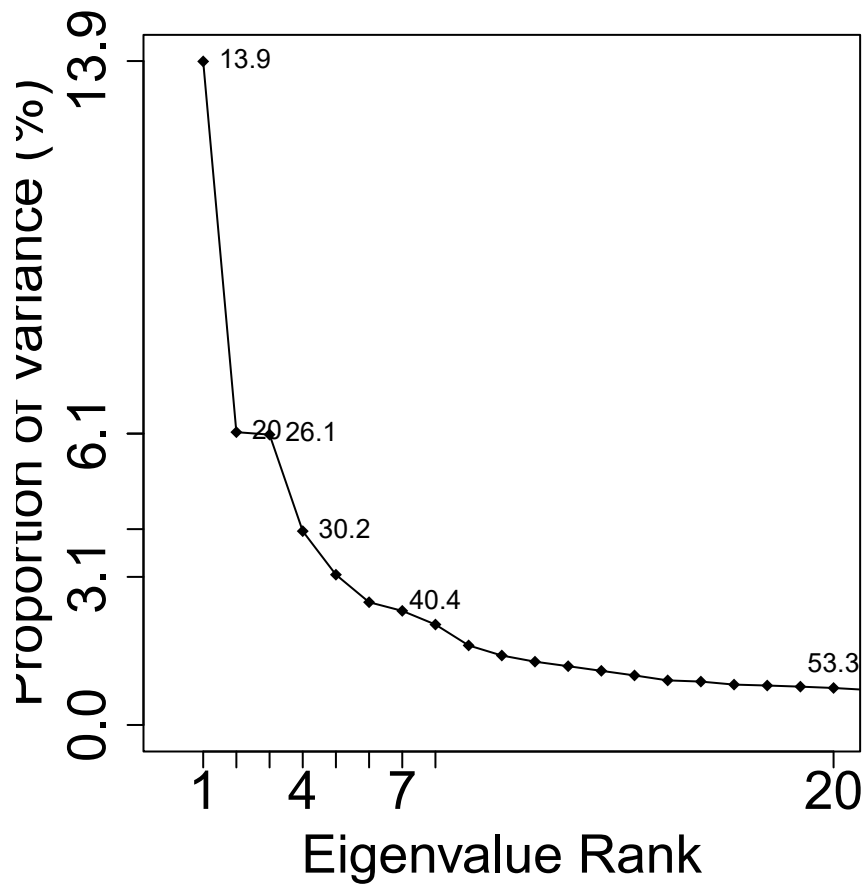

Supplement: Supplementary file 1 — Supporting information. [file IID3-13-e70166-s001.zip › Supplementary materials/S8-Molecular docking and kinetic simulation/Dynamic simulation/gpi-apopca.pdf]

Residue Cross Correlation

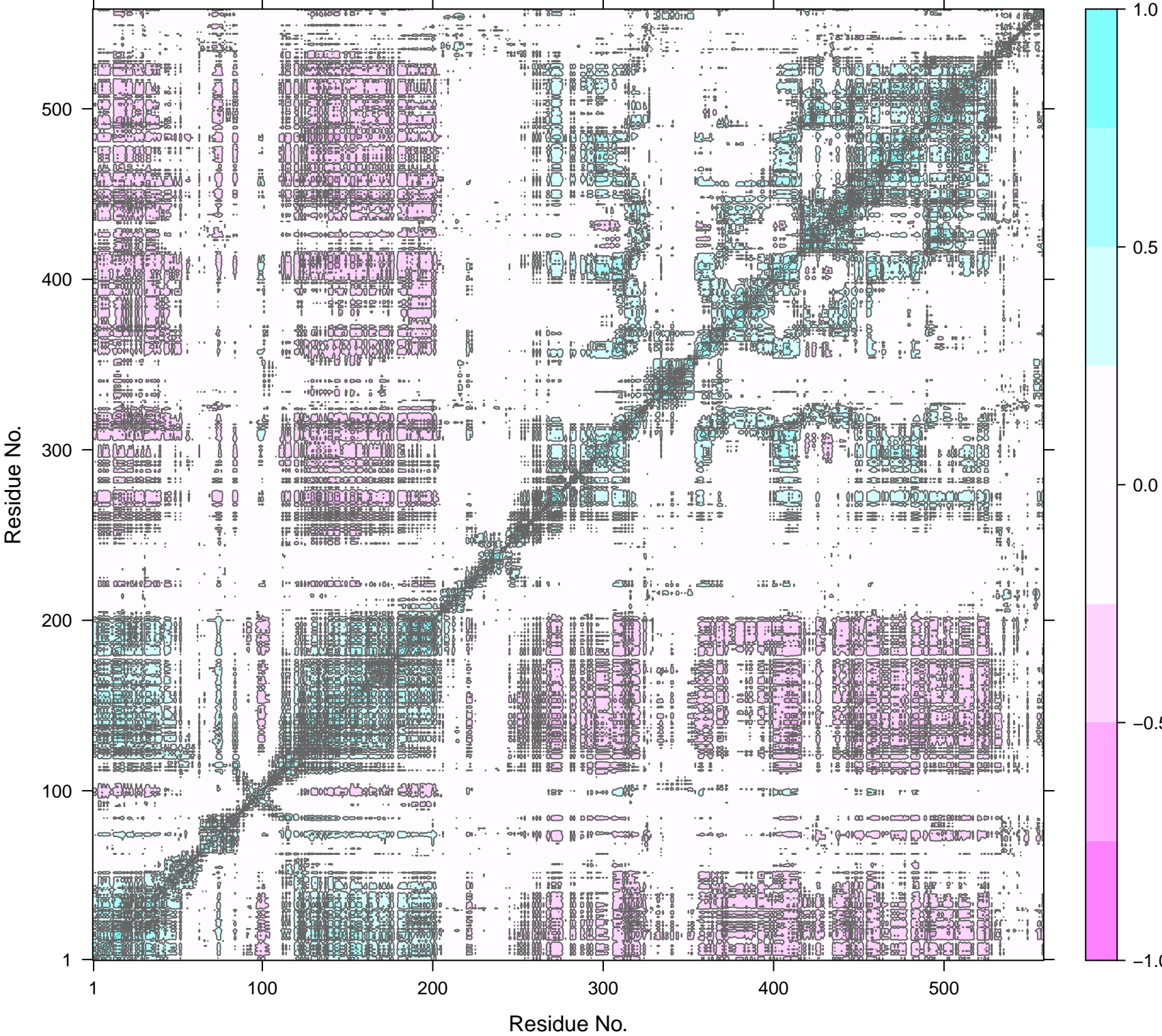

Supplement: Supplementary file 1 — Supporting information. [file IID3-13-e70166-s001.zip › Supplementary materials/S8-Molecular docking and kinetic simulation/Dynamic simulation/gpi-lutdccp.pdf]

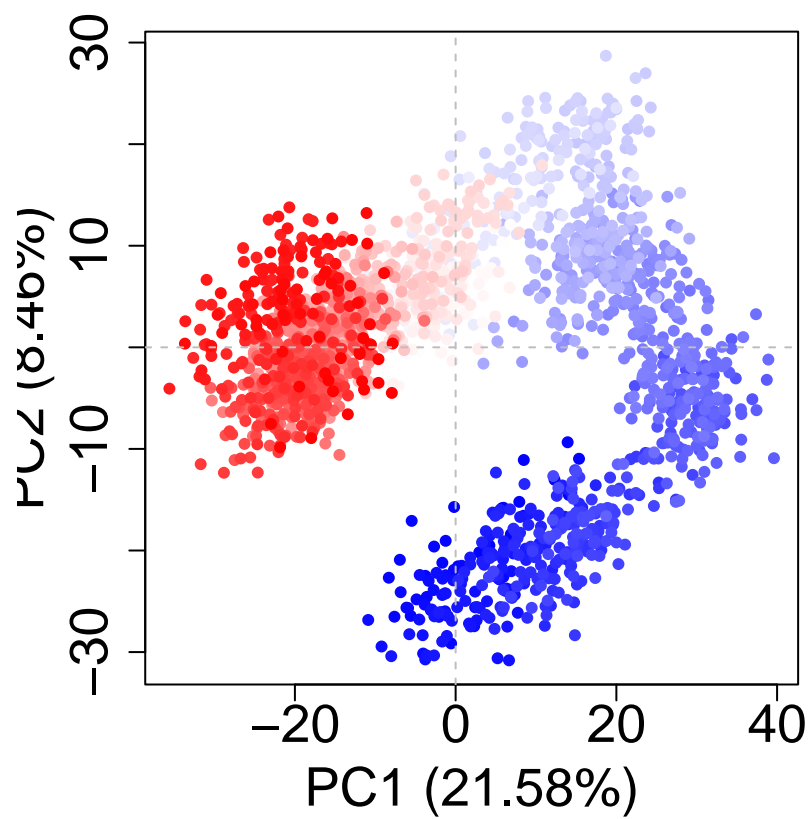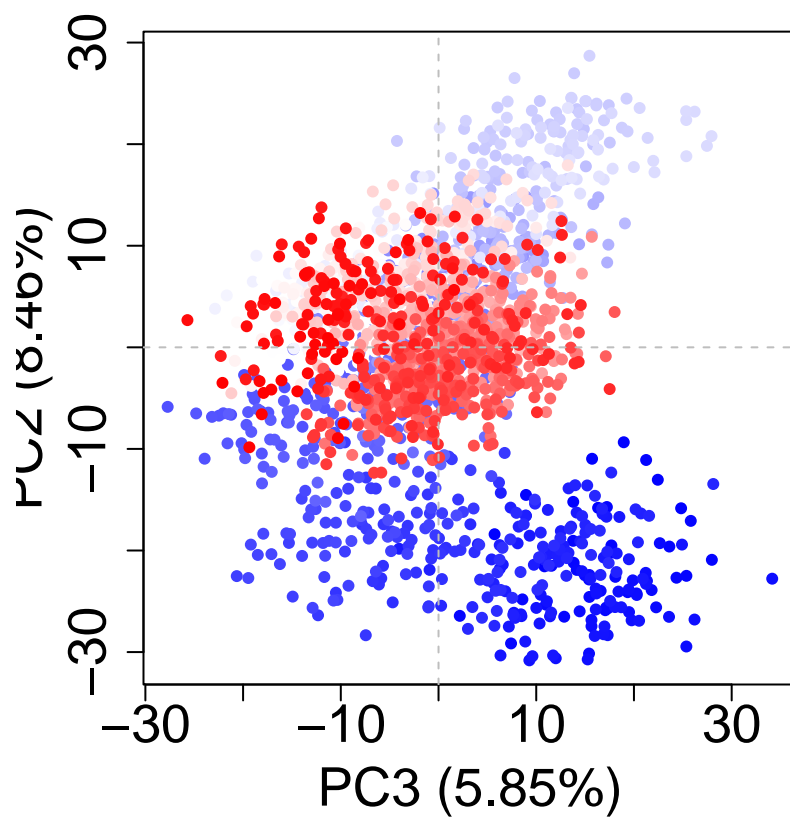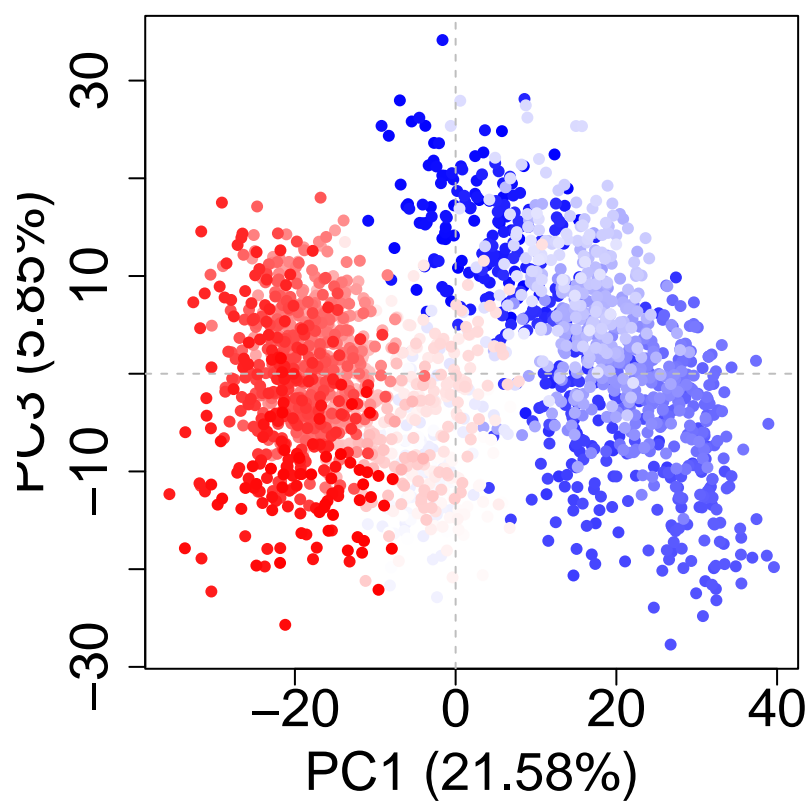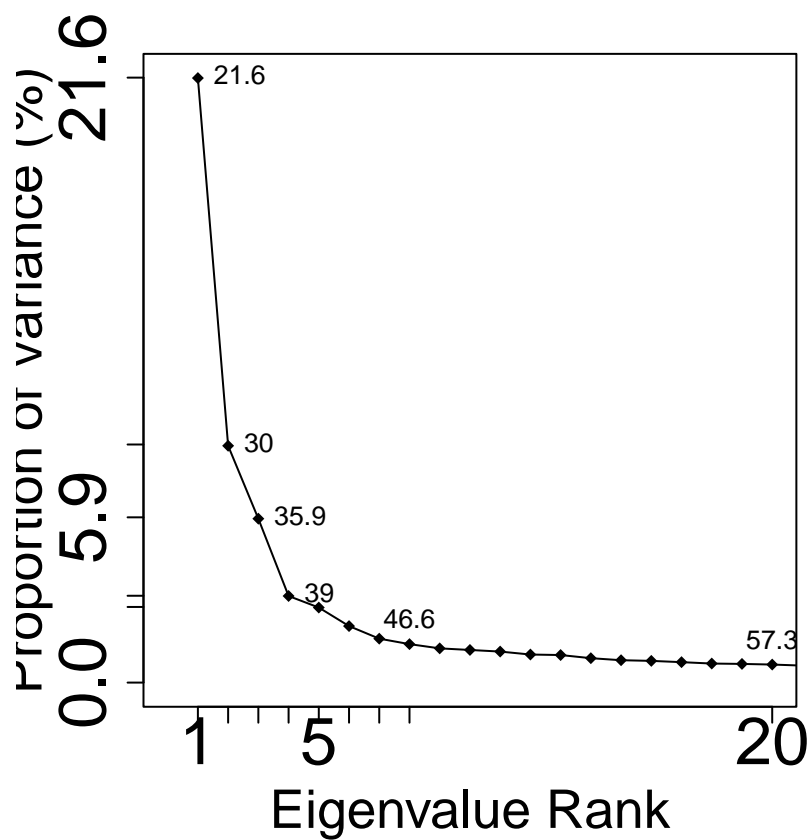

Supplement: Supplementary file 1 — Supporting information. [file IID3-13-e70166-s001.zip › Supplementary materials/S8-Molecular docking and kinetic simulation/Dynamic simulation/gpi-lutpca.pdf]

Residue Cross Correlation

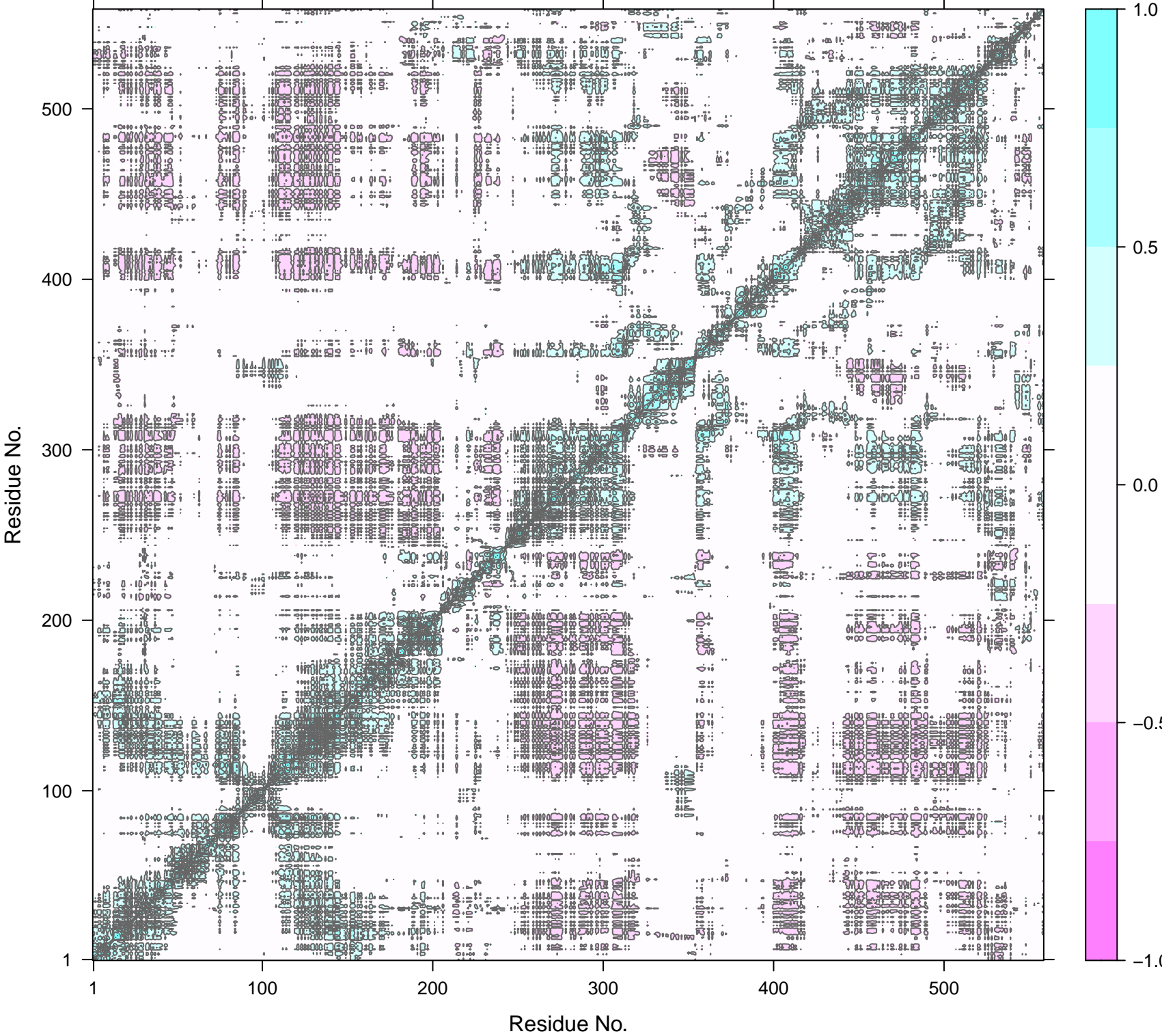

Supplement: Supplementary file 1 — Supporting information. [file IID3-13-e70166-s001.zip › Supplementary materials/S8-Molecular docking and kinetic simulation/Dynamic simulation/gpi-sitdccp.pdf]

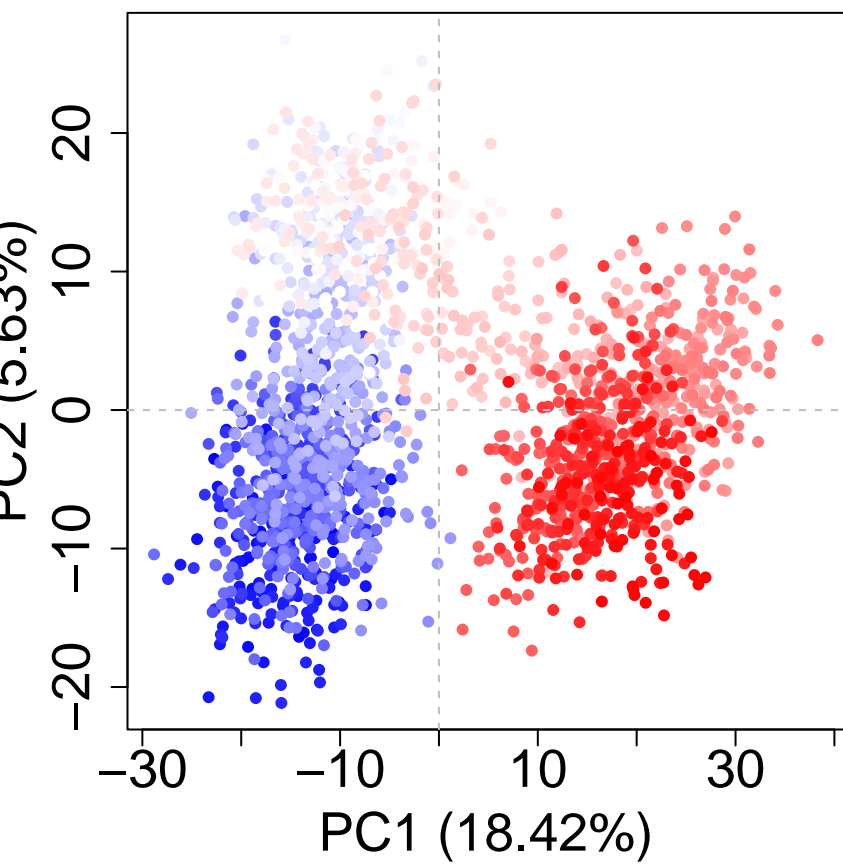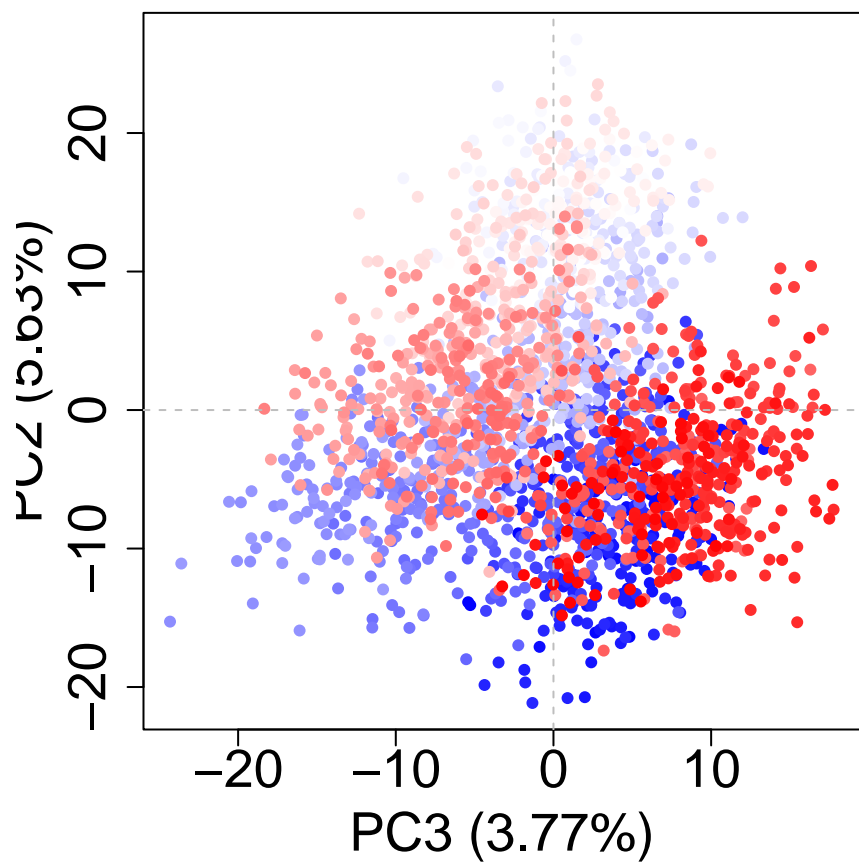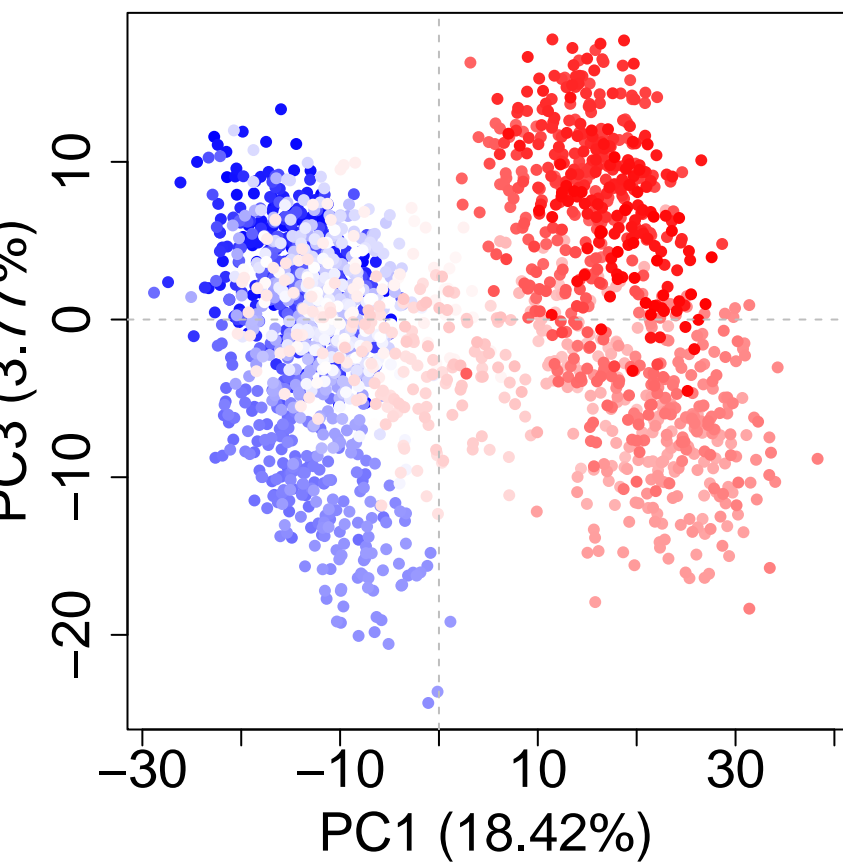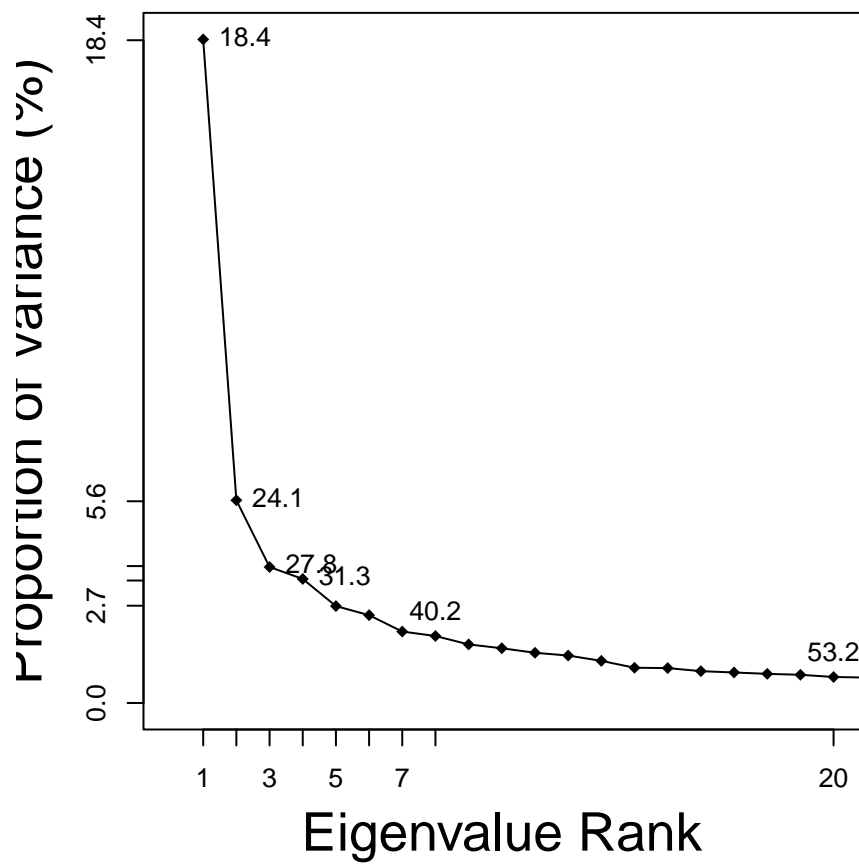

Supplement: Supplementary file 1 — Supporting information. [file IID3-13-e70166-s001.zip › Supplementary materials/S8-Molecular docking and kinetic simulation/Dynamic simulation/gpi-stipca.pdf]

(A)

Residue Cross Correlation

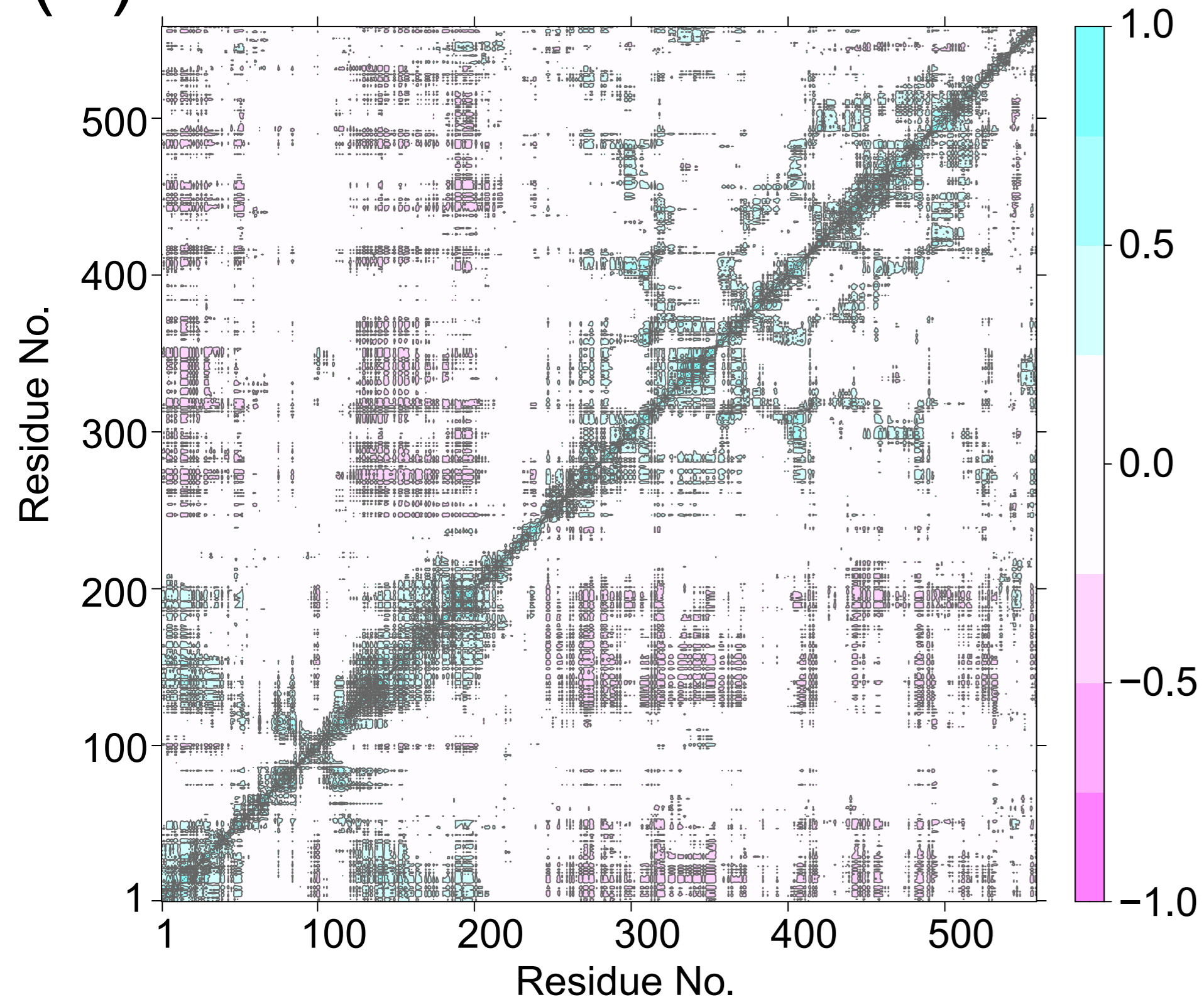

(B)

Residue Cross Correlation

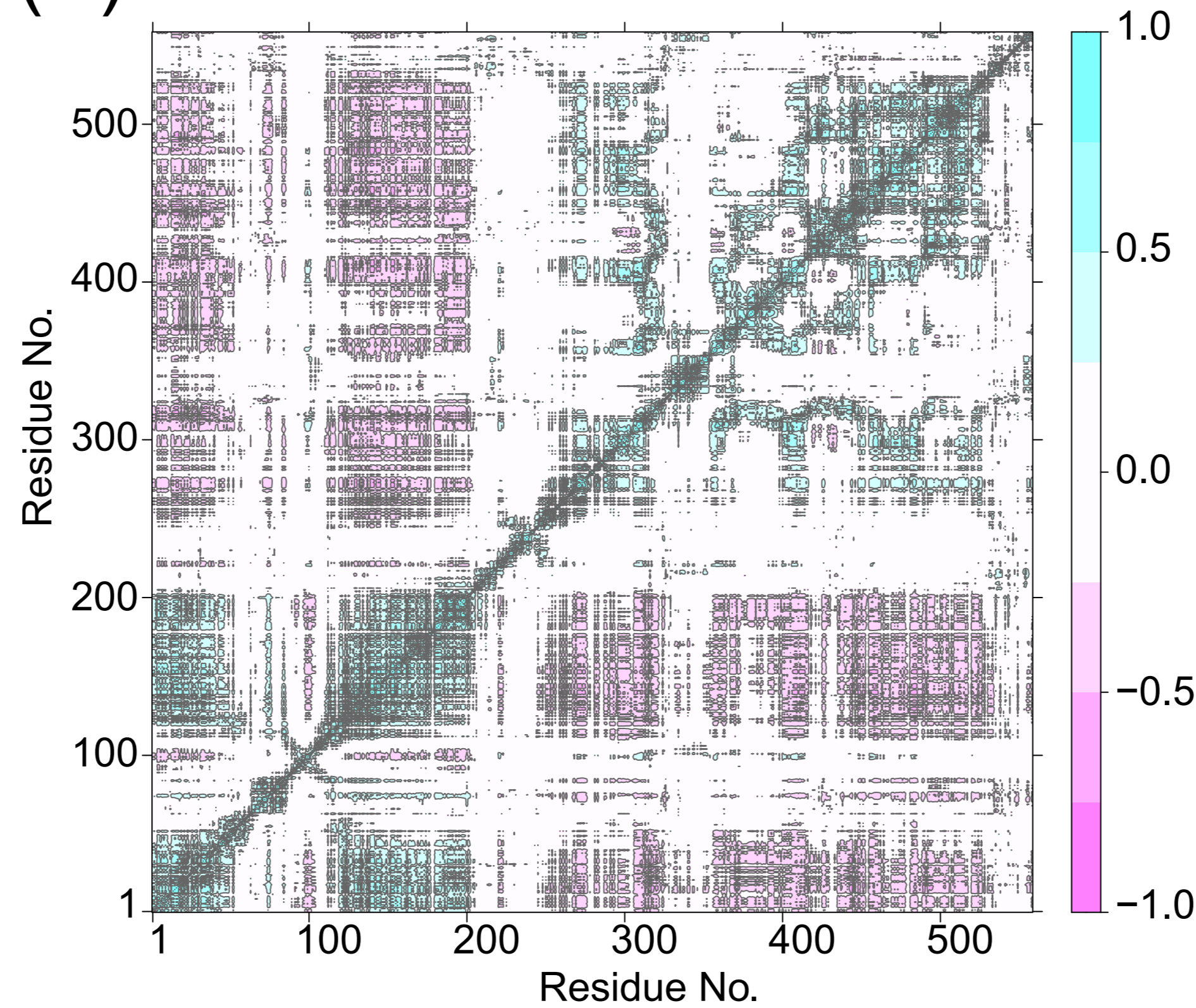

(C)

Residue Cross Correlation

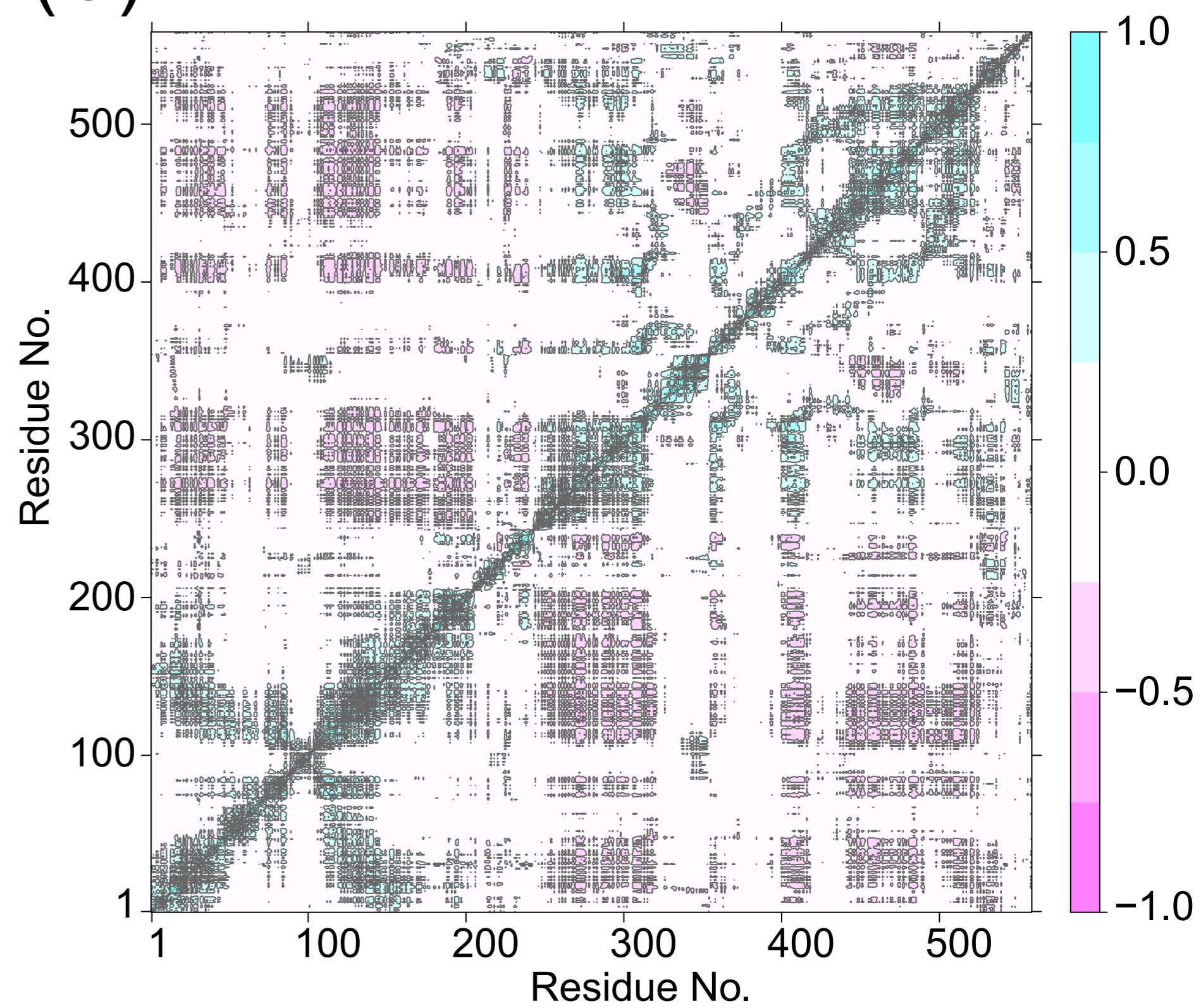

Supplement: Supplementary file 1 — Supporting information. [file IID3-13-e70166-s001.zip › Supplementary materials/S8-Molecular docking and kinetic simulation/Dynamic simulation/merge-dccp.pdf]

(A)

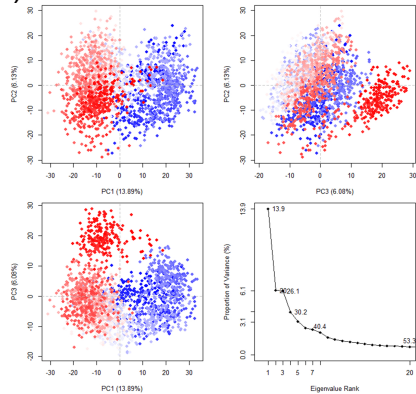

(B)

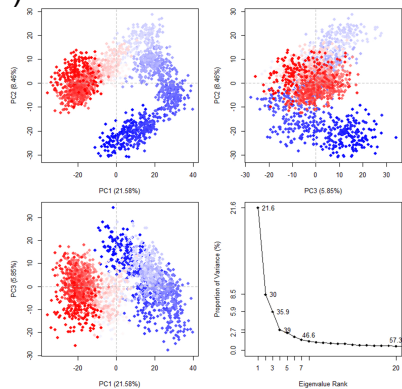

(C)

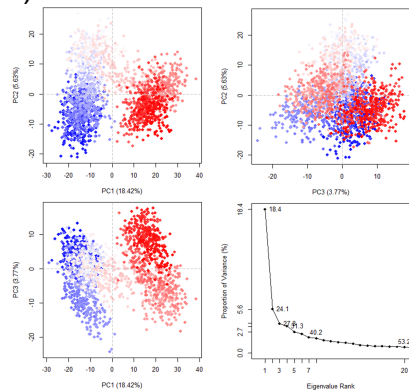

Supplement: Supplementary file 1 — Supporting information. [file IID3-13-e70166-s001.zip › Supplementary materials/S8-Molecular docking and kinetic simulation/Dynamic simulation/merge-pca.pdf]

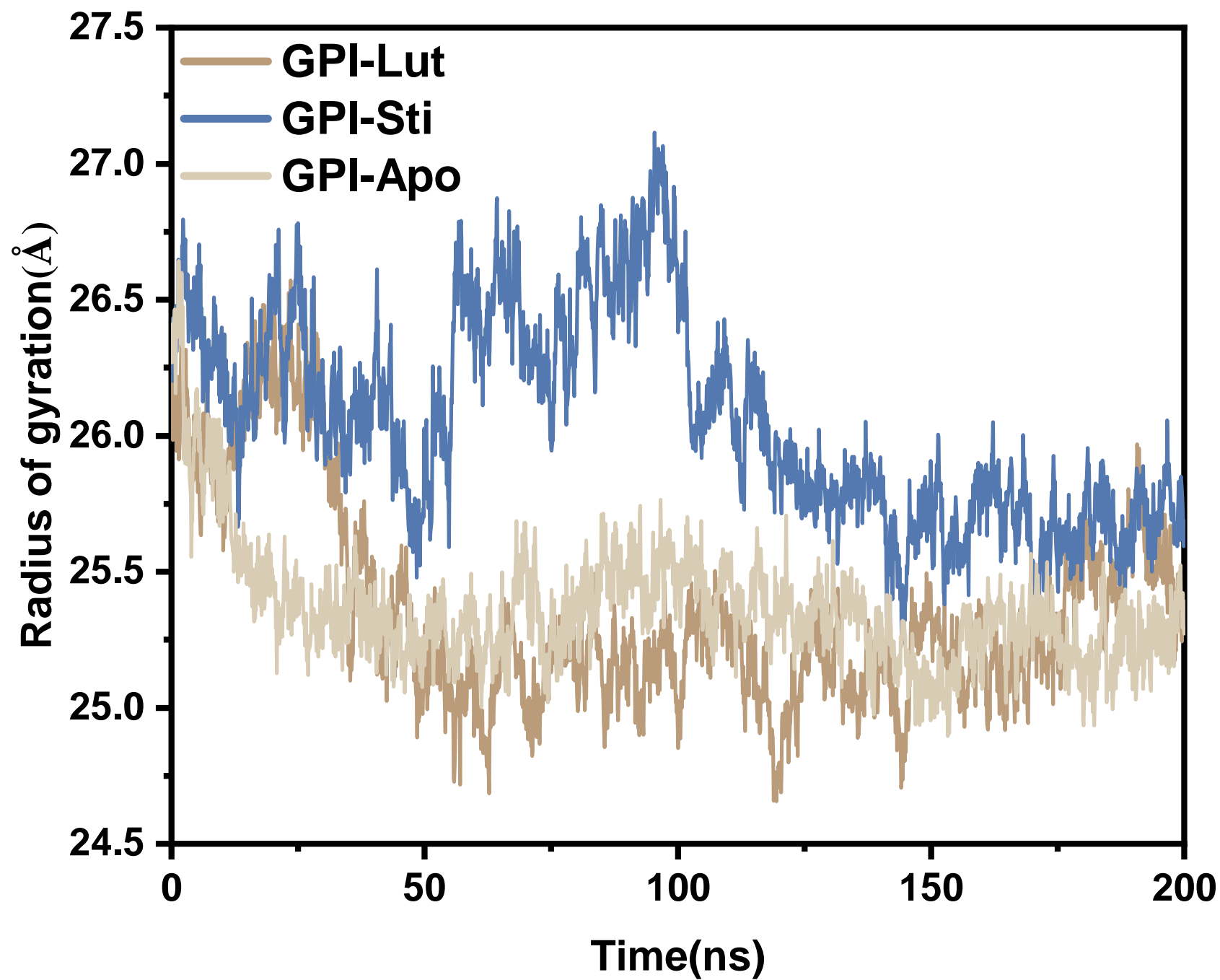

Supplement: Supplementary file 1 — Supporting information. [file IID3-13-e70166-s001.zip › Supplementary materials/S8-Molecular docking and kinetic simulation/Dynamic simulation/RG1.pdf]

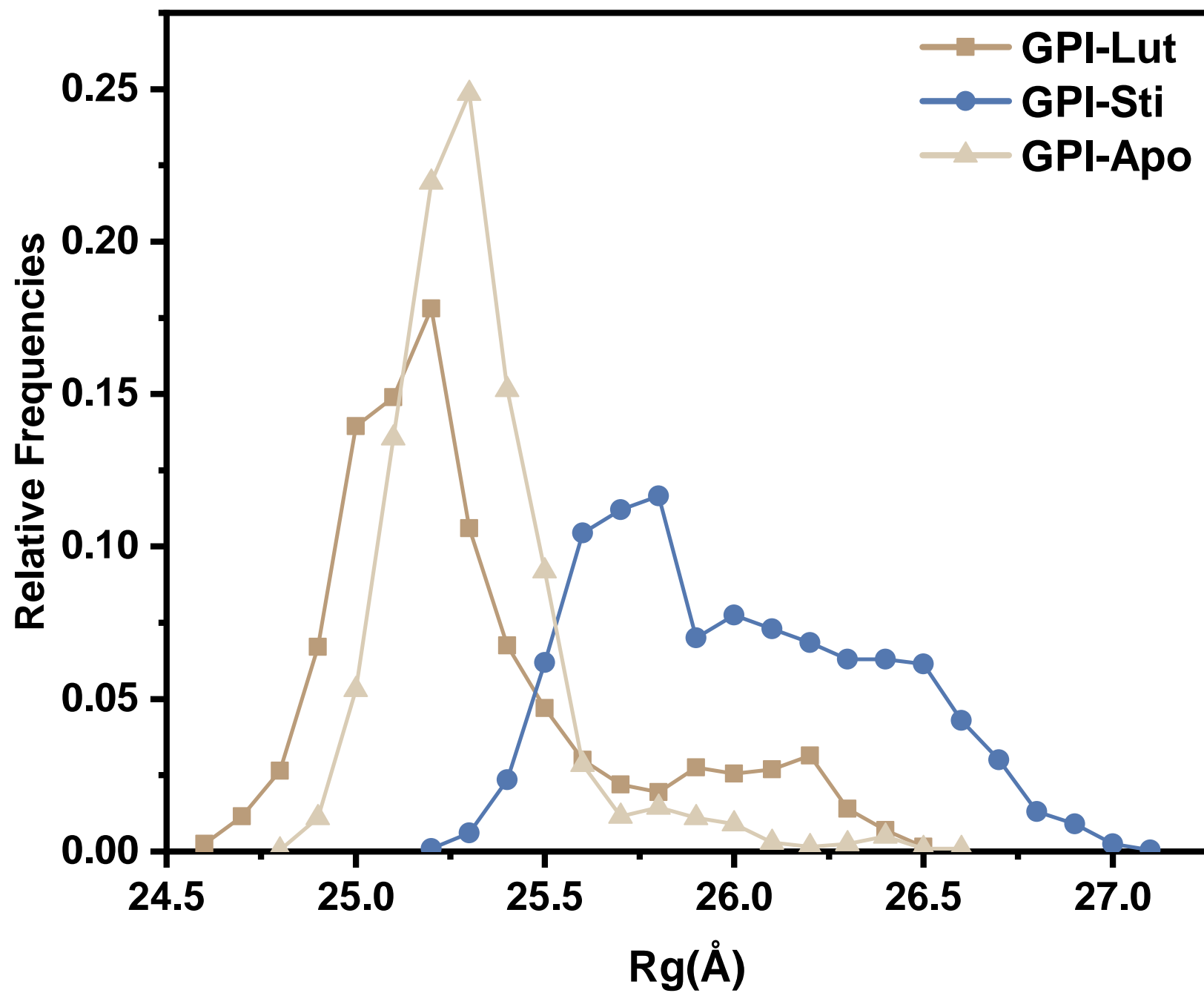

Supplement: Supplementary file 1 — Supporting information. [file IID3-13-e70166-s001.zip › Supplementary materials/S8-Molecular docking and kinetic simulation/Dynamic simulation/RG2.pdf]

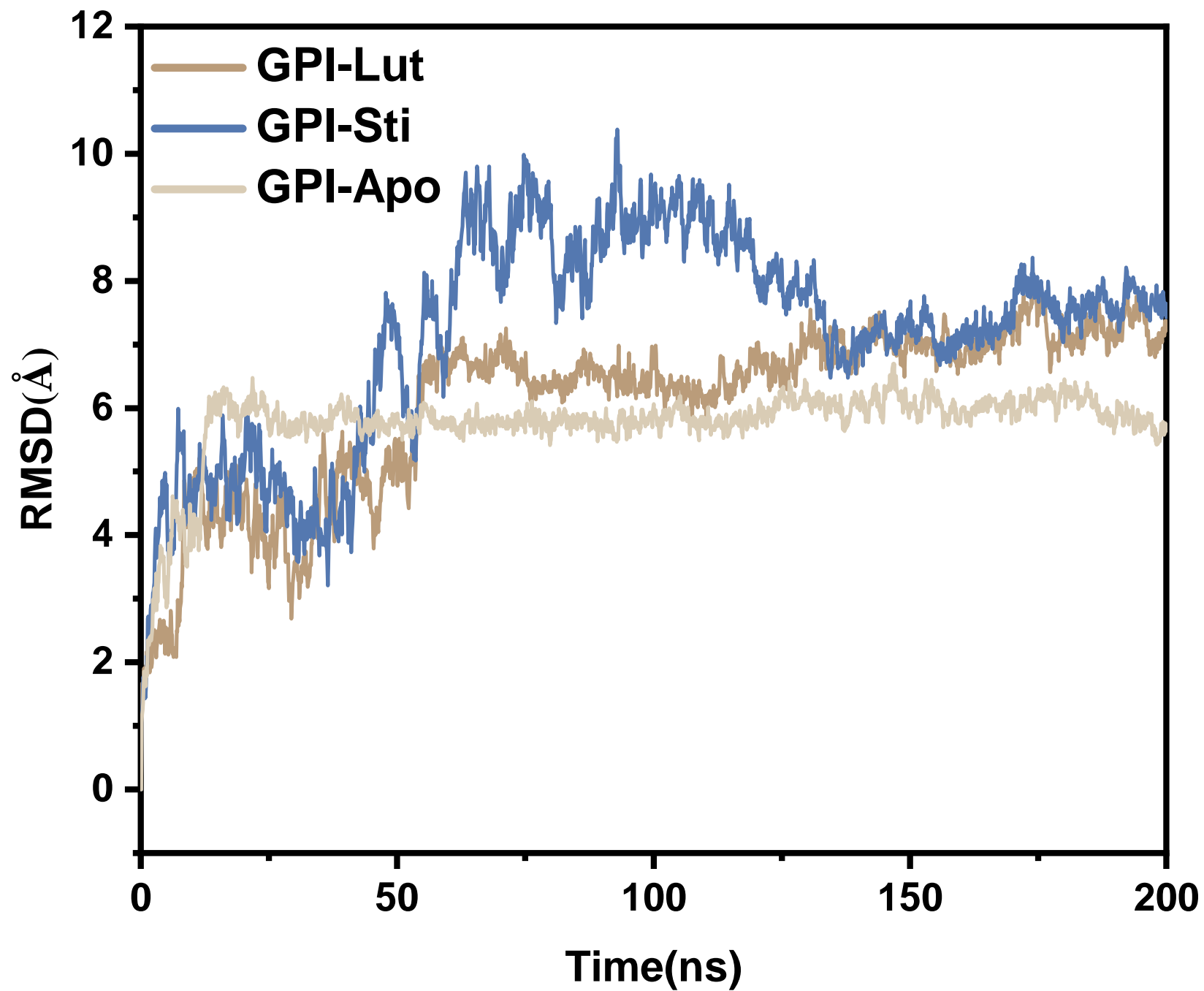

Supplement: Supplementary file 1 — Supporting information. [file IID3-13-e70166-s001.zip › Supplementary materials/S8-Molecular docking and kinetic simulation/Dynamic simulation/RMSD1.pdf]

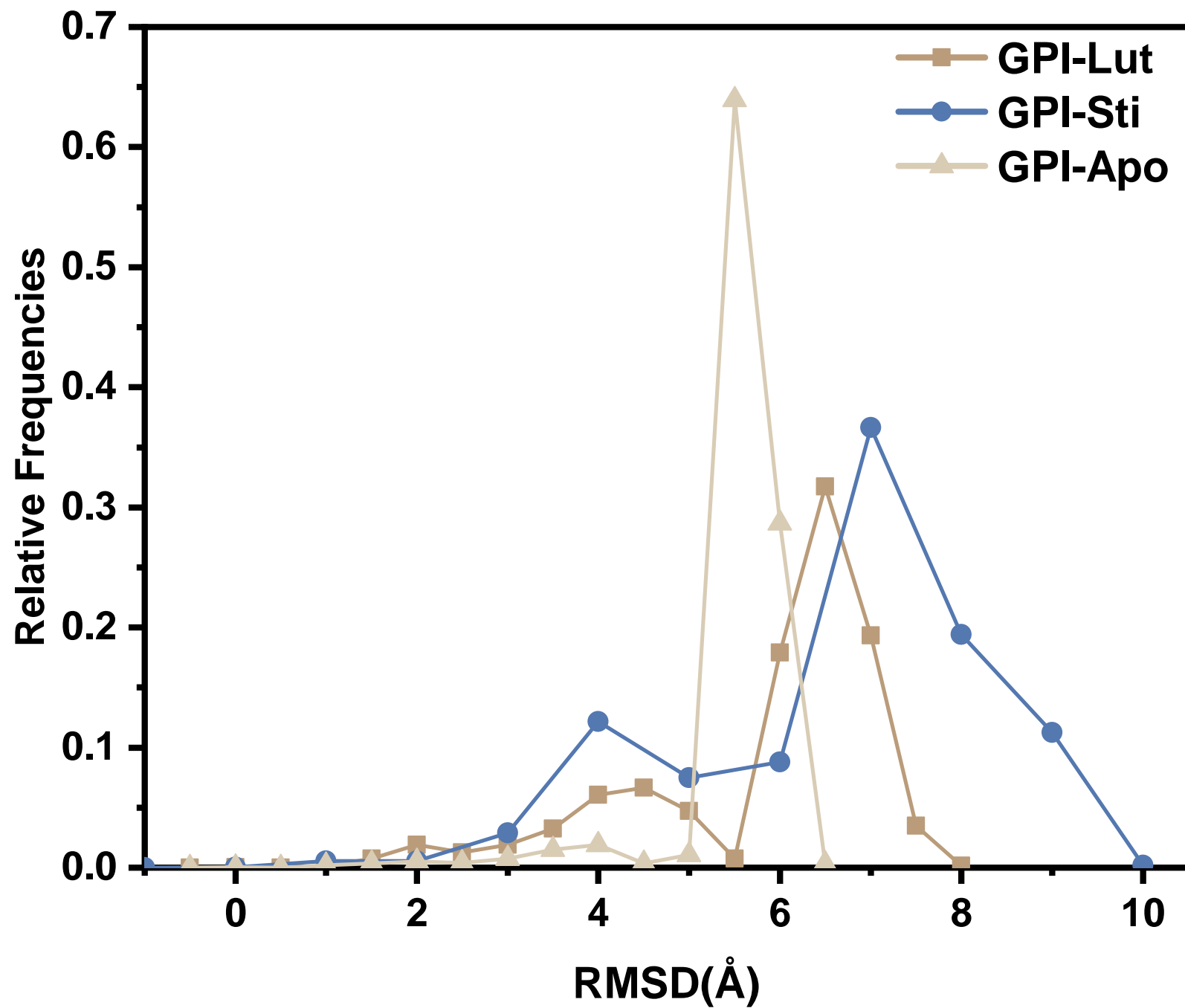

Supplement: Supplementary file 1 — Supporting information. [file IID3-13-e70166-s001.zip › Supplementary materials/S8-Molecular docking and kinetic simulation/Dynamic simulation/RMSD2.pdf]

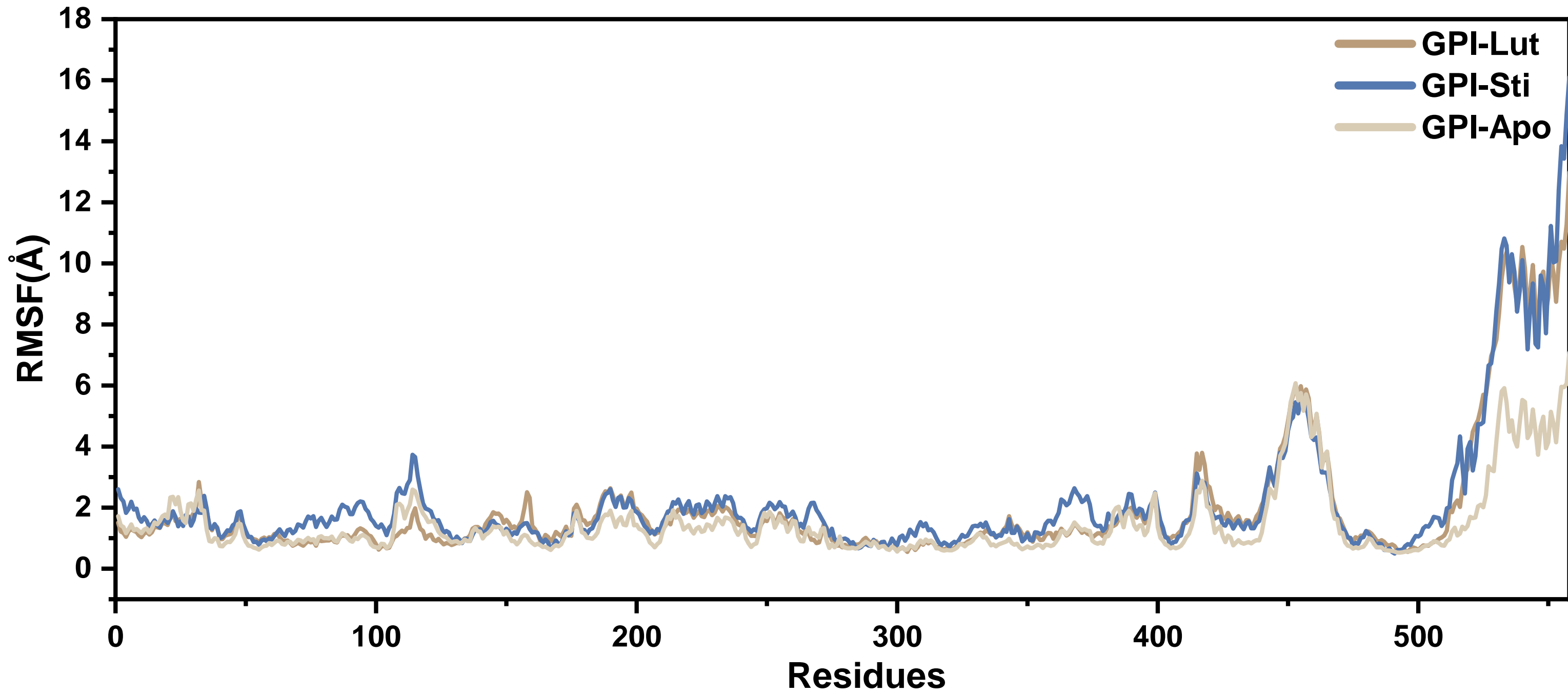

Supplement: Supplementary file 1 — Supporting information. [file IID3-13-e70166-s001.zip › Supplementary materials/S8-Molecular docking and kinetic simulation/Dynamic simulation/RMSF.pdf]

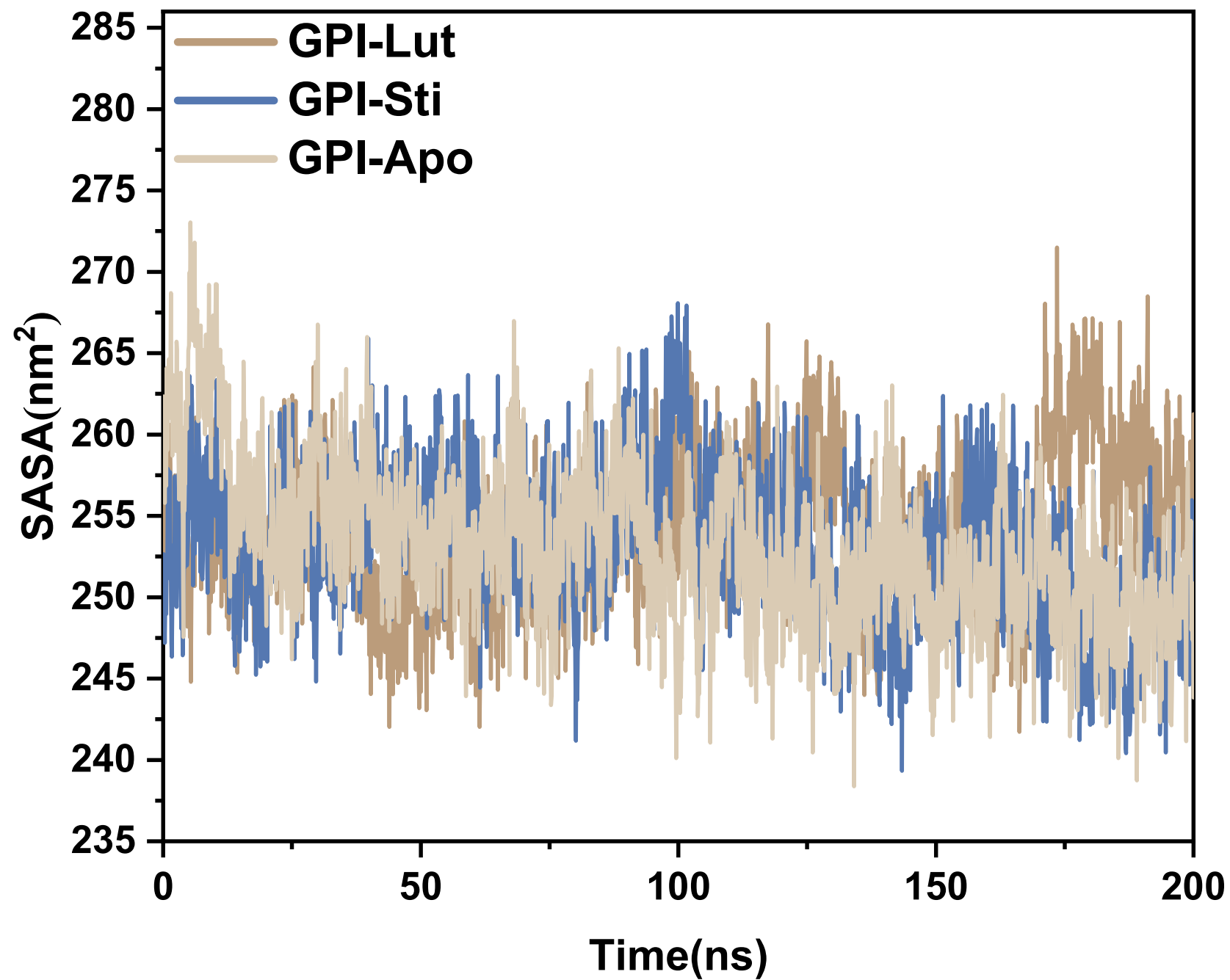

Supplement: Supplementary file 1 — Supporting information. [file IID3-13-e70166-s001.zip › Supplementary materials/S8-Molecular docking and kinetic simulation/Dynamic simulation/SASA_1.pdf]

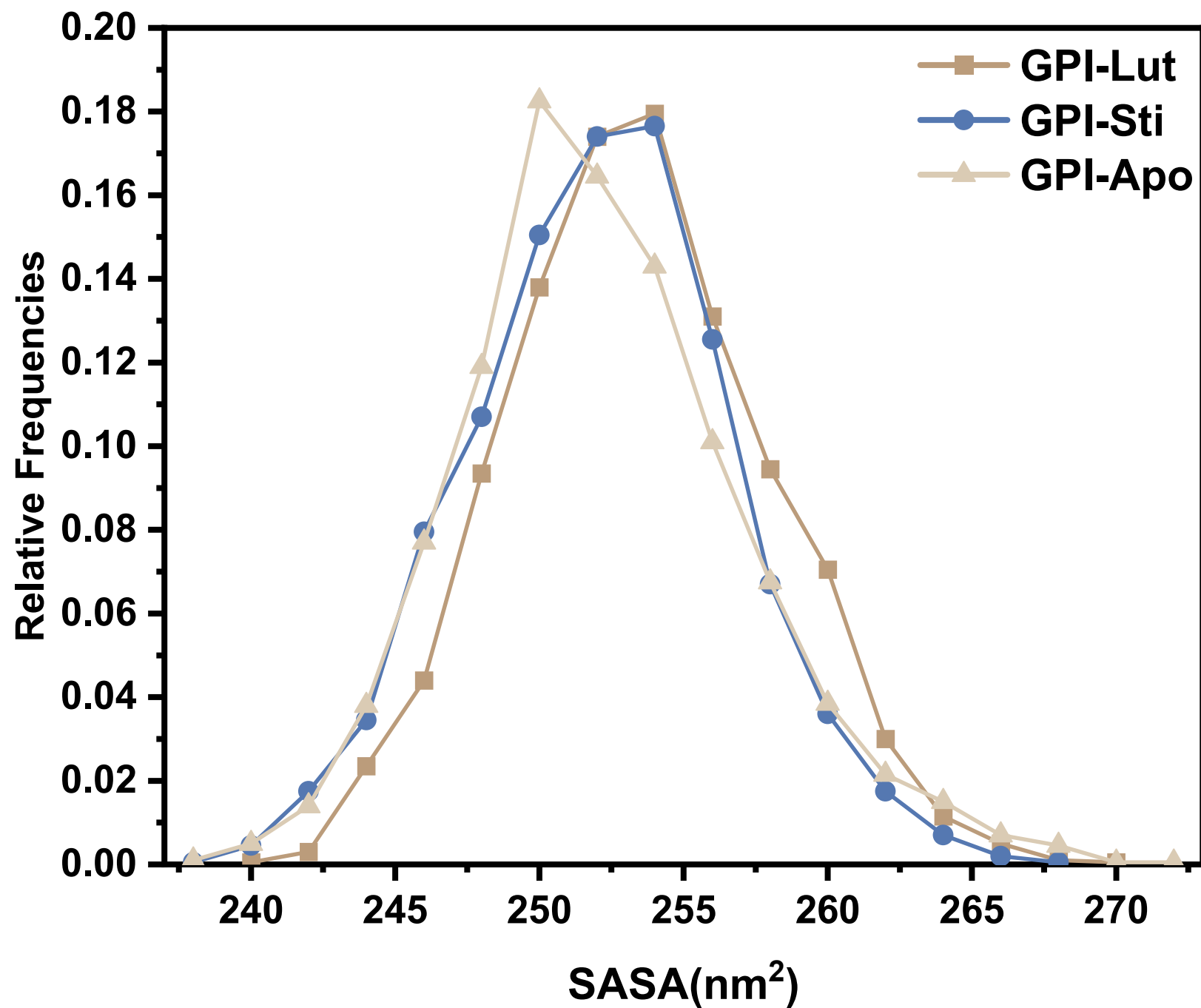

Supplement: Supplementary file 1 — Supporting information. [file IID3-13-e70166-s001.zip › Supplementary materials/S8-Molecular docking and kinetic simulation/Dynamic simulation/SASA_2.pdf]

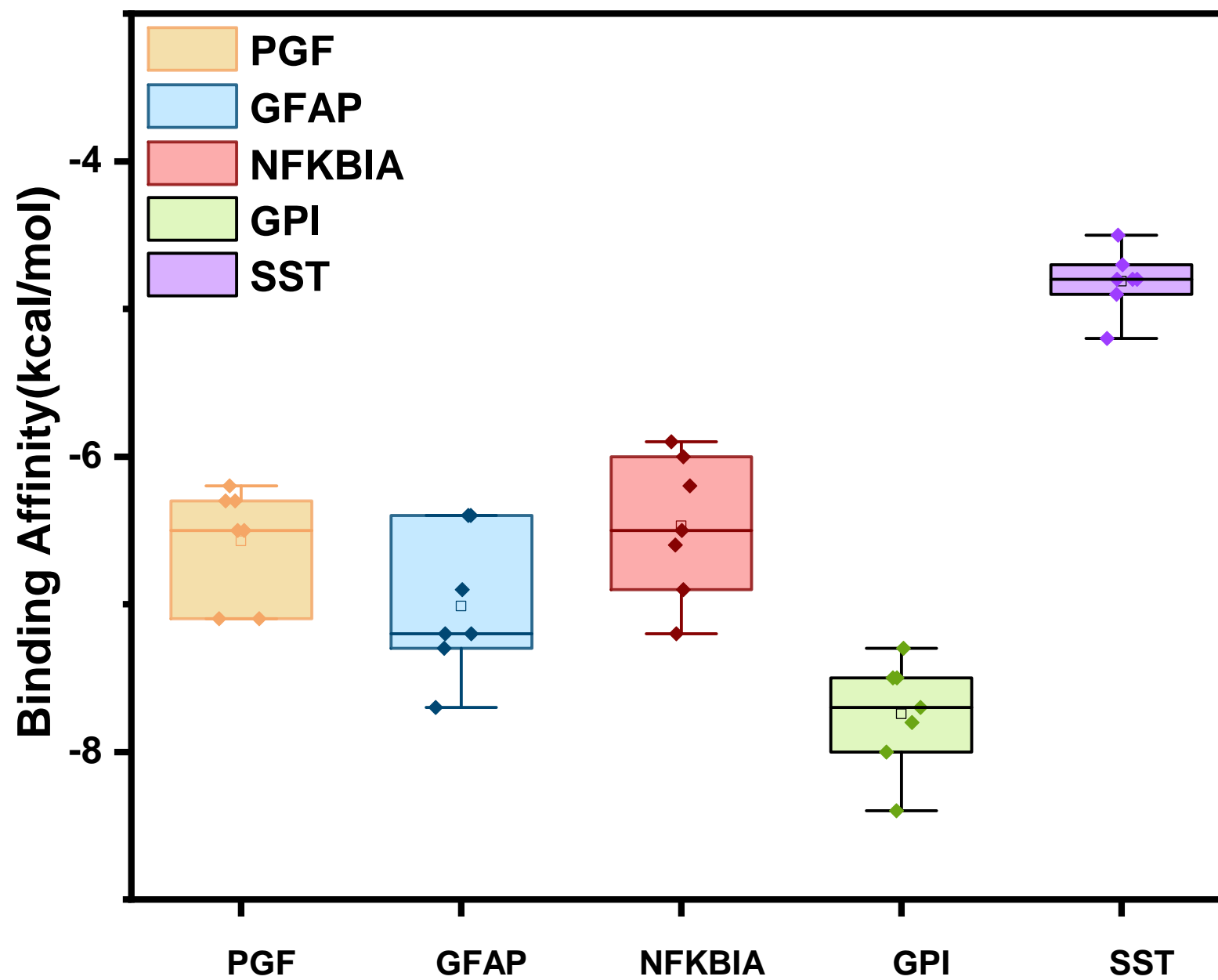

Supplement: Supplementary file 1 — Supporting information. [file IID3-13-e70166-s001.zip › Supplementary materials/S8-Molecular docking and kinetic simulation/Molecular docking/Docking affinity value.pdf]
